# Association between CD40 rs1883832 and immune-related diseases susceptibility: A meta-analysis

**DOI:** 10.18632/oncotarget.18704

**Published:** 2017-06-28

**Authors:** Jiaxuan Qin, Jinchun Xing, Rongfu Liu, Bin Chen, Yuedong Chen, Xuan Zhuang

**Affiliations:** ^1^ Department of Urology Surgery, The First Affiliated Hospital of Xiamen University, Xiamen, Fujian, 361003, China; ^2^ Center of Diagnosis and Treatment of Urinary System Diseases, The First Affiliated Hospital of Xiamen University, Xiamen, Fujian, 361003, China; ^3^ The Key Laboratory of Urinary Tract Tumors and Calculi, The First Affiliated Hospital of Xiamen University, Xiamen, Fujian, 361003, China

**Keywords:** CD40, rs1883832, immune-related disease, meta-analysis, SNP

## Abstract

**Background/objective:**

It has been reported that CD40 rs1883832 might be associated with immune-related diseases susceptibility. Owing to mixed and inconclusive results, we conducted a meta-analysis of case–control studies to summarize and clarify this association.

**Methods/main results:**

A systematic search of studies on the association between CD40 rs1883832 and immune-related diseases susceptibility was conducted in databases. Odds ratios and 95% confidence intervals were used to pool the effect size. 40 articles were included in our meta-analysis.

**Conclusions:**

CD40 rs1883832 is associated with decreased risk of Graves’ disease, especially in Asian; CD40 rs1883832 is associated with increased risk of multiple sclerosis; CD40 -1C>T (rs1883832) is not associated with the susceptibility of Hashimoto's thyroiditis, systemic sclerosis or Asthma; there is insufficient data to fully confirm the association between CD40 rs1883832 and systemic lupus erythematosus (SLE), rheumatoid arthritis (RA), Behçet's disease (BD), myasthenia gravis (MG), Crohn's disease (CD), ulcerative colitis (UC), Sarcoidosis, Fuch uveitis syndrome (FUS), Vogt-Koyanagi-Harada syndrome (VKH), Kawasaki disease (KD), giant cell arteritis (GCA) or Immune thrombocytopenia (ITP).

## INTRODUCTION

CD40, expressed by some immune and non-immune cells, is a tumor necrosis factor receptor that plays a critical role in adaptive immunity. CD40 involves in B cell proliferation, the generation of plasma cells, B cell memory and the development of immature dendritic cells. [1–3] CD40 is an attractive candidate receptor for contributing to a variety of immune-related processes in which B and T cell activation play a role in pathogenesis [4].

CD40 -1C>T (rs1883832) is a functional single nucleotide polymorphism (SNP) located at -1 from the start codon of CD40 gene. The rs1883832T decreased the translational efficiency of CD40 transcripts, resulting in less CD40 protein level [5].

Association between CD40 rs1883832 and immune-related diseases susceptibility has been studied in several populations. Sample sizes in these studies are relatively small. In Graves’ disease and Hashimoto's thyroiditis, meta-analysis has been performed before, [6, 7] however new studies and more immune-related diseases need to be involved. Therefore, we decided to perform a meta-analysis of case–control studies to estimate it.

## MATERIALS AND METHODS

### Identification of eligible studies

A systematic search in PubMed, Embase, Cochrane Library, clinicaltrials.gov, CNKI (China National Knowledge Infrastructure), WanFangData (one China database) and CQVIP (one China database) databases were carried out by two independent investigators. The following terms were used: “CD40 OR TNFRSF5 OR Bp50” AND “rs1883832 OR C/T-1 OR C>T-1 OR -1C/T OR -1C>T OR C-1T OR Kozak OR 5′-untranslated region OR 5′-utr”, without any limitation applied. The last search update was performed on August 4, 2016. References of related studies and reviews were also manually searched for additional studies. GWAS were searched in Immunobase.

### Inclusion and exclusion criteria

Studies selected in this meta-analysis must meet the following inclusion criteria: (1) evaluation of the association between CD40 rs1883832 and immune-related diseases; (2) case-control study; (3) studies focusing on tissues of human beings; (4) detailed genotype data could be acquired to calculate the odds ratios (ORs) and 95% confidence intervals (95%CIs). Exclusion criteria: (1) duplication of previous publications (When there were multiple publications from the same population, only the largest study was included); (2) comment, review and editorial; (3) study without detailed genotype data; (4) GWAS; (5) studies focusing on cell lines. Dissertation thesis were included in the analysis.

Study selection was achieved by two investigators independently, according to the inclusion and exclusion criteria by screening the title, abstract and full-text. Any dispute was solved by discussion.

### Data extraction

Two investigators extracted data of the eligible studies independently. In the case of a conflict, an agreement was reached by discussion. If the dissent still existed, the third investigator would be involved to adjudicate the disagreements. Try to contact the author by email for detailed genotype data.

The following contents were collected: first author's surname, year of publication, disease type, the characteristics of cases and controls, source of control groups, country of origin, the detective sample, ethnicity, genotyping method, Hardy-Weinberg equilibrium, number of cases and controls for each genotype.

### Methodological quality assessment

The qualities of included studies were evaluated independently by two investigators according to Newcastle-Ottawa Scale (NOS) [8] and the most important factor was ”age, gender and country”. Quality scores range from 0 to 9, and higher scores means better quality of the study. Disagreement was resolved through discussion.

### Statistics analysis

Our meta-analysis was conducted according to the PRISMA checklists. [9] Hardy-Weinberg equilibrium (HWE) was evaluated for each study by Chi-square test in control groups, and *P* < 0.05 was considered as a significant departure from HWE. OR and 95% CIs were calculated to evaluate the strength of the association between CD40 rs1883832 and immune-related diseases. Pooled ORs were obtained from combination of single studies by allelic comparison (T vs C), dominant model (CT+TT vs CC), recessive model (TT vs CC+CT), homozygote comparison (TT vs CC) and heterozygote comparison (CT vs CC), respectively. The statistical significant level was determined by *Z*-test with *P* value less than 0.05.

Heterogeneity was evaluated by *Q*-test and I^2^ index. [10] When *Q*-test's *P*-value was less than 0.10 and/or I^2^ index was more than 50%, the random-effects model (DerSimonian and Laird method) was used; otherwise, the fixed-effects model (Mantel and Haenszel method) was conducted. [11] Sensitivity analyses were performed towards each genetic model to evaluate effect of each study on combined ORs by sequentially excluding each study in total and in any subgroup including more than two studies. Besides, subgroup analyses were stratified by disease, ethnicity (Caucasian and Asian), control type (population-based and hospital-based) and HWE (*P* < 0.05 and *P* ≥ 0.05). Potential publication bias was checked by Begg's funnel [12] plots and Egger's test. [13] An asymmetric plot, the *P* value of Begg's test (P_B_) less than 0.05, and the *P* value of Egger's test (P_E_) less than 0.05 was considered a significant publication bias. All statistical analyses were performed with Stata 12.0 software (StataCorp, College Station, Texas, USA). A two-tailed *P* < 0.05 was considered significant except for specified conditions, where a certain *P* value was declared.

## RESULTS

### Characteristics of studies

**Figure 1:**
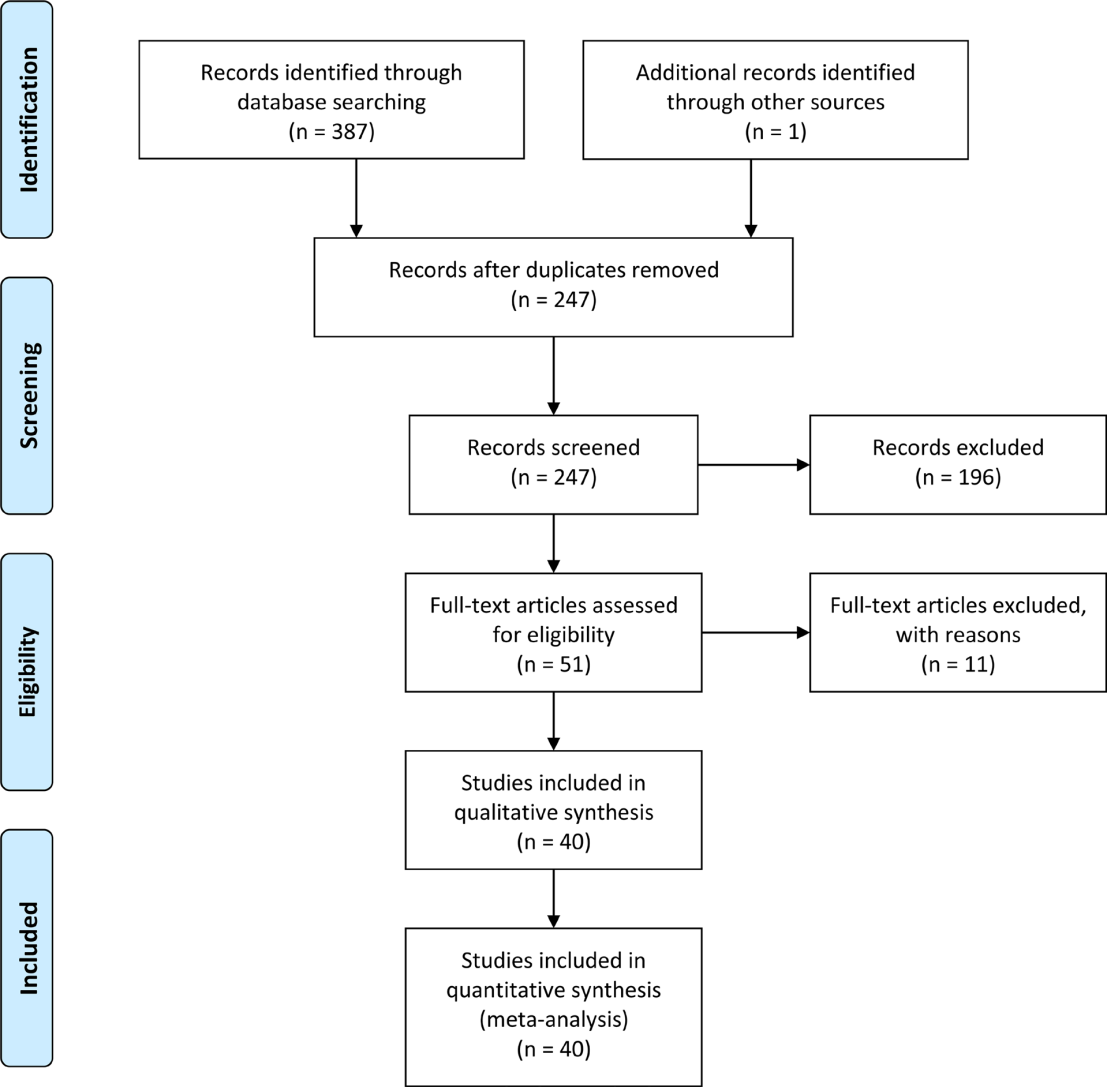
Flow chart of study selection

A total of 388 articles were acquired from databases (PubMed = 78, Embase = 98, Cochrane = 2, clinicaltrials.gov = 0, CNKI = 118, WanFangData = 79, CQVIP = 12, other sources (from manually search) = 1 [14]). The selection process was shown in Figure 1. 11 full-text articles were excluded (2 duplicate study [63, 64]; 3 detailed genotype data could not be extracted and might from the same population of another study [14–16]; 4 improper full text [17–20]; 2 might from the same population of a larger study [21, 22]). Finally, 40 articles [23–62] were included in our meta-analysis. The characteristics of each study were shown in Supplementary Table 1. Different genotyping methods were utilized including sequencing, PCR–RFLP, PCR-HRM, TaqMan PCR, MassARRAY. Blood samples were used for genotyping in all studies.

### Overall analyses and subgroup analyses

Summary results of each genetic model were listed in Table 1. Significantly decreased risk of immune-related diseases was found in almost all genetic models of GD (Graves’ disease) and its subgroups except for homozygote comparison (TT vs CC) and recessive model (TT vs CC+CT) of GD's Caucasian subgroup. Significantly increased risk was found in all genetic models of MS (multiple sclerosis) and its subgroups. Overall, significantly decreased risk of immune-related diseases was found in allelic comparison (T vs C) and in several subgroups. No statistically significant changes of immune-related diseases risk was found in other analyses. (Detailed in Table 1).

**Table 1:** Summary of pooled ORs in the meta-analysis

	Number (cases/controls)	T vs C		TT vs CC		CT vs CC		CT+TT vs CC		TT vs CC+CT	
		OR^*^ (95%CI^*^)	I^2^ (%)	OR (95%CI)	I^2^ (%)	OR (95%CI)	I^2^ (%)	OR (95%CI)	I^2^ (%)	OR (95%CI)	I^2^(%)
GD^*^	5006/4537	0.738 (0.664–0.820)^*^	**60.1**	**0.537 (0.424–0.679)**	**57.3**	**0.786 (0.719–0.859)**	29.9	**0.706 (0.619–0.806)**	**51.9**	**0.657 (0.577–0.748)**	46.2
**H**WE^*^	4560/4171	**0.775 (0.697–0.861)**	**55.8**	**0.623 (0.535–0.727)**	49.7	**0.809 (0.738–0.886)**	24.2	**0.774 (0.709–0.845)**	46.4	**0.696 (0.604–0.801)**	35.2
**P**B^*^	4746/4289	**0.744 (0.666–0.830)**	**60.7**	**0.541 (0.420–0.696)**	**58.7**	**0.788 (0.720–0.863)**	33.5	**0.712 (0.620–0.817)**	**53.2**	**0.670 (0.584–0.769)**	48.0
**C**aucasian	2247/2297	**0.832 (0.697–0.992)**	**60.4**	0.745 (0.462–1.201)	**53.5**	**0.816 (0.721–0.923)**	0.8	**0.814 (0.722–0.917)**	43.0	0.863 (0.662–1.125)	45.3
**A**sian	2759/2240	**0.700 (0.619–0.791)**	**53.5**	**0.507 (0.428–0.600)**	48.0	**0.755 (0.665–0.858)**	39.9	**0.653 (0.546–0.781)**	**51.5**	**0.602 (0.518–0.699)**	34.6
HWE	2313/1874	**0.743 (0.652–0.847)**	**50.7**	**0.553 (0.459–0.667)**	34.8	**0.800 (0.698–0.917)**	38.8	**0.731 (0.642–0.831)**	49.6	**0.638 (0.540–0.754)**	9.7
PB	2499/1992	**0.703 (0.615–0.804)**	**56.2**	**0.482 (0.367–0.633)**	**51.9**	**0.758 (0.663–0.866)**	44.8	**0.654 (0.538–0.796)**	**54.9**	**0.609 (0.518–0.716)**	39.5
HT^*^	576/604	1.079 (0.914–1.274)	0.0	1.171 (0.825–1.661)	0.0	1.121 (0.870–1.445)	0.0	1.129 (0.888–1.435)	0.0	1.069 (0.781–1.463)	0.0
MS^*^	3851/4368	**1.175 (1.093–1.263)**	23.0	**1.329 (1.117–1.582)**	29.3	**1.189 (1.081–1.309)**	0.0	**1.213 (1.107–1.329)**	0.0	***1.239 (1.047–1.467)***	20.9
**H**WE	2321/1471	**1.249 (1.120–1.393)**	0.0	**1.573 (1.195–2.071)**	4.3	***1.227 (1.066–1.414)***	0.0	**1.277 (1.116–1.460)**	0.0	***1.458 (1.114–1.908)***	0.0
**P**B	2172/3489	**1.162 (1.067–1.265)**	39.7	***1.302 (1.066–1.591)***	45.3	***1.178 (1.050–1.321)***	0.0	**1.199 (1.075–1.338)**	0.0	***1.217 (1.002–1.477)***	38.8
SSc^*^	2605/3138	1.022 (0.940–1.111)	0.0	1.086 (0.888–1.328)	40.8	0.994 (0.890–1.110)	27.1	1.009 (0.908–1.120)	0.0	1.099 (0.793–1.523)	**56.9**
SLE^*^	905/1307	1.115 (0.808–1.537)	**78.5**	1.249 (0.695–2.245)	**72.5**	*1.042 (0.570–1.903)*	84.3	1.093 (0.611–1.956)	**85.1**	1.183 (0.927–1.509)	0.2
**P**B	700/1087	0.950 (0.620–1.455)	**77.4**	0.963 (0.534–1.734)	**54.0**	0.779 (0.284–2.139)	89.6	0.817 (0.333–2.004)	**88.4**	1.059 (0.793–1.413)	0.0
**Asthma**	675/333	0.980 (0.807–1.189)	2.1	0.903 (0.602–1.354)	0.0	1.007 (0.555–1.826)	71.5	0.978 (0.592–1.614)	**64.6**	0.911 (0.630–1.319)	0.0
RA^*^	1722/2021	0.949 (0.792–1.138)	**52.5**	0.887 (0.563–1.397)	**60.2**	0.942 (0.821–1.081)	0.0	0.918 (0.805–1.046)	11.6	0.890 (0.604–1.313)	52.9
BD^*^	658/627	1.062 (0.796–1.416)	**67.1**	1.189 (0.640–2.209)	**66.5**	0.857 (0.673–1.091)	0.0	0.959 (0.765–1.203)	0.0	1.284 (0.661–2.492)	**74.3**
**Overall**	19482/24998	***0.932 (0.873–0.994)***	**74.6**	*0.873 (0.760–1.002)*	**70.1**	0.944 (0.875–1.017)	**59.9**	*0.924 (0.852–1.001)*	**69.7**	0.918 (0.821–1.027)	**60.6**
**H**WE	15471/15941	*0.943 (0.880–1.010)*	**70.1**	0.905 (0.781–1.047)	**65.3**	0.940 (0.869–1.017)	**52.7**	*0.929 (0.853–1.012)*	**64.2**	0.947 (0.841–1.068)	**55.0**
**P**B	17126/23175	**0.921 (0.861–0.984)**	**72.6**	***0.851 (0.739–0.981)***	**68.0**	*0.927 (0.858–1.002)*	**58.8**	***0.908 (0.836–0.986)***	**67.9**	*0.905 (0.805–1.017)*	**58.9**
**C**aucasian	12919/18426	1.019 (0.948–1.095)	**66.9**	1.073 (0.918–1.254)	**55.4**	1.010 (0.931–1.095)	**54.5**	1.015 (0.932–1.106)	**62.7**	*1.057 (0.968–1.154)*	48.3
HWE	9354/9735	0.993 (0.910–1.085)	**65.3**	1.051 (0.860–1.286)	**57.0**	0.968 (0.910–1.029)	42.1	0.975 (0.883–1.075)	**56.7**	1.070 (0.891–1.284)	**50.7**
PB	11240/17547	1.007 (0.935–1.085)	**65.6**	1.051 (0.893–1.237)	**55.3**	0.996 (0.916–1.083)	**53.1**	1.001 (0.917–1.093)	**61.2**	1.041 (0.950–1.140)	48.9
**A**sian	6563/6572	**0.863 (0.775–0.961)**	**76.0**	**0.747 (0.604–0.923)**	**73.2**	***0.870 (0.762–0.994)***	**61.1**	**0.834 (0.722–0.963)**	**71.1**	**0.820 (0.696–0.965)**	**63.6**
HWE	6117/6206	*0.904 (0.814–1.004)*	**72.9**	*0.819 (0.667–1.006)*	**68.8**	0.911 (0.798–1.040)	**59.1**	*0.884 (0.767–1.019)*	**68.6**	*0.878 (0.750–1.027)*	**56.8**
PB	5886/5628	**0.846 (0.758–0.945)**	**73.5**	**0.717 (0.577–0.891)**	**70.3**	**0.845 (0.735–0.972)**	**59.9**	**0.810 (0.698–0.940)**	**69.0**	**0.801 (0.674–0.951)**	**61.4**

^*^OR: Odds ratio; CI: confidence interval; PB: population-based; HWE: in all studies of this subgroup, the HWE's *P* value ≥ 0.05. GD: Graves’ disease; HT: Hashimoto's thyroiditis; MS: multiple sclerosis; SSc: systemic sclerosis; SLE: systemic lupus erythematosus; RA: rheumatoid arthritis; BD: Behçet's disease.

^*^Results with statistical significant difference were marked as bold. Unstable results in sensitivity analyses were marked as italic.

### Sensitivity analyses

Sensitivity analyses were performed in any comparison and any subgroup including more than two studies. When study Tomer Y [23], Heward JM [25], Kurylowicz A [28] or Jacobson E [32] was excluded, statistically different results were obtained in allelic comparison (T vs C) of GD's Caucasian subgroup. Statistically different results were obtained in recessive model (TT vs CC+CT) of MS and in several genetic models of MS's HWE and PB subgroup. Statistically different results were obtained in heterozygote comparison (CT vs CC) of SLE (systemic lupus erythematosus). Overall, statistically different results were obtained in several genetic models and in some subgroups. (Table 1 and Supplementary Data).

Because of the limited number of included studies, sensitivity analyses could not be performed in RA (rheumatoid arthritis), BD (Behçet's disease) and PB subgroup of SLE.

Other results showed stability in sensitivity analyses. (Supplementary data).

### Publication bias

Begg's funnel plot and Egger's test were used to assess the publication bias. Symmetry of funnel plot, *P* value of Begg's test (P_B_) and *P* value of Egger's test (P_E_) were evaluated in every genetic model overall and in MS, GD, GD's Asian subgroup, GD's Caucasian subgroup. Significant publication bias was found in heterozygote comparison (CT vs CC) overall (P_B_ = 0.043), dominant model (CT+TT vs CC) overall (P_B_ = 0.000), recessive model (TT vs CC+CT) overall (P_E_ = 0.021), recessive model (TT vs CC+CT) of MS (P_E_ = 0.033), homozygote comparison (TT vs CC) of GD's Asian subgroup (P_E_ = 0.016), dominant model (CT+TT vs CC) of GD's Caucasian subgroup (P_E_ = 0.044). (Supplementary data).

## DISCUSSION

In GD, 5006 cases/4537 controls were involved, and we found CD40 -1C>T was associated with decreased risk of GD in any genetic model and the results showed stability in sensitivity analyses and no publication bias. Similar stable results were shown in GD's Asian subgroup in any genetic model, but significant publication bias was found in homozygote comparison (TT vs CC). Similar stable results were shown in GD's Caucasian subgroup in heterozygote comparison (CT vs CC) and dominant model (CT+TT vs CC), but significant publication bias was found in dominant model (CT+TT vs CC). This may reveal the difference in ethnicity.

In MS, 3851 cases/4368 controls were involved, and we found CD40 -1C>T was associated with increased risk of MS in 4 genetic models and the results showed stability and no publication bias. Similar stable results were found in its HWE and PB subgroups in allelic comparison (T vs C) and dominant model (CT+TT vs CC), however, publication bias analyses could not be performed. Our results in MS was consistent with the GWAS (OR: 1.0740; 95%CI: 1.0360–1.1140; *P* value: 1.19E-04) performed by IMSGC et al. [65] in Caucasian.

In HT (576 cases/604 controls), SSc (2605 cases/3138 controls) and Asthma (675 cases/333 controls), no association was found in any genetic model and the results showed stability in sensitivity analyses.

In SLE (905 cases/1307 controls), no evidence was found for the association between CD40 -1C>T and SLE susceptibility in any genetic model, however, the results in heterozygote comparison (CT vs CC) lacked stability. In three studies of SLE, increased risk of SLE was found in dominant model (CT+TT vs CC: OR (95%CI) = 1.27 (1.02, 1.57), *P* = 0.03) of Joo YB [48] (593 cases/978 controls; Asian), however it lost significance after correction by age and sex. Similar results were found in dominant model (CT+TT vs CC: OR (95%CI) = 1.88 (1.23, 2.89), *P* = 0.004) of Wu C [50] (205 cases/220 controls; Asian), but no correction was done further. In Zhu Q [49] (107 cases/109 controls; Asian), decreased risk of SLE was found in dominant model (CT+TT vs CC: OR (95%CI) = 0.495 (0.278, 0.881), *P* = 0.017) and no correction was done. Thus no certain conclusion can be drawn about the association between CD40 -1C>T and SLE susceptibility, and more studies were needed.

In RA, BD, MG, CD, UC, Sarcoidosis, FUS, VKH, KD, GCA and ITP, less than three studies were included so that sensitivity analyses could not be performed. In two studies of RA, decreased risk of RA was found in allelic comparison (T vs C: OR (95%CI) = 0.89 (0.79, 0.99), *P* = 0.038) and homozygote comparison (TT vs CC: OR (95%CI) = 0.735 (0.553, 0.978), *P* = 0.035) of García BM [55] (1510 cases/1545 controls; Caucasian), but no association was found in any genetic model of Liu R [54] (212 cases/476 controls; Asian). In two studies of BD, increased risk of BD was found in recessive model (TT vs TC+CC: OR (95%CI) = 1.73 (1.22, 2.46), *P* = 0.002) and homozygote comparison (TT vs CC: OR (95%CI) = 1.565 (1.057, 2.317), *P* = 0.025) of Chen F [56] (373 cases/402 controls; Asian), but no association was found in any genetic model of İnal EE [57] (285 cases/225 controls; Caucasian).

Overall, because the association between CD40 -1C>T and GD conflicted with MS, the importance and scientific significance of the results decreased. Furthermore, 15 results were found unstable in sensitivity analyses and significant publication bias was found in 3 genetic models.

CD40 is expressed by some immune and non-immune cells. Thyroid follicular cells and orbital fibroblasts also express CD40 [66, 67]. Orbital fibroblasts are the target cells in Graves ophthalmopathy. *In vitro* studies demonstrated that the activation of CD40 on orbital fibroblasts leads to increased glycosaminoglycan productions, suggesting an important role in the pathogenesis of Graves’ ophthalmopathy [68]. The association between CD40 overactivation and GD has been firmly established. Transgenic mouse models constitutively overexpressing thyroidal CD40 develop more severe experimental autoimmune GD and thyrotoxicosis, whereas blockade of CD40 stimulation in experimental animal models suppresses progression to overt thyroiditis [69]. Similarly, functional blockade of CD40 with a murine antibody effectively prevents clinical expression in an animal model of multiple sclerosis. [70] CD40 -1C>T decreased the translational efficiency of CD40 transcripts, resulting in less CD40 protein level. However, our meta-analysis result and GWAS by IMSGC et al. [65] indicated that CD40 -1C>T is associated with increased risk of MS. Recently research showed that CD4(+) and CD8(+) Treg, which can be induced by CD40-activated B cells, play important roles in the maintenance of immune tolerance. The immune function of CD4(+)CD25(high) Tregs in MS patients significantly decreases as compared with normal controls. Adoptive transfer of CD8(+) Treg in rodents or induction of CD8(+) Treg in humans can prevent or treat autoimmune diseases. [71–73] It seems that different immune-related disease involves in different kind of disruption of immune balance.

Meanwhile, the limitations of this meta-analysis need to be addressed. To date, the number of available studies which can be included in this meta-analysis were small, especially available studies about HT, MS, SSc, SLE, Asthma, RA, BD, MG, CD, UC, Sarcoidosis, FUS, VKH, KD, GCA and ITP. Data for subgroup analyses were scanty. For example, gender subgroup analyses can not be done. For another example, the ethnicity in all studies of HT, SLE and Asthma were Asian, and in all studies of MS and SSc were Caucasian. Sensitivity analyses and publication bias analyses could not be performed in all subgroups. Some studies shared there controls with each other, like study NO.2 shared controls with NO.20, which were counted redundantly. (Detailed in Supplementary Table 1) Related studies published in other languages or unpublished were possibly missed.

In conclusion, our results suggested that: (1) CD40 -1C>T (rs1883832) is associated with decreased risk of Graves’ disease (GD), especially in Asian; (2) CD40 -1C>T (rs1883832) is associated with increased risk of MS (multiple sclerosis); (3) CD40 -1C>T (rs1883832) is not associated with the susceptibility of Hashimoto's thyroiditis (HT), systemic sclerosis (SSc) or Asthma; (4) there is insufficient data to fully confirm the association between CD40 -1C>T (rs1883832) and SLE, RA, BD, MG, CD, UC, Sarcoidosis, FUS, VKH, KD, GCA or ITP, and the results should be interpreted with caution. Well-designed studies with larger sample size and more subgroups are required to validate the risk identified in the current meta-analysis.

## SUPPLEMENTARY MATERIALS FIGURES AND TABLES







## References

[1] Munroe ME (2009). Functional roles for T cell CD40 in infection and autoimmune disease: the role of CD40 in lymphocyte homeostasis. Semin Immunol.

[2] Toubi E, Shoenfeld Y (2004). The role of CD40-CD154 interactions in autoimmunity and the benefit of disrupting this pathway. Autoimmunity.

[3] Bishop GA, Hostager BS (2003). The CD40-CD154 interaction in B cell-T cell liaisons. Cytokine Growth Factor Rev.

[4] Peters AL, Stunz LL, Bishop GA (2009). CD40 and autoimmunity: the dark side of a great activator. Semin Immunol.

[5] Jacobson EM, Concepcion E, Oashi T, Tomer Y (2005). A Graves' disease-associated Kozak sequence single-nucleotide polymorphism enhances the efficiency of CD40 gene translation: a case for translational pathophysiology. Endocrinology.

[6] Li M, Sun H, Liu S, Yu J, Li Q, Liu P, Shen H, Sun D (2012). CD40 C/T-1 polymorphism plays different roles in Graves' disease and Hashimoto's thyroiditis: a meta-analysis. Endocr J.

[7] Hu Z, Chen X, Li W, Wu G (2015). The association between the polymorphisms in the CD40 gene and Graves’ disease-a Meta analysis. Chongqing Medical Journal.

[8] Wells GA, Shea B, O'Connell D, Peterson J, Welch V, Losos M, Tugwell P The Newcastle-Ottawa Scale (NOS) for assessing the quality of nonrandomised studies in meta-analyses. http://www.ohri.ca/programs/clinical_epidemiology/nosgen.pdf.

[9] Moher D, Liberati A, Tetzlaff J, Altman DG, PRISMA Group (2009). Preferred reporting items for systematic reviews and meta-analyses: the PRISMA statement. J Clin Epidemiol.

[10] Higgins J, Thompson SG (2002). Quantifying heterogeneity in a meta-analysis. Stat Med.

[11] DerSimonian R, Laird N (1986). Meta-analysis in clinical trials. Control Clin Trials.

[12] Begg CB, Mazumdar M (1994). Operating characteristics of a rank correlation test for publication bias. Biometrics.

[13] Egger M, Smith GD, Schneider M, Minder C (1997). Bias in meta-analysis detected by a simple, graphical test. BMJ.

[14] Jurecka-Lubieniecka B, Ploski R, Kula D, Krol A, Bednarczuk T, Kolosza Z, Tukiendorf A, Szpak-Ulczok S, Stanjek-Cichoracka A, Polanska J, Jarzab B (2013). Association between age at diagnosis of Graves' disease and variants in genes involved in immune response. PLoS One.

[15] Kim I, Cheong HS, Shin HD, Kim K, Kang C, Bae SC (2010). Associations of genetic polymorphisms in CD40 with susceptibility to SLE in Korean populations. Lupus.

[16] Korobko DS, Malkova NA, Bulatova EV, Babenko LA, Sazonov DV, Sokolova EA, Filipenko ML (2013). The effect of genetic factors on the phenotypic expression of multiple sclerosis. Zh Nevrol Psikhiatr Im S S Korsakova.

[17] Lee SH, Lee EB, Shin ES, Lee JE, Cho SH, Min KU, Park HW (2014). The Interaction Between Allelic Variants of CD86 and CD40LG: A Common Risk Factor of Allergic Asthma and Rheumatoid Arthritis. Allergy Asthma Immunol Res.

[18] Li Y, Tian CX, Wang M, Xia ZE (2007). Correlation of CD40 gene polymorphisms with acute coronary syndrome, hypertension and diabetes. Zhonghua Yi Xue Za Zhi.

[19] Tomer Y, Davies TF, Greenberg DA (2005). What is the contribution of a Kozak SNP in the CD40 gene to Graves' disease?. Clin Endocrinol (Oxf).

[20] Simmonds MJ, Heward JM, Franklyn JA, Gough SC (2005). The CD40 Kozak SNP: a new susceptibility loci for Graves' disease?. Clin Endocrinol (Oxf).

[21] Chen XM, Hu ZQ, Li W, Liu ML, Wu MF, Fang S, Wu G (2015). Relationship between CD40-1C/T polymorphism (rs1883832) and Graves’ disease of Han population in western region of Guangdong province. Int J Endocrinol Metab.

[22] Zhang Y, Zhu BZ, Sun Y (2008). Association of CD40 gene 5′ UTR C(-1)T, -58038T and C64610G polymorphism with autoimmune thyroid disorders. Journal of Xi’an Jiaotong University (Medical Sciences).

[23] Tomer Y, Concepcion E, Greenberg DA (2002). A C/T single-nucleotide polymorphism in the region of the CD40 gene is associated with Graves' disease. Thyroid.

[24] Kim TY, Park YJ, Hwang JK, Song JY, Park KS, Cho BY, Park DJ (2003). A C/T polymorphism in the 5'-untranslated region of the CD40 gene is associated with Graves' disease in Koreans. Thyroid.

[25] Heward JM, Simmonds MJ, Carr-Smith J, Foxall H, Franklyn JA, Gough SC (2004). A single nucleotide polymorphism in the CD40 gene on chromosome 20q (GD-2) provides no evidence for susceptibility to Graves' disease in UK Caucasians. Clin Endocrinol (Oxf).

[26] Houston FA, Wilson V, Jennings CE, Owen CJ, Donaldson P, Perros P, Pearce SH (2004). Role of the CD40 locus in Graves' disease. Thyroid.

[27] Mukai T, Hiromatsu Y, Fukutani T, Ichimura M, Kaku H, Miyake I, Yamada K (2005). A C/T polymorphism in the 5' untranslated region of the CD40 gene is associated with later onset of Graves' disease in Japanese. Endocr J.

[28] Kurylowicz A, Kula D, Ploski R, Skorka A, Jurecka-Lubieniecka B, Zebracka J, Steinhof-Radwanska K, Hasse-Lazar K, Hiromatsu Y, Jarzab B, Bednarczuk T (2005). Association of CD40 gene polymorphism (C-1T) with susceptibility and phenotype of Graves' disease. Thyroid.

[29] Luo HX, Zhang P, Gao ZH, Qiu MC (2006). Association between Hepatic Injury of Graves’ Disease and a C/T Single-Nucleotide Polymorphism of the CD40 Gene. Tianjin Med J.

[30] Meng FT (2006). Study on the association of a C/T SNP in the Kozak of the CD40 gene with Graves Disease. Harbin Medical University.

[31] Ban Y, Tozaki T, Taniyama M, Tomita M, Ban Y (2006). Association of a C/T single-nucleotide polymorphism in the 5' untranslated region of the CD40 gene with Graves' disease in Japanese. Thyroid.

[32] Jacobson EM, Huber AK, Akeno N, Sivak M, Li CW, Concepcion E, Ho K, Tomer Y (2007). A CD40 Kozak sequence polymorphism and susceptibility to antibody-mediated autoimmune conditions: the role of CD40 tissue-specific expression. Genes Immun.

[33] Sun LL, Chu X, Huang W, Bi FY (2007). Correlation between CD40 gene polymorphism and Graves disease. The Journal of Practical Medicine.

[34] Makni K, Hadj Kacem H, Rebaï A, Abid M, Ayadi H (2007). Association and linkage studies of the 20q11.2 region (GRD-2 locus) with Graves' disease in the Tunisian population. Ann Hum Biol.

[35] Hsiao JY, Tien KJ, Hsiao CT, Hsieh MC (2008). A C/T polymorphism in CD40 gene is not associated with susceptibility and phenotype of Graves' disease in Taiwanese. Endocr J.

[36] Su YM, Xiao ZH, Huang P, Fu MJ, Zhou Z (2009). Correlation between CD40 gene polymorphism and Graves' disease in Guangdong Han population of south China. Guangdong Medical Journal.

[37] Ma LD, Yan SL, Li P, Wang L, Qi YQ (2010). The relationship between CD40 gene 5′ untranslated region position _1 site C/T polymorphism and the relapse of Grave's disease after antithyroid withdrawal. Immunological Journal.

[38] Yang J, Qin Q, Yan N, Zhu YF, Li C, Yang XJ, Wang X, Pandey M, Hou P, Zhang JA (2012). CD40 C/T(-1) and CTLA-4 A/G SNPs are associated with autoimmune thyroid diseases in the Chinese population. Endocrine.

[39] Inoue N, Watanabe M, Yamada H, Takemura K, Hayashi F, Yamakawa N, Akahane M, Shimizuishi Y, Hidaka Y, Iwatani Y (2012). Associations between autoimmune thyroid disease prognosis and functional polymorphisms of susceptibility genes, CTLA4, PTPN22, CD40, FCRL3, and ZFAT, previously revealed in genome-wide association studies. J Clin Immunol.

[40] Huang JZ, Li J, Dai CP (2013). Correlation between CD40 gene polymorphism and Graves disease. Int J Lab Med.

[41] Chen X, Hu Z, Li W, Wu P, Liu M, Bao L, Wu M, Fang S, Xiong W, Chen M, Wu G (2015). Synergistic combined effect between CD40-1C>T and CTLA-4+6230G>A polymorphisms in Graves' disease. Gene.

[42] Buck D, Kroner A, Rieckmann P, Mäurer M, Wiendl H (2006). Analysis of the C/T(-1) single nucleotide polymorphism in the CD40 gene in multiple sclerosis. Tissue Antigens.

[43] Blanco-Kelly F, Matesanz F, Alcina A, Teruel M, Díaz-Gallo LM, Gómez-García M, López-Nevot MA, Rodrigo L, Nieto A, Cardeña C, Alcain G, Díaz-Rubio M, de la Concha EG (2010). CD40: novel association with Crohn's disease and replication in multiple sclerosis susceptibility. PLoS One.

[44] Sokolova EA, Malkova NA, Korobko DS, Rozhdestvenskii AS, Kakulya AV, Khanokh EV, Delov RA, Platonov FA, Popova TY, Aref' eva EG, Zagorskaya NN, Alifirova VM, Titova MA (2013). Association of SNPs of CD40 gene with multiple sclerosis in Russians. PLoS One.

[45] Wagner M, Wisniewski A, Bilinska M, Pokryszko-Dragan A, Cyrul M, Kusnierczyk P, Jasek M (2014). Investigation of gene-gene interactions between CD40 and CD40L in Polish multiple sclerosis patients. Hum Immunol.

[46] Field J, Shahijanian F, Johnson L, Gresle M, Laverick L, Parnell G, Stewart G, McKay F, Kilpatrick T, Butzkueven H, Booth D, Australia Schibeci S; New Zealand MS Genetics Consortium ANZgene (2015). The MS Risk Allele of CD40 Is Associated with Reduced Cell-Membrane Bound Expression in Antigen Presenting Cells: Implications for Gene Function. PLoS One.

[47] Teruel M, Simeon CP, Broen J, Vonk MC, Carreira P, Camps MT, García-Portales R, Delgado-Frías E, Gallego M, Espinosa G, Beretta L, Airó P, Spanish Scleroderma Group (2012). Analysis of the association between CD40 and CD40 ligand polymorphisms and systemic sclerosis. Arthritis Res Ther.

[48] Joo YB, Park BL, Shin HD, Park SY, Kim I, Bae SC (2013). Association of genetic polymorphisms in CD40 with susceptibility to SLE in the Korean population. Rheumatology (Oxford).

[49] Zhu Q, Ding Q, Yu Q, Li W, Xu H (2015). Association of CD40 Gene Polymorphism with the Systemic Lupus Erythematosus. J Mod Lab Med.

[50] Wu C, Guo J, Luo H, Wei C, Wang C, Lan Y, Wei YS (2016). Association of CD40 polymorphisms and haplotype with risk of systemic lupus erythematosus. Rheumatol Int.

[51] Park JH, Chang HS, Park CS, Jang AS, Park BL, Rhim TY, Uh ST, Kim YH, Chung IY, Shin HD (2007). Association analysis of CD40 polymorphisms with asthma and the level of serum total IgE. Am J Respir Crit Care Med.

[52] Hsieh YY, Wan L, Chang CC, Tsai CH, Tsai FJ (2009). STAT2*C related genotypes and allele but not TLR4 and CD40 gene polymorphisms are associated with higher susceptibility for asthma. Int J Biol Sci.

[53] Du J, Xu H, Jie Q, Zhong Q, Wu Y, Xu J (2013). Analysis on the correlation of CD40-1C/T gene polymorphism with children asthma. Laboratory Medicine.

[54] Liu R, Xu N, Wang X, Shen L, Zhao G, Zhang H, Fan W (2012). Influence of MIF, CD40, and CD226 polymorphisms on risk of rheumatoid arthritis. Mol Biol Rep.

[55] García-Bermúdez M, González-Juanatey C, López-Mejías R, Teruel M, Corrales A, Miranda-Filloy JA, Castañeda S, Balsa A, Fernández-Gutierrez B, González-Álvaro I, Gómez-Vaquero C, Blanco R, Llorca J (2012). Study of association of CD40-CD154 gene polymorphisms with disease susceptibility and cardiovascular risk in Spanish rheumatoid arthritis patients. PLoS One.

[56] Chen F, Hou S, Jiang Z, Chen Y, Kijlstra A, Rosenbaum JT, Yang P (2012). CD40 gene polymorphisms confer risk of Behcet's disease but not of Vogt-Koyanagi-Harada syndrome in a Han Chinese population. Rheumatology (Oxford).

[57] İnal EE, Rüstemoğlu A, İnanir A, Ekinci D, Gül Ü, Yiğit S, Ateş Ö (2015). Associations of rs4810485 and rs1883832 polymorphisms of CD40 gene with susceptibility and clinical findings of Behçet's disease. Rheumatol Int.

[58] Tanizawa K, Handa T, Nagai S, Ito I, Kubo T, Ito Y, Watanabe K, Aihara K, Mishima M, Izumi T (2011). A CD40 single-nucleotide polymorphism affects the lymphocyte profiles in the bronchoalveolar lavage of Japanese patients with sarcoidosis. Tissue Antigens.

[59] Chen F, Hou S, Jiang Z, Li F, Chen Y, Kijlstra A, Yang P (2011). CD40 polymorphisms in Han Chinese patients with Fuch uveitis syndrome. Mol Vis.

[60] Pu T, Xie J, Yang J, Chen S, Shen J (2015). Analysis of single nucleotide polymorphisms of CD40 gene of Han people with Kawasaki disease in Shanghai. Journal of Shanghai Jiaotong University (Medical Science).

[61] Rodríguez-Rodríguez L, Castañeda S, Vázquez-Rodríguez TR, Morado IC, Marí-Alfonso B, Gómez-Vaquero C, Miranda-Filloy JA, Narvaez J, Ortego-Centeno N, Blanco R, Fernández-Gutiérrez B, Martín J, González-Gay MA (2010). Influence of CD40 rs1883832 polymorphism in susceptibility to and clinical manifestations of biopsy-proven giant cell arteritis. J Rheumatol.

[62] Wei Y, He J, He H, Qu H, Yan H (2016). Correlation betweenCD40 Gene Polymorphismand Immune Thrombocytopenia. Journal of China Medical University.

[63] Chen J, Guo J, Wei C, Wang C, Luo H, Wei Y, Lan Y (2015). The association of CD40 polymorphisms with CD40 serum levels and risk of systemic lupus erythematosus. BMC Genet.

[64] Kudryavtseva E, Aulchenko Y, Filipenko M (2013). Role SNPs of CD40 in predisposition to multiple sclerosis. FEBS Journal.

[65] Beecham AH, Patsopoulos NA, Xifara DK, Davis MF, Kemppinen A, Cotsapas C, Shah TS, Spencer C, Booth D, Goris A, Oturai A, Saarela J, Fontaine B, International Multiple Sclerosis Genetics Consortium (IMSGC) (2013). Analysis of immune-related loci identifies 48 new susceptibility variants for multiple sclerosis. Nat Genet.

[66] Smith TJ, Sempowski GD, Berenson CS, Cao HJ, Wang HS, Phipps RP (1997). Human thyroid fibroblasts exhibit a distinctive phenotype in culture: characteristic ganglioside profile and functional CD40 expression. Endocrinology.

[67] Metcalfe RA, McIntosh RS, Marelli-Berg F, Lombardi G, Lechler R, Weetman AP (1998). Detection of CD40 on human thyroid follicular cells: analysis of expression and function. J Clin Endocrinol Metab.

[68] Cao HJ, Wang HS, Zhang Y, Lin HY, Phipps RP, Smith TJ (1998). Activation of human orbital fibroblasts through CD40 engagement results in a dramatic induction of hyaluronan synthesis and prostaglandin endoperoxide H synthase-2 expression. Insights into potential pathogenic mechanisms of thyroid-associated ophthalmopathy. J Biol Chem.

[69] Lee HJ, Lombardi A, Stefan M, Li CW, Inabnet WB, Owen RP, Concepcion E, Tomer Y (2017). CD40 Signaling in Graves Disease Is Mediated Through Canonical and Noncanonical Thyroidal Nuclear Factor κB Activation. Endocrinology.

[70] Boon L, Brok HP, Bauer J, Ortiz-Buijsse A, Schellekens MM, Ramdien-Murli S, Blezer E, van Meurs M, Ceuppens J, de Boer M, 't Hart BA, Laman JD (2001). Prevention of experimental autoimmune encephalomyelitis in the common marmoset (Callithrix jacchus) using a chimeric antagonist monoclonal antibody against human CD40 is associated with altered B cell responses. J Immunol.

[71] Zheng J, Liu Y, Qin G, Chan PL, Mao H, Lam KT, Lewis DB, Lau YL, Tu W (2009). Efficient induction and expansion of human alloantigen-specific CD8 regulatory T cells from naive precursors by CD40-activated B cells. J Immunol.

[72] Xiang YJ, Ren M, Jiang H, Yang TT, He Y, Ao DH, Wang YY, Zhang Q, He XJ, Gao XG, Liu GZ (2016). Ex vivo expansion of antigen-specific CD4+CD25+ regulatory T cells from autologous naïve CD4+ T cells of multiple sclerosis patients as a potential therapeutic approach. Eur Rev Med Pharmacol Sci.

[73] Haas J, Hug A, Viehöver A, Fritzsching B, Falk CS, Filser A, Vetter T, Milkova L, Korporal M, Fritz B, Storch-Hagenlocher B, Krammer PH, Suri-Payer E (2005). Reduced suppressive effect of CD4+CD25high regulatory T cells on the T cell immune response against myelin oligodendrocyte glycoprotein in patients with multiple sclerosis. Eur J Immunol.

