# Supplementary material for: Association between CD40 rs1883832 and immune-related diseases susceptibility: A meta-analysis
Source: Oncotarget. 2017 Jun 28;8(60):102235–43. doi: 10.18632/oncotarget.18704 (PMC5731949; doi:10.18632/oncotarget.18704)
Supplement: Supplementary file 1 [file oncotarget-08-102235-s001.pdf]

Association between CD40 rs1883832 and immune-related diseases susceptibility: a meta-analysis

Supplementary Materials

Supplementary Table 1: Characteristics of studies included in the meta-analysis. See Supplementary_Table_1

Supplementary Data: Sensitivity analyses results and publication bias results. See Supplementary_Data
