# Supplementary material for: Association between CD40 rs1883832 and immune-related diseases susceptibility: A meta-analysis
Source: Oncotarget. 2017 Jun 28;8(60):102235–43. doi: 10.18632/oncotarget.18704 (PMC5731949; doi:10.18632/oncotarget.18704)
Supplement: Supplementary file 2 [file oncotarget-08-102235-s002.doc]

**Supplementary Table 1.** Characteristics of studies included in the meta-analysis.

| NO. | Study ID | Year | Country or Area | Ethnicity | Control Type | Genotyping  Method | Case | | | Control | | | P for HWE* | Quality |
| --- | --- | --- | --- | --- | --- | --- | --- | --- | --- | --- | --- | --- | --- | --- |
|  | **CD40** **rs1883832** |  |  |  |  |  | CC | CT | TT | CC | CT | TT |  |  |
|  | **GD*** |  |  |  |  |  |  |  |  |  |  |  |  |  |
| 1 | **Tomer Y [23]** | 2002 | North America, Italy,  UK and Israel | Caucasian | PB* | PCR-RFLP | 100 | 53 | 1 | 64 | 49 | 5 | 0.244 | 7 |
| 2 | **Kim TY [24]** | 2003 | Korea | Asian | PB | PCR-RFLP | 66 | 56 | 10 | *56** | *79* | *29* | 0.901 | 7 |
| 3 | **Heward JM [25]** | 2004 | UK | Caucasian | PB | PCR-RFLP | 481 | 276 | 43 | 434 | 306 | 45 | 0.350 | 8 |
| 4 | **Houston F [26]** | 2004 | UK | Caucasian | PB | PCR-RFLP | 250 | 175 | 26 | 244 | 182 | 20 | 0.053 | 7 |
| 5 | **Mukai T [27]** | 2005 | Japan | Asian | PB | PCR-RFLP | 121 | 152 | 51 | 80 | 108 | 41 | 0.665 | 7 |
| 6 | **Kurylowicz A[28]** | 2005 | Poland | Caucasian | PB | PCR-RFLP | 336 | 192 | 28 | 341 | 237 | 33 | 0.322 | 7 |
| 7 | **Luo H [29]** | 2006 | China | Asian | PB | PCR-RFLP | 60 | 35 | 25 | 34 | 39 | 33 | **0.007*** | 9 |
| 8 | **Meng F [30]** | 2006 | China | Asian | PB | PCR-RFLP | 70 | 92 | 37 | 39 | 97 | 40 | 0.175 | 8 |
| 9 | **Ban Y [31]** | 2006 | Japan | Asian | PB | PCR-RFLP | 114 | 156 | 31 | *67* | *78* | *32* | 0.271 | 7 |
| 10 | **Jacobson E [32]** | 2007 | USA | Caucasian | PB | PCR-RFLP | 134 | 69 | 7 | *135* | *112* | *24* | 0.911 | 7 |
| 11 | **Sun L [33]** | 2007 | China | Asian | PB | Sequencing | 141 | 172 | 54 | 127 | 174 | 72 | 0.371 | 8 |
| 12 | **Makni K [34]** | 2007 | Tunisia | Caucasian | PB | PCR-RFLP | 67 | 8 | 1 | 62 | 4 | 0 | 0.800 | 7 |
| 13 | **Hsiao JY [35]** | 2008 | Taiwan | Asian | PB | PCR-RFLP | 74 | 116 | 25 | 49 | 77 | 15 | 0.058 | 7 |
| 14 | **Su Y [36]** | 2009 | China | Asian | PB | PCR-RFLP | 57 | 43 | 19 | 25 | 48 | 30 | 0.505 | 9 |
| 15 | **Ma L [37]** | 2010 | China | Asian | PB | PCR-RFLP | 47 | 136 | 15 | 13 | 68 | 29 | **0.006** | 8 |
| 16 | **Yang J [38]** | 2012 | China | Asian | PB | PCR-RFLP | 147 | 141 | 15 | *87* | *108* | *20* | 0.098 | 8 |
| 17 | **Inoue N [39]** | 2012 | Japan | Asian | PB | PCR-RFLP | 35 | 45 | 13 | *14* | *21* | *13* | 0.388 | 7 |
| 18 | **Huang J [40]** | 2013 | China | Asian | PB | PCR-RFLP | 59 | 37 | 32 | 43 | 55 | 52 | **0.001** | 8 |
| 19 | **Chen X [41]** | 2015 | China | Asian | HB* | PCR-HRM | 84 | 118 | 58 | 56 | 108 | 84 | 0.064 | 6 |
|  | **HT*** |  |  |  |  |  |  |  |  |  |  |  |  |  |
| 20 | **Kim TY [24]** | 2003 | Korea | Asian | PB | PCR-RFLP | 38 | 58 | 22 | *56* | *79* | *29* | 0.901 | 7 |
| 21 | **Ban Y [31]** | 2006 | Japan | Asian | PB | PCR-RFLP | 62 | 93 | 29 | *67* | *78* | *32* | 0.271 | 7 |
| 22 | **Yang J [38]** | 2012 | China | Asian | PB | PCR-RFLP | 81 | 99 | 28 | *87* | *108* | *20* | 0.098 | 7 |
| 23 | **Inoue N [39]** | 2012 | Japan | Asian | PB | PCR-RFLP | 16 | 33 | 17 | *14* | *21* | *13* | 0.388 | 7 |
|  | **MS*** |  |  |  |  |  |  |  |  |  |  |  |  |  |
| 24 | **Buck D [42]** | 2006 | Germany | Caucasian | PB | PCR-RFLP | 136 | 115 | 36 | 94 | 74 | 16 | 0.792 | 8 |
| 25 | **Blanco KF [43]** | 2010 | Spain | Caucasian | PB | TaqMan PCR | 768 | 625 | 137 | *1562* | *1098* | *237* | **0.026** | 7 |
| 26 | **Sokolova EA[44]** | 2013 | Russia | Caucasian | PB+HB | TaqMan PCR | 927 | 634 | 118 | 532 | 299 | 48 | 0.483 | 6 |
| 27 | **Wagner M [45]** | 2014 | Poland | Caucasian | PB | PCR-RFLP | 166 | 137 | 31 | 194 | 114 | 14 | 0.592 | 7 |
| 28 | **Field J [46]** | 2015 | Australia and New Zealand | Caucasian | PB | TaqMan PCR and PCR-RFLP | 12 | 7 | 2 | 49 | 27 | 10 | 0.052 | 6 |
|  |  |  |  |  |  |  |  |  |  |  |  |  |  |  |
| **Supplementary Table 1.** Continued. |  |  |  |  |  |  |  |  |  |  |  |  |  |  |
|  |  |  |  |  |  |  |  |  |  |  |  |  |  |  |
|  | **SSc*** |  |  |  |  |  |  |  |  |  |  |  |  |  |
| 29 | **Teruel M [47]** | 2012 | Europe | Caucasian | PB | TaqMan PCR |  |  |  |  |  |  |  |  |
| 29.1 |  |  | Spain |  |  |  | 579 | 414 | 90 | 815 | 607 | 124 | 0.462 | 7 |
| 29.2 |  |  | Germany |  |  |  | 289 | 190 | 48 | 224 | 174 | 21 | 0.081 | 7 |
| 29.3 |  |  | Netherland |  |  |  | 216 | 142 | 16 | 297 | 156 | 31 | 0.093 | 7 |
| 29.4 |  |  | Italy |  |  |  | 309 | 254 | 58 | 350 | 279 | 60 | 0.679 | 7 |
|  | **SLE*** |  |  |  |  |  |  |  |  |  |  |  |  |  |
| 30 | **Joo YB [48]** | 2013 | Korea | Asian | PB | TaqMan PCR | 242 | 281 | 70 | 451 | 418 | 109 | 0.414 | 7 |
| 31 | **Zhu Q [49]** | 2015 | China | Asian | PB | PCR-RFLP | 44 | 42 | 21 | 28 | 60 | 21 | 0.271 | 8 |
| 32 | **Wu C [50]** | 2016 | China | Asian | HB | Sequencing | 47 | 105 | 53 | 79 | 101 | 40 | 0.440 | 7 |
|  | **Asthma** |  |  |  |  |  |  |  |  |  |  |  |  |  |
| 33 | **Park JH [51]** | 2007 | Korea | Asian | PB | Sequencing | 175 | 199 | 50 | 73 | 67 | 20 | 0.453 | 8 |
| 34 | **Hsieh YY [52]** | 2009 | China | Asian | PB | PCR-RFLP | 35 | 62 | 20 | 22 | 25 | 13 | 0.253 | 7 |
| 35 | **Du J [53]** | 2013 | China | Asian | PB | PCR-RFLP | 49 | 60 | 25 | 28 | 64 | 21 | 0.145 | 8 |
|  | **RA*** |  |  |  |  |  |  |  |  |  |  |  |  |  |
| 36 | **Liu R [54]** | 2012 | China | Asian | HB | MassARRAY | 77 | 105 | 30 | 182 | 234 | 60 | 0.254 | 7 |
| 37 | **García BM [55]** | 2012 | Spain | Caucasian | PB | TaqMan PCR | 839 | 577 | 94 | 814 | 607 | 124 | 0.468 | 7 |
|  | **BD*** |  |  |  |  |  |  |  |  |  |  |  |  |  |
| 38 | **Chen F [56]** | 2012 | China | Asian | PB | PCR-RFLP | 130 | 147 | 96 | *142* | *193* | *67* | 0.917 | 7 |
| 39 | **İnal EE [57]** | 2015 | Turkey | Caucasian | PB | PCR-RFLP | 132 | 126 | 27 | 97 | 104 | 24 | 0.618 | 7 |
|  | **MG*** |  |  |  |  |  |  |  |  |  |  |  |  |  |
| 40 | **Jacobson E [32]** | 2007 | USA | Caucasian | PB | PCR-RFLP | 42 | 32 | 7 | *135* | *112* | *24* | 0.911 | 6 |
|  | **CD*** |  |  |  |  |  |  |  |  |  |  |  |  |  |
| 41 | **Blanco KF [43]** | 2010 | Spain | Caucasian | PB | TaqMan PCR | 527 | 452 | 107 | *1562* | *1098* | *237* | **0.026** | 7 |
|  | **UC*** |  |  |  |  |  |  |  |  |  |  |  |  |  |
| 42 | **Blanco KF [43]** | 2010 | Spain | Caucasian | PB | TaqMan PCR | 485 | 398 | 66 | *1562* | *1098* | *237* | **0.026** | 7 |
|  | **Sarcoidosis** |  |  |  |  |  |  |  |  |  |  |  |  |  |
| 43 | **Tanizawa K [58]** | 2011 | Japan | Asian | PB | TaqMan PCR | 56 | 90 | 26 | 56 | 72 | 22 | 0.883 | 7 |
|  | **FUS*** |  |  |  |  |  |  |  |  |  |  |  |  |  |
| 44 | **Chen F [59]** | 2011 | China | Asian | PB | PCR-RFLP | 44 | 61 | 26 | *142* | *193* | *67* | 0.917 | 8 |
|  | **VKH*** |  |  |  |  |  |  |  |  |  |  |  |  |  |
| 45 | **Chen F [56]** | 2012 | China | Asian | PB | PCR-RFLP | 177 | 243 | 99 | *142* | *193* | *67* | 0.917 | 8 |
|  |  |  |  |  |  |  |  |  |  |  |  |  |  |  |
|  |  |  |  |  |  |  |  |  |  |  |  |  |  |  |
|  |  |  |  |  |  |  |  |  |  |  |  |  |  |  |
|  | **KD*** |  |  |  |  |  |  |  |  |  |  |  |  |  |
| 46 | **Pu T [60]** | 2015 | China | Asian | PB | Sequencing | 30 | 37 | 14 | 35 | 42 | 19 | 0.327 | 8 |
|  | **GCA*** |  |  |  |  |  |  |  |  |  |  |  |  |  |
| 47 | **Rodríguez [61]** | 2010 | Spain | Caucasian | PB | TaqMan PCR | 161 | 107 | 37 | 428 | 295 | 65 | 0.164 | 8 |
|  | **ITP*** |  |  |  |  |  |  |  |  |  |  |  |  |  |
| 48 | **Wei Y [62]** | 2016 | China | Asian | PB | PCR-RFLP | 77 | 57 | 26 | 39 | 74 | 47 | 0.358 | 7 |

* HWE: Hardy–Weinberg equilibrium; PB: population-based; HB: hospital-based; GD: Graves’ disease; HT: Hashimoto’s thyroiditis; MS: multiple sclerosis; SSc: systemic sclerosis;

SLE: systemic lupus erythematosus; RA: rheumatoid arthritis; BD: Behçet's disease; MG: myasthenia gravis; CD: Crohn’s disease; UC: ulcerative colitis; FUS: Fuch uveitis syndrome;

VKH: Vogt-Koyanagi-Harada syndrome; KD: Kawasaki disease; GCA: giant cell arteritis; ITP: Immune thrombocytopenia.

***** Results with statistical significant difference were marked as bold. Control group shared with other studies were marked as italics.

**Supplementary Table 1.** Continued.
