# Supplementary material for: Association between CD40 rs1883832 and immune-related diseases susceptibility: A meta-analysis
Source: Oncotarget. 2017 Jun 28;8(60):102235–43. doi: 10.18632/oncotarget.18704 (PMC5731949; doi:10.18632/oncotarget.18704)
Supplement: Supplementary file 3 [file oncotarget-08-102235-s003.docx]

**Supplementary data**

1：allelic comparison (T vs C)

2：homozygote comparison (TT vs CC)

3：heterozygote comparison (CT vs CC)

4：dominant model (CT+TT vs CC)

5：recessive model (TT vs CC+CT)

*（1）Sensitivity analyses results：*

**Asthma**

1

------------------------------------------------------------------------------

Study ommited | e^coef. [95% Conf. Interval]

-------------------+----------------------------------------------------------

Park JH [51] | .88044787 .66648229 1.1631044

Hsieh YY [52] | .96455992 .77703874 1.1973352

Du J [53] | 1.0735059 .85139762 1.3535567

-------------------+----------------------------------------------------------

Combined | .97952336 .80644883 1.189742

2

------------------------------------------------------------------------------

Study ommited | e^coef. [95% Conf. Interval]

-------------------+----------------------------------------------------------

Park JH [51] | .78774465 .44666218 1.3892863

Hsieh YY [52] | .88525999 .55875564 1.4025545

Du J [53] | 1.0188954 .62573184 1.6590937

-------------------+----------------------------------------------------------

Combined | .90225789 .60019738 1.356336

3

------------------------------------------------------------------------------

Study ommited | e^coef. [95% Conf. Interval]

-------------------+----------------------------------------------------------

Park JH [51] | .8961564 .31484797 2.5507431

Hsieh YY [52] | .83888374 .369599 1.9040255

Du J [53] | 1.3068453 .92949051 1.8373988

-------------------+----------------------------------------------------------

Combined | 1.0071452 .55550918 1.8259671

4

------------------------------------------------------------------------------

Study ommited | e^coef. [95% Conf. Interval]

-------------------+----------------------------------------------------------

Park JH [51] | .86379908 .37056248 2.0135575

Hsieh YY [52] | .85142244 .41458539 1.7485425

Du J [53] | 1.2305241 .8935666 1.6945458

-------------------+----------------------------------------------------------

Combined | .97785926 .59231795 1.6143504

5

------------------------------------------------------------------------------

Study ommited | e^coef. [95% Conf. Interval]

-------------------+----------------------------------------------------------

Park JH [51] | .89049723 .5421267 1.4627306

Hsieh YY [52] | .96459616 .63402893 1.4675131

Du J [53] | .86718789 .55206948 1.3621743

-------------------+----------------------------------------------------------

Combined | .91041263 .6291241 1.3174684

**SLE**

1

------------------------------------------------------------------------------

Study ommited | e^coef. [95% Conf. Interval]

-------------------+----------------------------------------------------------

Joo YB [48] | 1.0697308 .5259197 2.1758531

Zhu Q [49] | 1.2863127 .97750117 1.6926837

Wu C [50] | .95000364 .62026134 1.455043

-------------------+----------------------------------------------------------

Combined | 1.1145094 .80795646 1.5373739

2

------------------------------------------------------------------------------

Study ommited | e^coef. [95% Conf. Interval]

-------------------+----------------------------------------------------------

Joo YB [48] | 1.2270945 .36004141 4.1821883

Zhu Q [49] | 1.5707531 .85871488 2.8732067

Wu C [50] | .96261364 .53444094 1.7338212

-------------------+----------------------------------------------------------

Combined | 1.2493625 .69531673 2.2448856

3

------------------------------------------------------------------------------

Study ommited | e^coef. [95% Conf. Interval]

-------------------+----------------------------------------------------------

Joo YB [48] | .89709455 .23512566 3.4227597

**Zhu Q [49] | 1.3910691 1.0276232 1.8830572**

Wu C [50] | .77899812 .28371658 2.1388883

-------------------+----------------------------------------------------------

Combined | 1.04164 .57023931 1.9027341

------------------------------------------------------------------------------

4

------------------------------------------------------------------------------

Study ommited | e^coef. [95% Conf. Interval]

-------------------+----------------------------------------------------------

Joo YB [48] | .97982897 .26454543 3.6291112

Zhu Q [49] | 1.4639674 .98163076 2.1833062

Wu C [50] | .81668771 .3329039 2.0035175

-------------------+----------------------------------------------------------

Combined | 1.0932986 .61097784 1.9563752

5

------------------------------------------------------------------------------

Study ommited | e^coef. [95% Conf. Interval]

-------------------+----------------------------------------------------------

Joo YB [48] | 1.3676311 .93332979 2.0040235

Zhu Q [49] | 1.2078364 .9285638 1.5711023

Wu C [50] | 1.0588974 .79354358 1.4129831

-------------------+----------------------------------------------------------

Combined | 1.1816592 .92492578 1.5096545

**SSc**

1

------------------------------------------------------------------------------

Study ommited | e^coef. [95% Conf. Interval]

-------------------+----------------------------------------------------------

Teruel M [47] | 1.0508747 .93857103 1.176616

Teruel M [47] | 1.0123291 .92430023 1.1087416

Teruel M [47] | 1.0186766 .93152209 1.1139854

Teruel M [47] | 1.0157654 .92304125 1.1178041

-------------------+----------------------------------------------------------

Combined | 1.0218994 .94030714 1.1105717

2

------------------------------------------------------------------------------

Study ommited | e^coef. [95% Conf. Interval]

-------------------+----------------------------------------------------------

Teruel M [47] | 1.1436103 .8613741 1.5183234

Teruel M [47] | .99871594 .80209644 1.2435332

Teruel M [47] | 1.1374105 .91757799 1.4099102

Teruel M [47] | 1.0781537 .85009441 1.3673957

-------------------+----------------------------------------------------------

Combined | 1.0826373 .88353387 1.3266084

3

------------------------------------------------------------------------------

Study ommited | e^coef. [95% Conf. Interval]

-------------------+----------------------------------------------------------

Teruel M [47] | 1.0228696 .88121754 1.1872916

Teruel M [47] | 1.0266566 .90969586 1.1586552

Teruel M [47] | .95503695 .84742191 1.0763181

Teruel M [47] | .9829616 .86658882 1.1149619

-------------------+----------------------------------------------------------

Combined | .99398793 .89012593 1.1099688

4

------------------------------------------------------------------------------

Study ommited | e^coef. [95% Conf. Interval]

-------------------+----------------------------------------------------------

Teruel M [47] | 1.0418121 .90387113 1.2008044

Teruel M [47] | 1.0217693 .9108746 1.146165

Teruel M [47] | .98478792 .8791202 1.1031566

Teruel M [47] | .99870984 .88589392 1.1258925

-------------------+----------------------------------------------------------

Combined | 1.0087798 .90830733 1.120366

5

------------------------------------------------------------------------------

Study ommited | e^coef. [95% Conf. Interval]

-------------------+----------------------------------------------------------

Teruel M [47] | 1.1207479 .65725987 1.9110796

Teruel M [47] | .99446304 .80063314 1.2352183

Teruel M [47] | 1.2098901 .88290055 1.6579828

Teruel M [47] | 1.1026851 .66613121 1.8253377

-------------------+----------------------------------------------------------

Combined | 1.0985275 .79259422 1.522548

**HT**

1

------------------------------------------------------------------------------

Study ommited | e^coef. [95% Conf. Interval]

-------------------+----------------------------------------------------------

Kim TY [24] | 1.0851881 .89704654 1.3127895

Ban Y [31] | 1.0982373 .89914734 1.3414099

Yang J [38] | 1.0524942 .85696145 1.2926417

Inoue N [39] | 1.0799021 .90665401 1.2862554

-------------------+----------------------------------------------------------

Combined | 1.0793763 .91434353 1.2741963

2

------------------------------------------------------------------------------

Study ommited | e^coef. [95% Conf. Interval]

-------------------+----------------------------------------------------------

Kim TY [24] | 1.1888327 .79118438 1.7863387

Ban Y [31] | 1.2777908 .83222198 1.9619156

Yang J [38] | 1.0549636 .69528262 1.6007135

Inoue N [39] | 1.1736621 .80774831 1.7053366

-------------------+----------------------------------------------------------

Combined | 1.1701277 .82395913 1.6617316

3

------------------------------------------------------------------------------

Study ommited | e^coef. [95% Conf. Interval]

-------------------+----------------------------------------------------------

Kim TY [24] | 1.1334185 .8494236 1.5123638

Ban Y [31] | 1.0547852 .77771821 1.4305589

Yang J [38] | 1.21809 .88072093 1.6846917

Inoue N [39] | 1.1021073 .84605761 1.4356475

-------------------+----------------------------------------------------------

Combined | 1.1215633 .87023143 1.4454825

4

------------------------------------------------------------------------------

Study ommited | e^coef. [95% Conf. Interval]

-------------------+----------------------------------------------------------

Kim TY [24] | 1.1397959 .86725268 1.4979887

Ban Y [31] | 1.0985035 .82260369 1.4669396

Yang J [38] | 1.1690249 .86175519 1.5858554

Inoue N [39] | 1.1155195 .86821643 1.4332645

-------------------+----------------------------------------------------------

Combined | 1.1286416 .88768485 1.4350046

5

------------------------------------------------------------------------------

Study ommited | e^coef. [95% Conf. Interval]

-------------------+----------------------------------------------------------

Kim TY [24] | 1.0677337 .73931363 1.5420455

Ban Y [31] | 1.1940093 .81306426 1.7534384

Yang J [38] | .93848862 .64921283 1.3566597

Inoue N [39] | 1.0909499 .77657449 1.5325918

-------------------+----------------------------------------------------------

Combined | 1.0674893 .77887278 1.4630547

MS

1

------------------------------------------------------------------------------

Study ommited | e^coef. [95% Conf. Interval]

-------------------+----------------------------------------------------------

Buck D [42] | 1.1725794 1.0879417 1.2638016

Blanco KF [43] | 1.2497377 1.1206426 1.3937043

Sokolova EA[44] | 1.1612845 1.0659925 1.2650949

Wagner M [45] | 1.1477412 1.0640349 1.2380327

Field J [46] | 1.1762922 1.0937141 1.2651052

-------------------+----------------------------------------------------------

Combined | 1.1739711 1.091913 1.262196

2

------------------------------------------------------------------------------

Study ommited | e^coef. [95% Conf. Interval]

-------------------+----------------------------------------------------------

Buck D [42] | 1.3045296 1.0869744 1.5656279

Blanco KF [43] | 1.5706507 1.1915897 2.0702962

Sokolova EA[44] | 1.2933703 1.0562462 1.583728

Wagner M [45] | 1.2565981 1.047474 1.5074731

Field J [46] | 1.3289848 1.1138822 1.5856262

-------------------+----------------------------------------------------------

Combined | 1.3216277 1.1088345 1.5752576

3

------------------------------------------------------------------------------

Study ommited | e^coef. [95% Conf. Interval]

-------------------+----------------------------------------------------------

Buck D [42] | 1.1970837 1.0842147 1.3217026

Blanco KF [43] | 1.2274883 1.0655821 1.4139947

Sokolova EA[44] | 1.1774995 1.0494156 1.3212164

Wagner M [45] | 1.1704132 1.0584391 1.2942333

Field J [46] | 1.1905437 1.0810914 1.3110772

-------------------+----------------------------------------------------------

Combined | 1.1893611 1.0804593 1.3092393

4

------------------------------------------------------------------------------

Study ommited | e^coef. [95% Conf. Interval]

-------------------+----------------------------------------------------------

Buck D [42] | 1.2160952 1.106836 1.3361397

Blanco KF [43] | 1.2765604 1.1156735 1.4606482

Sokolova EA[44] | 1.1993571 1.075256 1.3377813

Wagner M [45] | 1.1857775 1.0777948 1.3045787

Field J [46] | 1.2147831 1.1083965 1.3313809

-------------------+----------------------------------------------------------

Combined | 1.2125937 1.1068542 1.3284346

5

------------------------------------------------------------------------------

Study ommited | e^coef. [95% Conf. Interval]

-------------------+----------------------------------------------------------

Buck D [42] | 1.2124333 1.0152695 1.4478861

Blanco KF [43] | 1.4550338 1.1106237 1.9062472

**Sokolova EA[44] | 1.2089278 .99359976 1.4709207**

**Wagner M [45] | 1.1788741 .98781566 1.4068862**

Field J [46] | 1.2386268 1.043303 1.4705184

-------------------+----------------------------------------------------------

Combined | 1.2324782 1.0391414 1.4617861

MS-HWE-sub

1

------------------------------------------------------------------------------

Study ommited | e^coef. [95% Conf. Interval]

-------------------+----------------------------------------------------------

Buck D [42] | 1.2594709 1.1192975 1.4171986

Sokolova EA[44] | 1.3311474 1.1092598 1.5974198

Wagner M [45] | 1.1969803 1.060321 1.3512529

Field J [46] | 1.2570319 1.1259173 1.4034151

-------------------+----------------------------------------------------------

Combined | 1.2497377 1.1206426 1.3937043

2

------------------------------------------------------------------------------

Study ommited | e^coef. [95% Conf. Interval]

-------------------+----------------------------------------------------------

Buck D [42] | 1.5741544 1.1595894 2.1369306

Sokolova EA[44] | 1.8649942 1.1946425 2.9115017

Wagner M [45] | 1.4149871 1.0443771 1.9171125

Field J [46] | 1.600765 1.2096105 2.118408

-------------------+----------------------------------------------------------

Combined | 1.5706507 1.1915897 2.0702962

3

------------------------------------------------------------------------------

Study ommited | e^coef. [95% Conf. Interval]

-------------------+----------------------------------------------------------

Buck D [42] | 1.2521164 1.0759721 1.4570969

**Sokolova EA[44] | 1.2484217 .97918003 1.5916959**

Wagner M [45] | 1.1890382 1.0159959 1.3915527

Field J [46] | 1.2308909 1.0671298 1.4197828

-------------------+----------------------------------------------------------

Combined | 1.2274883 1.0655821 1.4139947

4

------------------------------------------------------------------------------

Study ommited | e^coef. [95% Conf. Interval]

-------------------+----------------------------------------------------------

Buck D [42] | 1.2953839 1.1209718 1.4969327

Sokolova EA[44] | 1.3424724 1.0659595 1.6907134

Wagner M [45] | 1.2229498 1.0530003 1.4203285

Field J [46] | 1.2829576 1.1197685 1.4699291

-------------------+----------------------------------------------------------

Combined | 1.2765604 1.1156735 1.4606482

5

------------------------------------------------------------------------------

Study ommited | e^coef. [95% Conf. Interval]

-------------------+----------------------------------------------------------

Buck D [42] | 1.4433726 1.0692448 1.9484074

Sokolova EA[44] | 1.7170163 1.1143317 2.6456618

**Wagner M [45] | 1.3287229 .98740684 1.7880214**

Field J [46] | 1.4808164 1.1258623 1.9476781

-------------------+----------------------------------------------------------

Combined | 1.4550338 1.1106237 1.9062472

MS-PB-sub

1

------------------------------------------------------------------------------

Study ommited | e^coef. [95% Conf. Interval]

-------------------+----------------------------------------------------------

Buck D [42] | 1.1580623 1.0586442 1.2668168

Blanco KF [43] | 1.3311474 1.1092598 1.5974198

Wagner M [45] | 1.1222438 1.0244636 1.2293567

Field J [46] | 1.1643426 1.0682216 1.2691128

-------------------+----------------------------------------------------------

Combined | 1.1612845 1.0659925 1.2650949

2

------------------------------------------------------------------------------

Study ommited | e^coef. [95% Conf. Interval]

-------------------+----------------------------------------------------------

Buck D [42] | 1.2675481 1.0240465 1.5689503

Blanco KF [43] | 1.8649942 1.1946425 2.9115017

**Wagner M [45] | 1.2046293 .97386412 1.4900762**

Field J [46] | 1.3025645 1.0621028 1.5974673

-------------------+----------------------------------------------------------

Combined | 1.2933703 1.0562462 1.583728

3

------------------------------------------------------------------------------

Study ommited | e^coef. [95% Conf. Interval]

-------------------+----------------------------------------------------------

Buck D [42] | 1.1877079 1.052931 1.3397365

**Blanco KF [43] | 1.2484217 .97918003 1.5916959**

Wagner M [45] | 1.147773 1.0147006 1.2982971

Field J [46] | 1.1790443 1.0500494 1.3238859

-------------------+----------------------------------------------------------

Combined | 1.1774995 1.0494156 1.3212164

4

------------------------------------------------------------------------------

Study ommited | e^coef. [95% Conf. Interval]

-------------------+----------------------------------------------------------

Buck D [42] | 1.2032088 1.0732414 1.3489149

Blanco KF [43] | 1.3424724 1.0659595 1.6907134

Wagner M [45] | 1.1581195 1.0305183 1.3015207

Field J [46] | 1.2023023 1.0771335 1.3420164

-------------------+----------------------------------------------------------

Combined | 1.1993571 1.075256 1.3377813

5

------------------------------------------------------------------------------

Study ommited | e^coef. [95% Conf. Interval]

-------------------+----------------------------------------------------------

Buck D [42] | 1.1797897 .95942324 1.4507713

**Blanco KF [43] | 1.7170163 1.1143317 2.6456618**

Wagner M [45] | 1.1361124 .92486154 1.3956159

Field J [46] | 1.2165757 .99839436 1.4824366

-------------------+----------------------------------------------------------

Combined | 1.2089278 .99359976 1.4709207

GD

1

------------------------------------------------------------------------------

Study ommited | e^coef. [95% Conf. Interval]

-------------------+----------------------------------------------------------

Tomer Y [23] | .74141056 .66516852 .82639152

Kim TY [24] | .74862835 .67328235 .83240621

Heward JM [25] | .72718711 .64940809 .81428165

Houston F [26] | .7224751 .65084253 .80199165

Mukai T [27] | .72736521 .65156734 .81198076

Kurylowicz A[28] | .72775492 .65048597 .81420238

Luo H [29] | .74784202 .67252205 .83159756

Meng F [30] | .73912958 .66164034 .82569411

Ban Y [31] | .73090622 .65408439 .81675073

Jacobson E [32] | .74811719 .6722959 .83248958

Sun L [33] | .7304342 .652387 .81781843

Makni K [34] | .73326947 .66146086 .81287366

Hsiao JY [35] | .72531799 .65204295 .80682751

Su Y [36] | .75512743 .68250119 .83548197

Ma L [37] | .75133828 .67677113 .83412129

Yang J [38] | .73636414 .65856074 .82335936

Inoue N [39] | .74096907 .66514383 .82543826

Huang J [40] | .74773322 .67208185 .8319001

Chen X [41] | .74382506 .66643899 .8301971

-------------------+----------------------------------------------------------

Combined | .73815803 .66448817 .81999546

2

------------------------------------------------------------------------------

Study ommited | e^coef. [95% Conf. Interval]

-------------------+----------------------------------------------------------

Tomer Y [23] | .54542451 .43135104 .68966544

Kim TY [24] | .55364463 .43632387 .70251112

Heward JM [25] | .51619634 .40376873 .65992893

Houston F [26] | .51177156 .40689532 .64367938

Mukai T [27] | .51881386 .40493071 .66472563

Kurylowicz A[28] | .5180808 .40511829 .6625416

Luo H [29] | .54250898 .42382913 .69442134

Meng F [30] | .53595233 .41713123 .68861999

Ban Y [31] | .53221483 .41384442 .68444231

Jacobson E [32] | .5516118 .4344554 .7003609

Sun L [33] | .52382114 .40561857 .67646949

Makni K [34] | .53201524 .41991952 .67403443

Hsiao JY [35] | .51718955 .40736645 .65662019

Su Y [36] | .55747012 .4403752 .70570035

Ma L [37] | .5758507 .4652424 .71275538

Yang J [38] | .54095012 .42269776 .69228431

Inoue N [39] | .54182124 .42489007 .69093225

Huang J [40] | .54140017 .42211975 .69438623

Chen X [41] | .54077535 .42031057 .69576642

-------------------+----------------------------------------------------------

Combined | .5366406 .42400387 .67919931

3

------------------------------------------------------------------------------

Study ommited | e^coef. [95% Conf. Interval]

-------------------+----------------------------------------------------------

Tomer Y [23] | .78972731 .72134758 .86458904

Kim TY [24] | .79354519 .72481147 .86879691

Heward JM [25] | .78039477 .70711238 .86127186

Houston F [26] | .7699285 .70063962 .84606961

Mukai T [27] | .77854002 .71029442 .85334271

Kurylowicz A[28] | .78099079 .70964197 .85951317

Luo H [29] | .79366854 .72532548 .86845115

Meng F [30] | .79744495 .72832948 .87311919

Ban Y [31] | .77059158 .70332534 .84429117

Jacobson E [32] | .79711475 .72732881 .87359653

Sun L [33] | .77821334 .70924349 .85389011

Makni K [34] | .78298375 .71606551 .85615568

Hsiao JY [35] | .77922223 .71157242 .85330356

Su Y [36] | .7978323 .72913982 .87299632

Ma L [37] | .7912739 .72325053 .86569503

Yang J [38] | .78730052 .71817548 .86307891

Inoue N [39] | .78558629 .71821604 .85927602

Huang J [40] | .79569042 .72705293 .87080765

Chen X [41] | .78916793 .72043962 .86445277

-------------------+----------------------------------------------------------

Combined | .78642026 .7193721 .85971756

4

------------------------------------------------------------------------------

Study ommited | e^coef. [95% Conf. Interval]

-------------------+----------------------------------------------------------

Tomer Y [23] | .70852794 .61740065 .81310545

Kim TY [24] | .71786519 .62777327 .82088623

Heward JM [25] | .69305856 .59905957 .80180703

Houston F [26] | .68841784 .60117468 .78832182

Mukai T [27] | .6939034 .60398435 .79720927

Kurylowicz A[28] | .69376434 .60076269 .80116321

Luo H [29] | .71920776 .63000883 .8210358

Meng F [30] | .71766922 .62745225 .82085785

Ban Y [31] | .69162571 .60381305 .79220898

Jacobson E [32] | .71707639 .6259294 .82149608

Sun L [33] | .6957848 .6039029 .80164625

Makni K [34] | .70010337 .61477204 .79727882

Hsiao JY [35] | .69324094 .60539526 .79383344

Su Y [36] | .73014662 .64511548 .82638551

Ma L [37] | .71816781 .62956023 .81924647

Yang J [38] | .70335674 .61093742 .80975675

Inoue N [39] | .70595708 .61610641 .80891123

Huang J [40] | .72140256 .63240238 .82292804

Chen X [41] | .71166424 .6199274 .8169763

-------------------+----------------------------------------------------------

Combined | .70642487 .61894068 .80627452

5

------------------------------------------------------------------------------

Study ommited | e^coef. [95% Conf. Interval]

-------------------+----------------------------------------------------------

Tomer Y [23] | .6642753 .58202897 .75814382

Kim TY [24] | .67194405 .58769469 .76827103

Heward JM [25] | .63721082 .55473354 .73195074

Houston F [26] | .63801449 .55729848 .73042097

Mukai T [27] | .64470146 .56162051 .74007264

Kurylowicz A[28] | .64507938 .56280337 .73938328

Luo H [29] | .66477913 .58070609 .76102405

Meng F [30] | .65267608 .56926203 .7483128

Ban Y [31] | .67091408 .58551002 .76877542

Jacobson E [32] | .67047674 .58668431 .76623671

Sun L [33] | .65295853 .56745556 .75134488

Makni K [34] | .65902214 .57750451 .75204639

Hsiao JY [35] | .64733895 .56587529 .74053014

Su Y [36] | .67079886 .58623842 .76755651

Ma L [37] | .6888675 .6021656 .78805303

Yang J [38] | .66711398 .5832281 .76306519

Inoue N [39] | .66707155 .58371149 .7623363

Huang J [40] | .66282455 .57834158 .75964863

Chen X [41] | .67447999 .58632952 .77588325

-------------------+----------------------------------------------------------

Combined | .66056259 .5789187 .75372058

GD-HWE-sub

1

------------------------------------------------------------------------------

Study ommited | e^coef. [95% Conf. Interval]

-------------------+----------------------------------------------------------

Tomer Y [23] | .78044811 .70001097 .87012816

Kim TY [24] | .78992107 .71167848 .87676572

Heward JM [25] | .76427973 .68033984 .85857606

Houston F [26] | .75767309 .68165731 .84216585

Mukai T [27] | .7641485 .68315526 .85474411

Kurylowicz A[28] | .76491391 .68187373 .85806692

Meng F [30] | .77887114 .69676863 .87064806

Ban Y [31] | .76857586 .6865665 .86038111

Jacobson E [32] | .79005092 .71128402 .87754039

Sun L [33] | .76840949 .68472732 .86231865

Makni K [34] | .76997241 .69477508 .85330855

Hsiao JY [35] | .76140657 .68375794 .8478731

Su Y [36] | .79714155 .72350199 .8782763

Yang J [38] | .77560092 .69283644 .86825222

Inoue N [39] | .77940824 .69941115 .86855522

Chen X [41] | .78564012 .70451726 .87610402

-------------------+----------------------------------------------------------

Combined | .77479969 .69726208 .86095971

2

------------------------------------------------------------------------------

Study ommited | e^coef. [95% Conf. Interval]

-------------------+----------------------------------------------------------

Tomer Y [23] | .63340987 .54168257 .74067007

Kim TY [24] | .64735774 .55215452 .75897604

Heward JM [25] | .5998883 .50762165 .70892557

Houston F [26] | .59792628 .50879623 .70266999

Mukai T [27] | .61010164 .51767514 .71903011

Kurylowicz A[28] | .6093388 .5174832 .71749919

Meng F [30] | .63746855 .54228967 .74935254

Ban Y [31] | .6330545 .53835867 .74440706

Jacobson E [32] | .64406003 .54961548 .7547337

Sun L [33] | .62118454 .52530496 .73456425

Makni K [34] | .62599846 .53546296 .73184159

Hsiao JY [35] | .61170341 .52142969 .71760598

Su Y [36] | .65233084 .55610404 .76520847

Yang J [38] | .63903626 .54464314 .74978882

Inoue N [39] | .63549677 .54260922 .74428545

Chen X [41] | .65218874 .55289249 .769318

-------------------+----------------------------------------------------------

Combined | .62819385 .53743953 .73427334

3

------------------------------------------------------------------------------

Study ommited | e^coef. [95% Conf. Interval]

-------------------+----------------------------------------------------------

Tomer Y [23] | .81336835 .74068112 .89318878

Kim TY [24] | .81758887 .74449791 .89785553

Heward JM [25] | .80774085 .72898989 .89499907

Houston F [26] | .79359202 .71968263 .87509169

Mukai T [27] | .80170221 .72911014 .88152173

Kurylowicz A[28] | .80672368 .73036864 .89106112

Meng F [30] | .82191799 .74839011 .90266984

Ban Y [31] | .79273272 .7212736 .87127154

Jacobson E [32] | .82212022 .7477794 .90385166

Sun L [33] | .8019277 .72845783 .88280749

Makni K [34] | .80528177 .73430964 .88311346

Hsiao JY [35] | .80195051 .73007999 .88089611

Su Y [36] | .82197887 .74896319 .90211277

Yang J [38] | .81145025 .73784864 .89239375

Inoue N [39] | .80831156 .73681292 .88674827

Chen X [41] | .8130652 .73995443 .89339965

-------------------+----------------------------------------------------------

Combined | .80892703 .73781859 .88688865

4

------------------------------------------------------------------------------

Study ommited | e^coef. [95% Conf. Interval]

-------------------+----------------------------------------------------------

Tomer Y [23] | .77996134 .71332984 .85281682

Kim TY [24] | .78640942 .71912972 .85998362

Heward JM [25] | .76458568 .69331472 .84318312

Houston F [26] | .75340028 .68635213 .82699821

Mukai T [27] | .76736481 .70081754 .84023119

Kurylowicz A[28] | .7667644 .69738301 .84304842

Meng F [30] | .7865894 .71924244 .86024246

Ban Y [31] | .76415928 .69820239 .83634689

Jacobson E [32] | .79009089 .72175422 .86489777

Sun L [33] | .77029007 .70266947 .84441805

Makni K [34] | .77109599 .70608098 .8420975

Hsiao JY [35] | .76665845 .70094686 .8385303

Su Y [36] | .78989749 .72270965 .86333156

Yang J [38] | .77870632 .71118321 .8526404

Inoue N [39] | .77640287 .71066874 .84821714

Chen X [41] | .78466617 .7170504 .85865792

-------------------+----------------------------------------------------------

Combined | .77503048 .7098441 .84620305

5

------------------------------------------------------------------------------

Study ommited | e^coef. [95% Conf. Interval]

-------------------+----------------------------------------------------------

Tomer Y [23] | .70507295 .61088576 .81378205

Kim TY [24] | .71608503 .61900453 .82839098

Heward JM [25] | .67559332 .58049027 .78627732

Houston F [26] | .67436387 .58196878 .78142787

Mukai T [27] | .68465182 .58877583 .79614022

Kurylowicz A[28] | .6840572 .58942955 .79387647

Meng F [30] | .69387946 .5976622 .80558668

Ban Y [31] | .71656524 .61766941 .8312954

Jacobson E [32] | .71386636 .61745572 .82533073

Sun L [33] | .69698482 .59750168 .81303176

Makni K [34] | .69841739 .60522399 .80596085

Hsiao JY [35] | .68554497 .59220151 .79360134

Su Y [36] | .71526468 .61767825 .82826869

Yang J [38] | .71032686 .61369906 .82216886

Inoue N [39] | .70957713 .61375495 .82035951

Chen X [41] | .72447764 .62130912 .84477732

-------------------+----------------------------------------------------------

Combined | .70025744 .6069045 .80796977

GD-PB-sub

1

------------------------------------------------------------------------------

Study ommited | e^coef. [95% Conf. Interval]

-------------------+----------------------------------------------------------

Tomer Y [23] | .74761617 .667381 .83749753

Kim TY [24] | .75561568 .67646184 .84403144

Heward JM [25] | .73228662 .64983162 .82520407

Houston F [26] | .72718321 .65139942 .81178367

Mukai T [27] | .73243649 .65237741 .82232034

Kurylowicz A[28] | .73289883 .65113396 .82493116

Luo H [29] | .75471932 .67557041 .8431412

Meng F [30] | .74520966 .66358448 .83687526

Ban Y [31] | .73625511 .6551973 .827341

Jacobson E [32] | .75509574 .67544082 .84414439

Sun L [33] | .73579428 .65331824 .82868223

Makni K [34] | .73849695 .6630928 .82247575

Hsiao JY [35] | .730132 .65286277 .81654639

Su Y [36] | .7628126 .686719 .84733794

Ma L [37] | .75863729 .68038591 .84588839

Yang J [38] | .74220534 .66018611 .83441436

Inoue N [39] | .74708851 .66730982 .83640496

Huang J [40] | .75463179 .67513403 .8434905

-------------------+----------------------------------------------------------

Combined | .74382506 .66643899 .8301971

2

------------------------------------------------------------------------------

Study ommited | e^coef. [95% Conf. Interval]

-------------------+----------------------------------------------------------

Tomer Y [23] | .55089114 .42861693 .70804727

Kim TY [24] | .55995008 .43399598 .72245852

Heward JM [25] | .51863309 .39760218 .67650605

Houston F [26] | .51354895 .40097859 .6577222

Mukai T [27] | .52141448 .39902257 .68134757

Kurylowicz A[28] | .52059601 .39923959 .67884102

Luo H [29] | .5474145 .4200543 .71339025

Meng F [30] | .54013728 .41261984 .70706314

Ban Y [31] | .53602233 .40897917 .70252951

Jacobson E [32] | .55770181 .43193116 .72009464

Sun L [33] | .52684683 .39974192 .69436696

Makni K [34] | .53546244 .41564059 .68982683

Hsiao JY [35] | .51933938 .40165729 .67150129

Su Y [36] | .56422918 .4385177 .72597883

Ma L [37] | .58549927 .46672943 .73449277

Yang J [38] | .54569739 .41880966 .71102858

Inoue N [39] | .54672176 .42129631 .70948801

Huang J [40] | .54615522 .41814636 .71335196

-------------------+----------------------------------------------------------

Combined | .54077535 .42031057 .69576642

3

------------------------------------------------------------------------------

Study ommited | e^coef. [95% Conf. Interval]

-------------------+----------------------------------------------------------

Tomer Y [23] | .79273833 .72257378 .86971611

Kim TY [24] | .79675402 .72620891 .87415201

Heward JM [25] | .78343013 .70792198 .86699211

Houston F [26] | .77211934 .70095889 .85050392

Mukai T [27] | .78105068 .71102466 .8579733

Kurylowicz A[28] | .78388031 .71048583 .86485657

Luo H [29] | .79684264 .72672035 .87373113

Meng F [30] | .80085925 .72990344 .87871286

Ban Y [31] | .77267377 .70370222 .8484054

Jacobson E [32] | .80058804 .72890491 .87932074

Sun L [33] | .78076809 .70995772 .85864101

Makni K [34] | .78557668 .71698654 .86072845

Hsiao JY [35] | .78171481 .71233318 .85785424

Su Y [36] | .80121702 .73071957 .87851583

Ma L [37] | .79431565 .72453742 .87081403

Yang J [38] | .79027751 .71930434 .86825354

Inoue N [39] | .78832794 .71925165 .86403825

Huang J [40] | .79897979 .72854032 .87622975

-------------------+----------------------------------------------------------

Combined | .78916793 .72043962 .86445277

4

------------------------------------------------------------------------------

Study ommited | e^coef. [95% Conf. Interval]

-------------------+----------------------------------------------------------

Tomer Y [23] | .7142375 .6184631 .82484339

Kim TY [24] | .72435964 .62974015 .83319586

Heward JM [25] | .6977298 .59842987 .81350698

Houston F [26] | .69259503 .60054977 .79874791

Mukai T [27] | .69847069 .60382692 .80794892

Kurylowicz A[28] | .6984757 .60035016 .81263958

Luo H [29] | .72578079 .63212911 .83330722

Meng F [30] | .72415254 .62939776 .83317248

Ban Y [31] | .69591447 .60352977 .80244086

Jacobson E [32] | .72356839 .62782265 .83391577

Sun L [33] | .70059311 .60381462 .8128831

Makni K [34] | .7046908 .61522632 .80716496

Hsiao JY [35] | .69759281 .60524135 .80403584

Su Y [36] | .73757497 .64848622 .83890268

Ma L [37] | .724615 .63160203 .83132555

Yang J [38] | .70873541 .61150552 .82142493

Inoue N [39] | .71133392 .61697508 .82012379

Huang J [40] | .72817408 .63474911 .83534973

-------------------+----------------------------------------------------------

Combined | .71166424 .6199274 .8169763

5

------------------------------------------------------------------------------

Study ommited | e^coef. [95% Conf. Interval]

-------------------+----------------------------------------------------------

Tomer Y [23] | .67881582 .58992453 .78110147

Kim TY [24] | .68814972 .59675167 .79354622

Heward JM [25] | .64891981 .55957284 .7525328

Houston F [26] | .64920717 .56210451 .7498071

Mukai T [27] | .65749969 .56742211 .76187698

Kurylowicz A[28] | .65761622 .5685706 .76060755

Luo H [29] | .68017508 .58895306 .78552634

Meng F [30] | .66649833 .5760424 .77115857

Ban Y [31] | .68756471 .59466944 .79497146

Jacobson E [32] | .68632293 .59550075 .79099676

Sun L [33] | .66768669 .57453205 .77594542

Makni K [34] | .67273375 .5847338 .77397733

Hsiao JY [35] | .65981828 .57181877 .76136039

Su Y [36] | .68703613 .59522965 .79300257

Ma L [37] | .70797155 .61353497 .81694399

Yang J [38] | .682665 .59169957 .78761508

Inoue N [39] | .68238066 .59208846 .78644223

Huang J [40] | .67819965 .58643614 .784322

-------------------+----------------------------------------------------------

Combined | .67447999 .58632952 .77588325

GD-欧美-sub

1

------------------------------------------------------------------------------

Study ommited | e^coef. [95% Conf. Interval]

-------------------+----------------------------------------------------------

**Tomer Y [23] | .85838934 .71124207 1.0359796**

**Heward JM [25] | .8194418 .63717249 1.053851**

Houston F [26] | .78669128 .64348454 .9617685

**Kurylowicz A[28] | .81878752 .64285557 1.0428672**

**Jacobson E [32] | .89077903 .77263512 1.0269884**

Makni K [34] | .8176381 .69218842 .96582382

-------------------+----------------------------------------------------------

Combined | .83150633 .69664479 .99247534

------------------------------------------------------------------------------

2

------------------------------------------------------------------------------

Study ommited | e^coef. [95% Conf. Interval]

-------------------+----------------------------------------------------------

Tomer Y [23] | .81251114 .52541411 1.2564839

Heward JM [25] | .66663449 .32759223 1.3565693

Houston F [26] | .63120741 .36183328 1.1011226

Kurylowicz A[28] | .67515155 .34218229 1.3321251

Jacobson E [32] | .91762535 .64904223 1.2973521

Makni K [34] | .72009681 .43910367 1.1809043

-------------------+----------------------------------------------------------

Combined | .74537756 .46307229 1.1997861

3

------------------------------------------------------------------------------

Study ommited | e^coef. [95% Conf. Interval]

-------------------+----------------------------------------------------------

Tomer Y [23] | .82429363 .72521984 .93690211

Heward JM [25] | .81636159 .69951216 .95273004

Houston F [26] | .78601973 .68379197 .90353067

Kurylowicz A[28] | .81309785 .70383801 .93931857

Jacobson E [32] | .84188017 .73845127 .95979552

Makni K [34] | .8088297 .71403653 .91620729

-------------------+----------------------------------------------------------

Combined | .81546044 .72033396 .92314922

4

------------------------------------------------------------------------------

Study ommited | e^coef. [95% Conf. Interval]

-------------------+----------------------------------------------------------

Tomer Y [23] | .82562943 .72998098 .93381057

Heward JM [25] | .80951576 .69741023 .93964175

Houston F [26] | .77667615 .6792578 .88806613

Kurylowicz A[28] | .80845841 .70351646 .92905432

Jacobson E [32] | .84909823 .74840488 .96333926

Makni K [34] | .80599026 .71485381 .90874567

-------------------+----------------------------------------------------------

Combined | .8132646 .7217164 .91642549

5

------------------------------------------------------------------------------

Study ommited | e^coef. [95% Conf. Interval]

-------------------+----------------------------------------------------------

Tomer Y [23] | .91389996 .69529963 1.2012277

Heward JM [25] | .85887058 .60556042 1.2181422

Houston F [26] | .80416162 .59319072 1.0901652

Kurylowicz A[28] | .87303223 .63489163 1.2004967

Jacobson E [32] | .98214992 .73805782 1.3069687

Makni K [34] | .88112975 .67116838 1.1567733

-------------------+----------------------------------------------------------

Combined | .88803237 .67708177 1.1647064

GD-Asian-sub

1

------------------------------------------------------------------------------

Study ommited | e^coef. [95% Conf. Interval]

-------------------+----------------------------------------------------------

Kim TY [24] | .71120288 .62659247 .80723846

Mukai T [27] | .68203281 .60169908 .773092

Luo H [29] | .71036658 .62596976 .80614225

Meng F [30] | .69745299 .6100139 .79742556

Ban Y [31] | .68655464 .60239254 .78247528

Sun L [33] | .6853059 .60011696 .78258775

Hsiao JY [35] | .68115763 .60523812 .76660029

Su Y [36] | .72072555 .64094824 .81043256

Ma L [37] | .71495178 .63150573 .80942424

Yang J [38] | .69349888 .60606727 .79354341

Inoue N [39] | .70164449 .6169756 .79793268

Huang J [40] | .70987284 .62475605 .80658594

Chen X [41] | .70326968 .61502001 .80418236

-------------------+----------------------------------------------------------

Combined | .69983339 .61900229 .79121966

2

------------------------------------------------------------------------------

Study ommited | e^coef. [95% Conf. Interval]

-------------------+----------------------------------------------------------

Kim TY [24] | .52206843 .43840585 .62169664

Mukai T [27] | .47716457 .39791777 .57219367

Luo H [29] | .514603 .43133933 .6139395

Meng F [30] | .50795654 .42506352 .60701479

Ban Y [31] | .5031245 .42083589 .60150351

Sun L [33] | .48178465 .3999317 .58039023

Hsiao JY [35] | .48659324 .40828982 .579914

Su Y [36] | .52600932 .44140787 .62682572

Ma L [37] | .53471184 .44931864 .63633407

Yang J [38] | .51269695 .43010308 .61115156

Inoue N [39] | .51233599 .43083219 .60925849

Huang J [40] | .51442693 .43042979 .61481587

Chen X [41] | .51610518 .42987136 .61963782

-------------------+----------------------------------------------------------

Combined | .50856265 .42877544 .60319679

3

------------------------------------------------------------------------------

Study ommited | e^coef. [95% Conf. Interval]

-------------------+----------------------------------------------------------

Kim TY [24] | .76920314 .67364806 .87831244

Mukai T [27] | .73617323 .64236356 .8436827

Luo H [29] | .77007356 .67557077 .87779594

Meng F [30] | .77727231 .68058573 .88769456

Ban Y [31] | .72062282 .62961773 .8247818

Sun L [33] | .73330031 .63758598 .84338326

Hsiao JY [35] | .73934538 .64705025 .84480545

Su Y [36] | .77865092 .68312179 .88753903

Ma L [37] | .7653579 .67175684 .87200113

Yang J [38] | .75434506 .65788362 .86495005

Inoue N [39] | .7541492 .66237963 .85863301

Huang J [40] | .77406166 .67872301 .88279231

Chen X [41] | .75942619 .66399322 .86857535

-------------------+----------------------------------------------------------

Combined | .7565816 .66560829 .85998887

4

------------------------------------------------------------------------------

Study ommited | e^coef. [95% Conf. Interval]

-------------------+----------------------------------------------------------

Kim TY [24] | .66470481 .55086863 .80206508

Mukai T [27] | .63076158 .52258276 .7613343

Luo H [29] | .66792817 .55569938 .80282265

Meng F [30] | .66422201 .55015881 .80193369

Ban Y [31] | .6282036 .52473371 .75207626

Sun L [33] | .63286859 .52076301 .76910733

Hsiao JY [35] | .63044729 .52583026 .75587849

Su Y [36] | .68467503 .57896502 .80968605

Ma L [37] | .66784701 .5570175 .80072821

Yang J [38] | .64274206 .52702392 .78386832

Inoue N [39] | .64999173 .53864545 .784355

Huang J [40] | .67035964 .55809514 .80520688

Chen X [41] | .65448993 .538426 .79557278

-------------------+----------------------------------------------------------

Combined | .65302113 .54615344 .78079999

5

------------------------------------------------------------------------------

Study ommited | e^coef. [95% Conf. Interval]

-------------------+----------------------------------------------------------

Kim TY [24] | .61411816 .52642631 .71641768

Mukai T [27] | .57651806 .49115781 .6767134

Luo H [29] | .60406985 .51683049 .70603494

Meng F [30] | .58764978 .5015956 .6884675

Ban Y [31] | .61043409 .5215137 .7145158

Sun L [33] | .58350427 .4952342 .68750751

Hsiao JY [35] | .58392504 .50014128 .68174427

Su Y [36] | .61187976 .52388756 .71465115

Ma L [37] | .6340933 .5430945 .7403395

Yang J [38] | .60782418 .52070169 .70952378

Inoue N [39] | .60876181 .52221298 .7096548

Huang J [40] | .60039505 .51278796 .70296933

Chen X [41] | .61027453 .51819521 .71871563

-------------------+----------------------------------------------------------

Combined | .60266871 .51820398 .70090077

GD-Asian-HWE-sub

1

------------------------------------------------------------------------------

Study ommited | e^coef. [95% Conf. Interval]

-------------------+----------------------------------------------------------

Kim TY [24] | .76331521 .66966875 .87005719

Mukai T [27] | .72300887 .62934098 .83061782

Meng F [30] | .74532653 .64452592 .86189184

Ban Y [31] | .72963963 .63151897 .84300555

Sun L [33] | .7285979 .62788188 .84546937

Hsiao JY [35] | .72102118 .63464545 .81915271

Su Y [36] | .77441482 .6911685 .86768757

Yang J [38] | .73999358 .6384067 .85774554

Inoue N [39] | .74809249 .65180297 .85860667

Chen X [41] | .75517418 .65508768 .87055223

-------------------+----------------------------------------------------------

Combined | .74301003 .65204445 .84666605

2

------------------------------------------------------------------------------

Study ommited | e^coef. [95% Conf. Interval]

-------------------+----------------------------------------------------------

Kim TY [24] | .57538156 .47356842 .69908365

Mukai T [27] | .51852883 .42261556 .63620977

Meng F [30] | .55867868 .45757681 .68211908

Ban Y [31] | .55233051 .45209885 .67478382

Sun L [33] | .52787282 .42739237 .65197635

Hsiao JY [35] | .52766575 .43381512 .64181982

Su Y [36] | .58135878 .47802588 .70702872

Yang J [38] | .56332235 .46299533 .68538935

Inoue N [39] | .56105417 .46266528 .68036611

Chen X [41] | .57378924 .46684564 .70523115

-------------------+----------------------------------------------------------

Combined | .55412569 .45858771 .66956717

3

------------------------------------------------------------------------------

Study ommited | e^coef. [95% Conf. Interval]

-------------------+----------------------------------------------------------

Kim TY [24] | .82041926 .71122959 .94637198

Mukai T [27] | .78266165 .67538077 .90698357

Meng F [30] | .83058699 .71987359 .95832763

Ban Y [31] | .76237989 .65896593 .882023

Sun L [33] | .78203898 .67179479 .9103747

Hsiao JY [35] | .78417223 .6792174 .90534501

Su Y [36] | .83043481 .72148828 .95583254

Yang J [38] | .8057696 .69487164 .93436631

Inoue N [39] | .79939417 .69549924 .9188091

Chen X [41] | .80980882 .700588 .93605704

-------------------+----------------------------------------------------------

Combined | .80100452 .69831166 .91879926

4

------------------------------------------------------------------------------

Study ommited | e^coef. [95% Conf. Interval]

-------------------+----------------------------------------------------------

Kim TY [24] | .75339903 .65828427 .86225682

Mukai T [27] | .70890402 .6166136 .81500782

Meng F [30] | .75367356 .65836076 .86278507

Ban Y [31] | .70329371 .61271966 .80725669

Sun L [33] | .71195939 .61658158 .82209101

Hsiao JY [35] | .71052582 .62045225 .81367574

Su Y [36] | .76179535 .66684302 .87026801

Yang J [38] | .73394777 .63842215 .84376666

Inoue N [39] | .73382367 .6432789 .83711308

Chen X [41] | .74839421 .65237075 .8585515

-------------------+----------------------------------------------------------

Combined | .73223169 .64315639 .83364366

5

------------------------------------------------------------------------------

Study ommited | e^coef. [95% Conf. Interval]

-------------------+----------------------------------------------------------

Kim TY [24] | .65623465 .552138 .77995703

Mukai T [27] | .60933772 .50817671 .73063652

Meng F [30] | .62333791 .52128691 .74536719

Ban Y [31] | .65365941 .54735306 .78061247

Sun L [33] | .62109494 .51511377 .74888103

Hsiao JY [35] | .61638392 .51801898 .73342707

Su Y [36] | .65406136 .54939061 .77867414

Yang J [38] | .64822145 .54490951 .77112078

Inoue N [39] | .64856101 .54623776 .77005183

Chen X [41] | .65805785 .54614507 .79290313

-------------------+----------------------------------------------------------

Combined | .6389606 .53992198 .75616602

GD-Asian-PB-sub

1

------------------------------------------------------------------------------

Study ommited | e^coef. [95% Conf. Interval]

-------------------+----------------------------------------------------------

Kim TY [24] | .71648165 .62370242 .82306231

Mukai T [27] | .68358225 .59468201 .78577237

Luo H [29] | .715561 .62300854 .8218628

Meng F [30] | .70078829 .60457405 .81231443

Ban Y [31] | .68855116 .59568437 .79589582

Sun L [33] | .68737322 .59296401 .79681386

Hsiao JY [35] | .68208999 .5983741 .7775182

Su Y [36] | .72762978 .64042433 .82670983

Ma L [37] | .72079401 .629392 .82546967

Yang J [38] | .69635764 .60001393 .80817118

Inoue N [39] | .70554716 .61269215 .81247459

Huang J [40] | .71494211 .62157529 .82233355

-------------------+----------------------------------------------------------

Combined | .70326968 .61502001 .80418236

2

------------------------------------------------------------------------------

Study ommited | e^coef. [95% Conf. Interval]

-------------------+----------------------------------------------------------

Kim TY [24] | .50020777 .3780424 .6618512

Mukai T [27] | .45340491 .34278697 .59971945

Luo H [29] | .48452109 .36045608 .65128791

Meng F [30] | .47536603 .35173169 .64245806

Ban Y [31] | .47043393 .34808132 .63579419

Sun L [33] | .4589002 .34008703 .61922207

Hsiao JY [35] | .45503387 .34896665 .59333986

Su Y [36] | .50521159 .38331984 .66586367

Ma L [37] | .53202449 .42326934 .66872327

Yang J [38] | .4828132 .35955933 .64831744

Inoue N [39] | .48529793 .36402541 .64697153

Huang J [40] | .4824544 .35746679 .6511437

-------------------+----------------------------------------------------------

Combined | .48186838 .36701651 .6326613

3

------------------------------------------------------------------------------

Study ommited | e^coef. [95% Conf. Interval]

-------------------+----------------------------------------------------------

Kim TY [24] | .77369143 .67292005 .88955357

Mukai T [27] | .73706038 .63831917 .85107582

Luo H [29] | .77452968 .67499607 .88874032

Meng F [30] | .78269614 .68060156 .90010556

Ban Y [31] | .71976539 .62429817 .82983138

Sun L [33] | .73389163 .632914 .85097963

Hsiao JY [35] | .74053749 .64356373 .8521235

Su Y [36] | .78405322 .68332622 .89962809

Ma L [37] | .76926464 .6707814 .88220705

Yang J [38] | .75737031 .65551448 .87505281

Inoue N [39] | .75681763 .66045946 .86723403

Huang J [40] | .77900037 .6784904 .89439964

-------------------+----------------------------------------------------------

Combined | .75942619 .66399322 .86857535

4

------------------------------------------------------------------------------

Study ommited | e^coef. [95% Conf. Interval]

-------------------+----------------------------------------------------------

Kim TY [24] | .6676485 .54323628 .82055367

Mukai T [27] | .62978817 .51122667 .77584595

Luo H [29] | .67142801 .54878902 .82147339

Meng F [30] | .66708565 .54242224 .82040012

Ban Y [31] | .62665983 .51333203 .7650069

Sun L [33] | .63232735 .50924328 .78516084

Hsiao JY [35] | .62893511 .51461607 .76864948

Su Y [36] | .69047883 .57496906 .82919421

Ma L [37] | .67153434 .5504762 .81921502

Yang J [38] | .64312674 .51645448 .80086826

Inoue N [39] | .65114678 .52979693 .80029179

Huang J [40] | .6740612 .55136674 .8240586

-------------------+----------------------------------------------------------

Combined | .65448993 .538426 .79557278

5

------------------------------------------------------------------------------

Study ommited | e^coef. [95% Conf. Interval]

-------------------+----------------------------------------------------------

Kim TY [24] | .62437929 .52809143 .73822348

Mukai T [27] | .57975007 .48643361 .69096818

Luo H [29] | .61256974 .51683658 .72603546

Meng F [30] | .59304548 .49883088 .70505448

Ban Y [31] | .62045954 .52250329 .73678013

Sun L [33] | .58845878 .49127754 .70486377

Hsiao JY [35] | .58832287 .49707975 .69631442

Su Y [36] | .62187154 .52517025 .73637875

Ma L [37] | .64863544 .54801672 .7677283

Yang J [38] | .61691285 .52135228 .72998907

Inoue N [39] | .61784671 .52305214 .72982123

Huang J [40] | .60835879 .51211932 .72268395

-------------------+----------------------------------------------------------

Combined | .61027453 .51819521 .71871563

Overall

1

------------------------------------------------------------------------------

Study ommited | e^coef. [95% Conf. Interval]

-------------------+----------------------------------------------------------

Tomer Y [23] | .93654657 .87738291 .99969976

**Kim TY [24] | .94044145 .88180086 1.0029817**

Heward JM [25] | .93310084 .8730259 .99730968

Houston F [26] | .92940489 .86957453 .99335183

Mukai T [27] | .93190226 .8721196 .99578295

Kurylowicz A[28] | .93295853 .87301021 .99702342

**Luo H [29] | .93974752 .88101188 1.002399**

**Meng F [30] | .93701461 .87773308 1.0003**

Ban Y [31] | .93335059 .87368806 .99708737

**Jacobson E [32] | .94077795 .88217859 1.0032698**

Sun L [33] | .93414161 .87436639 .99800331

Makni K [34] | .92952669 .87092903 .99206692

Hsiao JY [35] | .92975432 .87026766 .99330716

**Su Y [36] | .94274389 .88467433 1.0046251**

**Ma L [37] | .94171867 .88334412 1.0039508**

Yang J [38] | .93607723 .87664339 .9995405

Inoue N [39] | .93568255 .87654264 .9988126

**Huang J [40] | .94014624 .88143919 1.0027634**

**Chen X [41] | .93985802 .88107861 1.0025588**

Kim TY [24] | .92942016 .87004158 .99285121

Ban Y [31] | .92951061 .87001149 .99307882

Yang J [38] | .92790635 .86856283 .99130446

Inoue N [39] | .93018583 .87103973 .99334811

Buck D [42] | .92704773 .86787895 .99025041

Blanco KF [43] | .92584257 .86539221 .99051558

Sokolova EA[44] | .92502952 .86589983 .98819699

Wagner M [45] | .92299552 .86519987 .98465193

Field J [46] | .93151446 .87243768 .99459161

Teruel M [47] | .92908858 .8682973 .99413599

Teruel M [47] | .92817285 .8684464 .99200692

Teruel M [47] | .92893854 .86922641 .99275265

Teruel M [47] | .92842904 .86837208 .99263957

Joo YB [48] | .9262359 .86656802 .99001223

Zhu Q [49] | .93511069 .87576609 .99847667

Wu C [50] | .92321985 .86532796 .9849848

Park JH [51] | .92856707 .86909088 .99211351

Hsieh YY [52] | .93016221 .87091535 .99343953

Du J [53] | .93419268 .8747502 .9976745

Liu R [54] | .92838294 .86878302 .99207151

García BM [55] | .93221484 .87156858 .99708103

Chen F [56] | .92567039 .86654132 .98883418

Inal EE [57] | .93200807 .87229216 .99581206

Jacobson E [32] | .93129069 .87188215 .99474723

Blanco KF [43] | .92505901 .86577399 .98840364

Blanco KF [43] | .92768094 .86690892 .9927132

Tanizawa K [58] | .92848008 .86915801 .99185103

Chen F [59] | .92830407 .86890145 .99176776

Chen F [56] | .92787392 .86806898 .99179906

Pu T [60] | .9314453 .87209054 .99483977

Rodríguez [61] | .92689696 .86742799 .99044298

**Wei Y [62] | .94545529 .88806483 1.0065546**

-------------------+----------------------------------------------------------

Combined | .93171324 .87295667 .99442457

2

------------------------------------------------------------------------------

Study ommited | e^coef. [95% Conf. Interval]

-------------------+----------------------------------------------------------

Tomer Y [23] | .87921128 .76643433 1.0085828

Kim TY [24] | .88936106 .77603491 1.0192365

Heward JM [25] | .87195491 .75716766 1.004144

**Houston F [26] | .86560054 .75250333 .99569564**

Mukai T [27] | .87311225 .75852251 1.005013

Kurylowicz A[28] | .87217483 .75775353 1.0038738

Luo H [29] | .88519306 .77121731 1.0160129

Meng F [30] | .88263499 .76835922 1.0139067

Ban Y [31] | .88067252 .76626259 1.0121649

Jacobson E [32] | .88757018 .77414286 1.0176169

Sun L [33] | .87796249 .76308183 1.0101382

**Makni K [34] | .87074272 .7583003 .99985833**

**Hsiao JY [35] | .86864712 .75526813 .99904629**

Su Y [36] | .89198178 .77895051 1.0214147

Ma L [37] | .89930846 .78803963 1.0262881

Yang J [38] | .88363932 .76961559 1.0145564

Inoue N [39] | .88143626 .76769017 1.0120357

Huang J [40] | .88581311 .77180707 1.0166593

Chen X [41] | .88777577 .77399975 1.0182766

**Kim TY [24] | .86820491 .7547993 .99864927**

Ban Y [31] | .86997079 .75607009 1.0010304

**Yang J [38] | .86340915 .75101565 .99262293**

**Inoue N [39] | .8694865 .75642859 .99944237**

**Buck D [42] | .86287687 .75063572 .99190123**

**Blanco KF [43] | .86263389 .74725742 .99582447**

**Sokolova EA[44] | .86093645 .7482968 .99053153**

**Wagner M [45] | .85662533 .74718296 .98209809**

Field J [46] | .87270951 .75963788 1.0026118

Teruel M [47] | .86676952 .75126851 1.0000278

**Teruel M [47] | .85957945 .7482843 .98742794**

Teruel M [47] | .87583997 .76150532 1.0073411

**Teruel M [47] | .86635849 .75205582 .99803368**

**Joo YB [48] | .86391282 .74982831 .995355**

Zhu Q [49] | .87698656 .76289213 1.0081444

**Wu C [50] | .85646549 .74687289 .98213919**

**Park JH [51] | .86874866 .75495505 .99969425**

Hsieh YY [52] | .87094416 .75748254 1.0014009

Du J [53] | .87606022 .76195123 1.0072581

**Liu R [54] | .86595558 .75242615 .99661484**

García BM [55] | .87587423 .76015734 1.0092064

**Chen F [56] | .85960074 .74788325 .9880064**

Inal EE [57] | .8730275 .75879986 1.0044506

Jacobson E [32] | .87137999 .75790907 1.0018393

**Blanco KF [43] | .8607556 .7474227 .99127334**

Blanco KF [43] | .87004424 .75413323 1.0037709

**Tanizawa K [58] | .86727565 .75401239 .99755265**

**Chen F [59] | .8654098 .75221209 .99564224**

**Chen F [56] | .86459289 .75066166 .99581596**

Pu T [60] | .87241714 .75873313 1.0031349

**Rodríguez [61] | .86077176 .74862488 .98971868**

Wei Y [62] | .89637162 .78404671 1.0247885

-------------------+----------------------------------------------------------

Combined | .87266234 .7602544 1.0016904

3

------------------------------------------------------------------------------

Study ommited | e^coef. [95% Conf. Interval]

-------------------+----------------------------------------------------------

Tomer Y [23] | .94809919 .87929719 1.0222847

Kim TY [24] | .95071923 .88224317 1.0245101

Heward JM [25] | .94809743 .87855625 1.0231431

Houston F [26] | .94297508 .87330781 1.0182

Mukai T [27] | .94334708 .87407724 1.0181065

Kurylowicz A[28] | .94725941 .87771312 1.0223163

Luo H [29] | .95086082 .88268468 1.0243027

Meng F [30] | .95350876 .88551137 1.0267276

Ban Y [31] | .93930104 .87052753 1.0135078

Jacobson E [32] | .95285843 .88447855 1.0265248

Sun L [33] | .94439865 .8749435 1.0193673

Makni K [34] | .94144604 .8733084 1.0148999

Hsiao JY [35] | .94227015 .87326847 1.016724

Su Y [36] | .95452719 .88717699 1.0269903

Ma L [37] | .9488432 .88043847 1.0225626

Yang J [38] | .94761595 .87847484 1.0221989

Inoue N [39] | .94395702 .87528408 1.0180179

Huang J [40] | .95245598 .88447867 1.0256577

Chen X [41] | .94811725 .87916951 1.0224721

Kim TY [24] | .9414472 .87265838 1.0156584

Ban Y [31] | .9386128 .87016039 1.0124501

Yang J [38] | .94231506 .87317896 1.0169252

Inoue N [39] | .94128651 .872939 1.0149853

Buck D [42] | .94067719 .87164651 1.0151748

Blanco KF [43] | .93533955 .86563464 1.0106574

Sokolova EA[44] | .93513555 .86635006 1.0093824

Wagner M [45] | .93524768 .86741477 1.0083852

Field J [46] | .94278667 .87427095 1.0166719

Teruel M [47] | .94134047 .87071983 1.0176889

Teruel M [47] | .94604385 .87647928 1.0211296

Teruel M [47] | .93649572 .86791393 1.0104968

Teruel M [47] | .94000471 .87020267 1.0154058

Joo YB [48] | .93526383 .86676004 1.0091818

Zhu Q [49] | .95286472 .88508842 1.025831

Wu C [50] | .93524502 .86822148 1.0074425

Park JH [51] | .938264 .86965656 1.0122839

Hsieh YY [52] | .93946011 .87137907 1.0128603

Du J [53] | .95084359 .88258325 1.0243833

Liu R [54] | .94058445 .87142204 1.0152361

García BM [55] | .94268681 .87193948 1.0191744

Chen F [56] | .94617084 .87679426 1.0210369

Inal EE [57] | .94429362 .87499939 1.0190755

Jacobson E [32] | .94356082 .87461122 1.017946

Blanco KF [43] | .93478043 .86615008 1.0088488

Blanco KF [43] | .9355893 .86615019 1.0105953

Tanizawa K [58] | .93927796 .87073949 1.0132113

Chen F [59] | .94185268 .87284262 1.0163189

Chen F [56] | .94116124 .87166696 1.016196

Pu T [60] | .9424224 .87370504 1.0165444

Rodríguez [61] | .94230109 .87271793 1.0174322

Wei Y [62] | .95861194 .89198806 1.0302121

-------------------+----------------------------------------------------------

Combined | .94360677 .8754647 1.0170527

4

------------------------------------------------------------------------------

Study ommited | e^coef. [95% Conf. Interval]

-------------------+----------------------------------------------------------

Tomer Y [23] | .92904906 .85663736 1.0075817

Kim TY [24] | .93335483 .86144442 1.0112681

Heward JM [25] | .92636336 .85305065 1.0059767

Houston F [26] | .92154506 .84835216 1.0010528

Mukai T [27] | .92355612 .85066386 1.0026944

Kurylowicz A[28] | .92593742 .85270393 1.0054605

Luo H [29] | .93317189 .86136725 1.0109623

Meng F [30] | .93339196 .86147007 1.0113184

Ban Y [31] | .9215993 .84888847 1.0005381

Jacobson E [32] | .93445884 .86259137 1.012314

Sun L [33] | .92562591 .85260192 1.0049043

**Makni K [34] | .92067421 .84908322 .99830145**

Hsiao JY [35] | .92162508 .84907572 1.0003734

Su Y [36] | .93719151 .86625889 1.0139324

Ma L [37] | .93182589 .86003674 1.0096074

Yang J [38] | .92855341 .85585979 1.0074214

Inoue N [39] | .92590883 .85362896 1.0043089

Huang J [40] | .93451642 .86285058 1.0121346

Chen X [41] | .93170296 .85943289 1.0100502

**Kim TY [24] | .92086416 .84850947 .99938873**

**Ban Y [31] | .91897013 .84672461 .9973799**

**Yang J [38] | .92048887 .84790503 .99928616**

**Inoue N [39] | .92107149 .84910324 .99913963**

**Buck D [42] | .91885813 .84644532 .9974658**

**Blanco KF [43] | .9152716 .84173225 .99523584**

**Sokolova EA[44] | .91484485 .84257677 .99331138**

**Wagner M [45] | .91350823 .84263083 .99034743**

Field J [46] | .92288191 .85077032 1.0011057

Teruel M [47] | .9203051 .8460421 1.0010867

Teruel M [47] | .92216718 .84891687 1.001738

**Teruel M [47] | .9176777 .84507291 .99652036**

**Teruel M [47] | .91933712 .84599573 .99903666**

**Joo YB [48] | .91539824 .84309298 .99390454**

Zhu Q [49] | .93166295 .8596919 1.0096592

**Wu C [50] | .91324846 .84297704 .98937777**

**Park JH [51] | .91831664 .84598912 .99682778**

**Hsieh YY [52] | .91962149 .84771314 .99762956**

Du J [53] | .92993062 .85770404 1.0082393

**Liu R [54] | .91970843 .84703465 .99861747**

García BM [55] | .92302652 .84876198 1.003789

**Chen F [56] | .92062196 .84766671 .99985618**

Inal EE [57] | .92409071 .85118995 1.0032351

Jacobson E [32] | .92320555 .85065335 1.0019457

**Blanco KF [43] | .91464571 .84242764 .99305476**

**Blanco KF [43] | .91641891 .84271506 .99656889**

**Tanizawa K [58] | .9188514 .84670347 .99714709**

**Chen F [59] | .9204578 .84795315 .999162**

**Chen F [56] | .91969327 .84672499 .99894974**

Pu T [60] | .92262252 .85027133 1.0011302

**Rodríguez [61] | .91943442 .84644946 .9987125**

Wei Y [62] | .9416063 .87149071 1.017363

-------------------+----------------------------------------------------------

Combined | .92352374 .85179829 1.0012888

5

------------------------------------------------------------------------------

Study ommited | e^coef. [95% Conf. Interval]

-------------------+----------------------------------------------------------

Tomer Y [23] | .92284472 .82533642 1.031873

Kim TY [24] | .93057789 .83261662 1.0400648

Heward JM [25] | .91697627 .8173969 1.0286869

Houston F [26] | .91192834 .81383191 1.021849

Mukai T [27] | .91900934 .81940499 1.0307213

Kurylowicz A[28] | .9172988 .81804668 1.028593

Luo H [29] | .92608794 .82714732 1.0368635

Meng F [30] | .92108141 .82162972 1.0325709

Ban Y [31] | .92989436 .83127927 1.0402082

Jacobson E [32] | .92944735 .83153458 1.0388893

Sun L [33] | .92379425 .82406247 1.035596

Makni K [34] | .91681677 .81911896 1.0261672

Hsiao JY [35] | .91496313 .81643917 1.0253765

Su Y [36] | .92972822 .83135068 1.0397472

Ma L [37] | .94316487 .84837734 1.0485428

Yang J [38] | .92713789 .82850718 1.0375102

Inoue N [39] | .92663592 .82829325 1.0366548

Huang J [40] | .92566105 .82649148 1.0367298

Chen X [41] | .93157856 .8330574 1.0417513

Kim TY [24] | .91515839 .81644549 1.0258063

Ban Y [31] | .91909828 .81983609 1.0303787

Yang J [38] | .90985385 .81241944 1.0189737

Inoue N [39] | .91755058 .81897181 1.0279952

Buck D [42] | .91010889 .81262492 1.0192872

Blanco KF [43] | .91080962 .81021557 1.0238931

Sokolova EA[44] | .90871579 .81058324 1.0187287

Wagner M [45] | .90579141 .81048238 1.0123084

Field J [46] | .91841287 .82024762 1.0283263

Teruel M [47] | .91327434 .81303276 1.025875

Teruel M [47] | .90549686 .80981795 1.0124801

Teruel M [47] | .92365918 .82458185 1.0346411

Teruel M [47] | .9133004 .81393335 1.0247984

Joo YB [48] | .91300489 .81322397 1.0250287

Zhu Q [49] | .91605448 .81736041 1.0266656

Wu C [50] | .90714755 .81029765 1.0155733

Park JH [51] | .91721732 .81808945 1.0283565

Hsieh YY [52] | .92043813 .82161332 1.0311497

Du J [53] | .91622812 .81744305 1.026951

Liu R [54] | .91294745 .81415474 1.0237281

García BM [55] | .92292576 .82260418 1.0354822

Chen F [56] | .90369031 .80907001 1.0093764

Inal EE [57] | .91844086 .81929407 1.0295859

Jacobson E [32] | .91713408 .81864661 1.0274701

Blanco KF [43] | .90870711 .80968621 1.0198378

Blanco KF [43] | .91954804 .81891197 1.0325513

Tanizawa K [58] | .91563955 .81686346 1.0263598

Chen F [59] | .91176491 .81338254 1.0220471

Chen F [56] | .91091489 .81189682 1.0220091

Pu T [60] | .91878246 .819996 1.0294699

Rodríguez [61] | .9069103 .8100408 1.015364

Wei Y [62] | .93236897 .83420459 1.0420848

-------------------+----------------------------------------------------------

Combined | .91814503 .82059349 1.0272934

Overall-HWE-sub

1

------------------------------------------------------------------------------

Study ommited | e^coef. [95% Conf. Interval]

-------------------+----------------------------------------------------------

Tomer Y [23] | .94863411 .88547763 1.0162952

Kim TY [24] | .95303518 .89067634 1.01976

Heward JM [25] | .94498088 .88044955 1.0142419

Houston F [26] | .94067471 .87658287 1.0094527

Mukai T [27] | .94352271 .87946059 1.0122513

Kurylowicz A[28] | .94478877 .88046404 1.0138129

Meng F [30] | .94928405 .88594945 1.0171463

Ban Y [31] | .94515359 .88125883 1.013681

Jacobson E [32] | .9534679 .89116166 1.0201303

Sun L [33] | .94611443 .88203671 1.0148472

Makni K [34] | .94050562 .87807856 1.0073709

Hsiao JY [35] | .94097944 .87735173 1.0092216

Su Y [36] | .95549301 .89401228 1.0212017

Yang J [38] | .94825056 .88468582 1.0163824

Inoue N [39] | .94760482 .88447179 1.0152442

Chen X [41] | .95251212 .88990867 1.0195196

Kim TY [24] | .94057018 .87709322 1.0086411

Ban Y [31] | .94070604 .87706539 1.0089645

Yang J [38] | .9388451 .87545368 1.0068267

Inoue N [39] | .94136533 .87818659 1.0090893

Buck D [42] | .93783119 .87470405 1.0055142

Sokolova EA[44] | .93563062 .87301075 1.0027421

**Wagner M [45] | .932972 .87200733 .99819889**

Field J [46] | .94282437 .87972715 1.0104472

Teruel M [47] | .94054797 .8751154 1.010873

Teruel M [47] | .93924529 .87533836 1.0078179

Teruel M [47] | .94011205 .87619545 1.0086912

Teruel M [47] | .93962307 .87525862 1.0087207

Joo YB [48] | .93706533 .87343125 1.0053355

Zhu Q [49] | .94705038 .88362093 1.015033

**Wu C [50] | .93325285 .87209918 .99869476**

Park JH [51] | .93962799 .87603961 1.007832

Hsieh YY [52] | .94136986 .8780585 1.0092462

Du J [53] | .94603302 .88245803 1.0141882

Liu R [54] | .9394507 .87570348 1.0078384

García BM [55] | .94405704 .87864409 1.0143398

Chen F [56] | .93626819 .87332642 1.0037463

Inal EE [57] | .94362352 .87965708 1.0122414

Jacobson E [32] | .94269811 .87916293 1.0108248

Tanizawa K [58] | .93949052 .87610899 1.0074573

Chen F [59] | .93931081 .87582798 1.0073951

Chen F [56] | .93892771 .87493314 1.007603

Pu T [60] | .94285372 .87939071 1.0108967

Rodríguez [61] | .93773435 .8742411 1.0058389

Wei Y [62] | .95857645 .89812299 1.0230991

-------------------+----------------------------------------------------------

Combined | .94294937 .88028717 1.0100721

2

------------------------------------------------------------------------------

Study ommited | e^coef. [95% Conf. Interval]

-------------------+----------------------------------------------------------

Tomer Y [23] | .91226943 .78875246 1.0551289

Kim TY [24] | .9248639 .80089178 1.068026

Heward JM [25] | .90480147 .77821317 1.0519813

Houston F [26] | .89687597 .77263768 1.0410915

Mukai T [27] | .90609556 .77981187 1.0528298

Kurylowicz A[28] | .90492867 .77887108 1.0513882

Meng F [30] | .91731878 .7917247 1.0628363

Ban Y [31] | .91501199 .78916612 1.0609261

Jacobson E [32] | .92266505 .79848126 1.0661625

Sun L [33] | .91202868 .7854322 1.0590301

Makni K [34] | .90236529 .77906978 1.0451735

Hsiao JY [35] | .90044152 .77582065 1.0450804

Su Y [36] | .92802822 .80458836 1.0704062

Yang J [38] | .91830527 .79311009 1.063263

Inoue N [39] | .91539348 .79053559 1.0599715

Chen X [41] | .92367217 .79898351 1.0678196

Kim TY [24] | .89994945 .77528725 1.0446567

Ban Y [31] | .9021646 .77681943 1.0477351

Yang J [38] | .89417197 .77092784 1.0371185

Inoue N [39] | .90124809 .77708506 1.0452499

Buck D [42] | .89352563 .77049962 1.0361953

Sokolova EA[44] | .89146258 .76803002 1.0347324

Wagner M [45] | .88584043 .76688188 1.0232518

Field J [46] | .90484124 .78069927 1.0487235

Teruel M [47] | .89887374 .77123513 1.0476364

Teruel M [47] | .8895424 .76792265 1.0304237

Teruel M [47] | .90918162 .78332177 1.0552639

Teruel M [47] | .89810575 .7721676 1.044584

Joo YB [48] | .89522425 .76963952 1.0413011

Zhu Q [49] | .91037744 .78489038 1.0559272

Wu C [50] | .88564233 .76653908 1.0232516

Park JH [51] | .90071748 .77551095 1.0461387

Hsieh YY [52] | .90307945 .77836907 1.0477709

Du J [53] | .9093047 .78377444 1.05494

Liu R [54] | .89741876 .77257327 1.0424389

García BM [55] | .90981632 .78199812 1.0585265

Chen F [56] | .88969766 .7675901 1.03123

Inal EE [57] | .90584051 .78007635 1.0518804

Jacobson E [32] | .90357666 .77885948 1.0482645

Tanizawa K [58] | .89883926 .77437129 1.0433135

Chen F [59] | .89669034 .77231186 1.0410996

Chen F [56] | .89596435 .77057048 1.0417634

Pu T [60] | .90486495 .77986438 1.0499012

Rodríguez [61] | .89111713 .76830951 1.0335545

Wei Y [62] | .9335031 .81128187 1.0741372

-------------------+----------------------------------------------------------

Combined | .90453195 .78133058 1.0471599

3

------------------------------------------------------------------------------

Study ommited | e^coef. [95% Conf. Interval]

-------------------+----------------------------------------------------------

Tomer Y [23] | .9446536 .87280498 1.0224167

Kim TY [24] | .94733733 .87596526 1.0245247

Heward JM [25] | .9447013 .87158696 1.0239489

Houston F [26] | .93924404 .86622533 1.0184179

Mukai T [27] | .93959742 .86713072 1.0181202

Kurylowicz A[28] | .94385876 .87084106 1.0229988

Meng F [30] | .95012277 .87946252 1.0264602

Ban Y [31] | .93499111 .86334193 1.0125865

Jacobson E [32] | .94960329 .87833784 1.026651

Sun L [33] | .94077395 .86801934 1.0196266

Makni K [34] | .93742125 .86649693 1.0141509

Hsiao JY [35] | .93838646 .8663051 1.0164654

Su Y [36] | .95086755 .88120355 1.0260389

Yang J [38] | .94420862 .8718585 1.0225626

Inoue N [39] | .94023603 .86854751 1.0178416

Chen X [41] | .94470798 .87264452 1.0227225

Kim TY [24] | .93746052 .86567975 1.0151933

Ban Y [31] | .93417561 .86298674 1.0112369

Yang J [38] | .93844221 .86618267 1.0167299

Inoue N [39] | .93727505 .86606793 1.0143368

Buck D [42] | .93658899 .86452978 1.0146544

Sokolova EA[44] | .93008429 .85953631 1.0064226

Wagner M [45] | .92999682 .86004987 1.0056325

Field J [46] | .93896709 .86748802 1.0163359

Teruel M [47] | .93775467 .86341808 1.0184913

Teruel M [47] | .94257158 .86959489 1.0216725

Teruel M [47] | .93162383 .86054557 1.0085729

Teruel M [47] | .93597987 .86294529 1.0151957

Joo YB [48] | .93008813 .8595311 1.006437

Zhu Q [49] | .94930894 .87899356 1.0252492

Wu C [50] | .92998481 .86107019 1.0044149

Park JH [51] | .93376328 .8624081 1.0110223

Hsieh YY [52] | .93515361 .86439895 1.0116998

Du J [53] | .94737938 .87633168 1.0241872

Liu R [54] | .93649295 .86426728 1.0147544

García BM [55] | .93922276 .86451404 1.0203876

Chen F [56] | .9426882 .87000707 1.0214412

Inal EE [57] | .94063967 .86811917 1.0192184

Jacobson E [32] | .93981252 .8677722 1.0178334

Tanizawa K [58] | .93496115 .86361305 1.0122037

Chen F [59] | .93791907 .86583941 1.0159992

Chen F [56] | .93719264 .86449639 1.016002

Pu T [60] | .93855701 .86683598 1.0162122

Rodríguez [61] | .93848192 .86561083 1.0174876

Wei Y [62] | .95489907 .88634015 1.0287611

-------------------+----------------------------------------------------------

Combined | .93976624 .8687986 1.0165309

4

------------------------------------------------------------------------------

Study ommited | e^coef. [95% Conf. Interval]

-------------------+----------------------------------------------------------

Tomer Y [23] | .9354994 .8587384 1.0191219

Kim TY [24] | .94010217 .86411182 1.0227752

Heward JM [25] | .93270623 .85446469 1.0181122

Houston F [26] | .92736421 .84950986 1.0123536

Mukai T [27] | .92953283 .85207346 1.0140338

Kurylowicz A[28] | .93224979 .85419915 1.0174321

Meng F [30] | .9401515 .86414189 1.0228469

Ban Y [31] | .92729763 .8501209 1.0114807

Jacobson E [32] | .9413581 .86539631 1.0239876

Sun L [33] | .93187229 .85418801 1.0166216

Makni K [34] | .92605419 .85039718 1.0084421

Hsiao JY [35] | .92729101 .85033285 1.0112142

Su Y [36] | .94390885 .86945001 1.0247443

Yang J [38] | .93504524 .8578457 1.0191921

Inoue N [39] | .93197953 .85537419 1.0154455

Chen X [41] | .93841452 .86185795 1.0217714

Kim TY [24] | .926398 .84971412 1.0100023

Ban Y [31] | .92423604 .84775739 1.007614

Yang J [38] | .92602032 .84904178 1.0099781

Inoue N [39] | .92656754 .85038492 1.0095751

Buck D [42] | .92414062 .84745001 1.0077714

Sokolova EA[44] | .91975219 .84418616 1.0020824

**Wagner M [45] | .9176549 .84352212 .99830284**

Field J [46] | .92860794 .85220779 1.0118573

Teruel M [47] | .92634877 .84697311 1.0131633

Teruel M [47] | .92807464 .85011729 1.0131808

Teruel M [47] | .92285806 .84600459 1.0066931

Teruel M [47] | .92495919 .84700252 1.0100909

Joo YB [48] | .92025054 .84419567 1.0031573

Zhu Q [49] | .93821997 .86213633 1.021018

**Wu C [50] | .91732995 .84392201 .99712324**

Park JH [51] | .92350798 .84695695 1.006978

Hsieh YY [52] | .92492579 .8488636 1.0078035

Du J [53] | .93640371 .85992389 1.0196855

Liu R [54] | .9251617 .84808973 1.0092378

García BM [55] | .92925647 .84959128 1.0163918

Chen F [56] | .92626327 .84877524 1.0108255

Inal EE [57] | .93013213 .85265482 1.0146495

Jacobson E [32] | .92905977 .85207894 1.0129954

Tanizawa K [58] | .92408497 .84773836 1.0073073

Chen F [59] | .92596862 .84909658 1.0098002

Chen F [56] | .92522737 .84775994 1.0097737

Pu T [60] | .92836677 .8516588 1.0119838

Rodríguez [61] | .92494341 .84746696 1.0095029

Wei Y [62] | .94855451 .87536349 1.0278652

-------------------+----------------------------------------------------------

Combined | .92921762 .85334606 1.011835

5

------------------------------------------------------------------------------

Study ommited | e^coef. [95% Conf. Interval]

-------------------+----------------------------------------------------------

Tomer Y [23] | .95286728 .8463948 1.0727335

Kim TY [24] | .96261382 .85576343 1.0828055

Heward JM [25] | .94687608 .83736319 1.0707114

Houston F [26] | .94046598 .83296624 1.0618393

Mukai T [27] | .9493242 .83981609 1.0731117

Kurylowicz A[28] | .94712029 .83806777 1.0703631

Meng F [30] | .95175022 .84250028 1.075167

Ban Y [31] | .96234233 .85455034 1.0837311

Jacobson E [32] | .96111975 .85429549 1.0813017

Sun L [33] | .95533739 .84580078 1.0790597

Makni K [34] | .94580622 .8388873 1.0663523

Hsiao JY [35] | .94408566 .83601316 1.0661288

Su Y [36] | .96184305 .85436892 1.0828367

Yang J [38] | .95867639 .85074783 1.0802971

Inoue N [39] | .95782966 .85028965 1.0789708

Chen X [41] | .96492481 .85740595 1.0859266

Kim TY [24] | .94438904 .83605619 1.0667592

Ban Y [31] | .94925541 .84022238 1.0724373

Yang J [38] | .93791774 .83134032 1.0581583

Inoue N [39] | .94706698 .8389514 1.0691154

Buck D [42] | .93822227 .83157505 1.0585467

Sokolova EA[44] | .93674916 .8292186 1.058224

Wagner M [45] | .93292093 .82936798 1.0494033

Field J [46] | .94780165 .8402626 1.0691038

Teruel M [47] | .94263079 .83215192 1.0677772

Teruel M [47] | .93255743 .82849455 1.0496911

Teruel M [47] | .95465081 .84598668 1.0772725

Teruel M [47] | .94244308 .83317683 1.066039

Joo YB [48] | .94220027 .83235592 1.0665406

Zhu Q [49] | .94541147 .83711582 1.0677171

Wu C [50] | .93465138 .8289202 1.0538689

Park JH [51] | .94696661 .83808554 1.0699931

Hsieh YY [52] | .95057752 .84217852 1.0729288

Du J [53] | .94565399 .83723441 1.0681136

Liu R [54] | .94185752 .83339122 1.0644408

García BM [55] | .95463374 .8443551 1.0793155

Chen F [56] | .93011484 .82772698 1.0451678

Inal EE [57] | .94841478 .83952899 1.0714229

Jacobson E [32] | .94654078 .8385445 1.0684459

Tanizawa K [58] | .94497079 .83655592 1.0674359

Chen F [59] | .94036103 .8324614 1.0622461

Chen F [56] | .93953779 .83075087 1.0625704

Pu T [60] | .9486103 .84022977 1.0709707

Rodríguez [61] | .93437114 .82862288 1.0536149

Wei Y [62] | .9653017 .85824889 1.0857076

-------------------+----------------------------------------------------------

Combined | .94731274 .84063221 1.0675316

Overall-PB-sub

1

------------------------------------------------------------------------------

Study ommited | e^coef. [95% Conf. Interval]

-------------------+----------------------------------------------------------

Tomer Y [23] | .92554967 .8660624 .98912294

Kim TY [24] | .92984638 .87097236 .99270004

Heward JM [25] | .92168387 .86114069 .98648358

Houston F [26] | .91769146 .85746633 .98214658

Mukai T [27] | .92043459 .86023933 .98484202

Kurylowicz A[28] | .92155103 .86115768 .9861798

Luo H [29] | .92906225 .87007879 .99204425

Meng F [30] | .92606915 .8664405 .98980146

Ban Y [31] | .92203422 .86197039 .98628341

Jacobson E [32] | .93025191 .87142036 .99305531

Sun L [33] | .92287241 .86267182 .98727403

Makni K [34] | .91828341 .85940986 .98119008

Hsiao JY [35] | .918177 .85832759 .98219959

Su Y [36] | .93238284 .87418722 .9944526

Ma L [37] | .93127839 .87271618 .99377032

Yang J [38] | .92503288 .86522646 .98897326

Inoue N [39] | .9246198 .86515664 .98816993

Huang J [40] | .92952267 .87056974 .99246775

Kim TY [24] | .91785014 .85812274 .98173471

Ban Y [31] | .91790434 .85804358 .98194124

Yang J [38] | .91617869 .85651138 .98000261

Inoue N [39] | .91878662 .85930603 .98238442

Buck D [42] | .91527906 .85581953 .97886965

Blanco KF [43] | .91382766 .8532232 .97873685

Wagner M [45] | .91107094 .85327719 .97277913

Field J [46] | .92023929 .8608262 .98375299

Teruel M [47] | .91721294 .85593199 .98288134

Teruel M [47] | .91635652 .85626957 .98065995

Teruel M [47] | .91720684 .85711943 .98150661

Teruel M [47] | .91657661 .85612428 .98129757

Joo YB [48] | .9142318 .85430618 .97836092

Zhu Q [49] | .92398751 .86428872 .98780986

Park JH [51] | .91686771 .85704447 .98086673

Hsieh YY [52] | .91871795 .85912835 .98244072

Du J [53] | .92298624 .86317434 .98694268

García BM [55] | .92063175 .85943585 .9861851

Chen F [56] | .91370556 .85435514 .97717894

Inal EE [57] | .92056572 .86044586 .98488618

Jacobson E [32] | .91987915 .86010682 .9838053

Blanco KF [43] | .91309743 .8538675 .97643594

Blanco KF [43] | .91569699 .85450427 .98127183

Tanizawa K [58] | .9168303 .85717622 .98063594

Chen F [59] | .91660148 .85686271 .98050513

Chen F [56] | .91600927 .85584648 .98040126

Pu T [60] | .92006444 .86035098 .98392238

Rodríguez [61] | .91499977 .85522959 .97894717

Wei Y [62] | .93551307 .87811198 .99666641

-------------------+----------------------------------------------------------

Combined | .92054297 .8614804 .98365484

2

------------------------------------------------------------------------------

Study ommited | e^coef. [95% Conf. Interval]

-------------------+----------------------------------------------------------

Tomer Y [23] | .85816761 .74567485 .98763108

Kim TY [24] | .86904884 .75600528 .99899553

Heward JM [25] | .84992187 .73521535 .98252463

Houston F [26] | .84332008 .7305203 .97353731

Mukai T [27] | .85123294 .7367353 .9835249

Kurylowicz A[28] | .85024167 .73593526 .98230231

Luo H [29] | .86449223 .75068289 .99555595

Meng F [30] | .86168208 .7475228 .99327539

Ban Y [31] | .85952322 .74521077 .99137075

Jacobson E [32] | .86706743 .75390435 .9972166

Sun L [33] | .85649054 .74164456 .98912076

Makni K [34] | .84934647 .73715213 .97861675

Hsiao JY [35] | .84665512 .73350434 .9772606

**Su Y [36] | .87197213 .75927111 1.0014017**

**Ma L [37] | .88006629 .76945453 1.0065789**

Yang J [38] | .86278224 .74891791 .99395833

Inoue N [39] | .86040588 .74685076 .99122651

Huang J [40] | .86519453 .75134366 .99629718

Kim TY [24] | .84614952 .73297645 .97679674

Ban Y [31] | .84795057 .73422775 .97928767

Yang J [38] | .84108118 .72907474 .97029496

Inoue N [39] | .84772156 .73489895 .97786482

Buck D [42] | .84052493 .72869412 .96951813

Blanco KF [43] | .83976725 .72479876 .97297219

Wagner M [45] | .83426319 .72562883 .95916128

Field J [46] | .85124795 .73838433 .98136301

Teruel M [47] | .84414537 .72876026 .97779948

Teruel M [47] | .83698686 .72629235 .96455237

Teruel M [47] | .85427312 .74004814 .9861285

Teruel M [47] | .84384777 .72975696 .97577564

Joo YB [48] | .84119104 .72742739 .97274638

Zhu Q [49] | .85556898 .74161018 .98703914

Park JH [51] | .84662528 .73303149 .97782206

Hsieh YY [52] | .84917674 .73591531 .97986974

Du J [53] | .85456262 .74058724 .98607866

García BM [55] | .85407263 .73829452 .98800686

Chen F [56] | .83680224 .72575372 .96484244

Inal EE [57] | .85122609 .73712696 .9829865

Jacobson E [32] | .84965325 .73637886 .9803522

Blanco KF [43] | .83793864 .72534221 .96801365

Blanco KF [43] | .84766745 .73175284 .98194372

Tanizawa K [58] | .84515752 .73214934 .97560865

Chen F [59] | .84306611 .73016592 .97342324

Chen F [56] | .84196117 .72832252 .97333062

Pu T [60] | .85071718 .73720996 .98170096

Rodríguez [61] | .83805952 .72645242 .96681316

**Wei Y [62] | .87701034 .76511042 1.005276**

-------------------+----------------------------------------------------------

Combined | .85138805 .73923488 .98055656

3

------------------------------------------------------------------------------

Study ommited | e^coef. [95% Conf. Interval]

-------------------+----------------------------------------------------------

Tomer Y [23] | .93169171 .86148874 1.0076155

Kim TY [24] | .93454149 .86469602 1.0100287

Heward JM [25] | .93130075 .8601923 1.0082874

Houston F [26] | .92589887 .85475055 1.0029695

Mukai T [27] | .92644973 .85574917 1.0029915

Kurylowicz A[28] | .93046765 .85938969 1.0074243

Luo H [29] | .93477458 .86527353 1.0098581

Meng F [30] | .93759299 .86828009 1.012439

**Ban Y [31] | .92219882 .85209891 .99806566**

Jacobson E [32] | .93679273 .86703391 1.0121641

Sun L [33] | .92750426 .85658225 1.0042984

**Makni K [34] | .92482091 .85538965 .99988785**

Hsiao JY [35] | .92539231 .85500089 1.001579

Su Y [36] | .93880753 .87023856 1.0127793

Ma L [37] | .93260125 .8628471 1.0079944

Yang J [38] | .93104331 .86045195 1.007426

Inoue N [39] | .92737075 .85732823 1.0031357

Huang J [40] | .93649539 .86721442 1.0113112

Kim TY [24] | .92457971 .85443122 1.0004874

**Ban Y [31] | .92154588 .8518106 .99699019**

Yang J [38] | .92538484 .85484394 1.0017467

**Inoue N [39] | .92458155 .85491866 .99992091**

**Buck D [42] | .92362962 .85322507 .99984366**

**Blanco KF [43] | .91783535 .84707093 .99451144**

**Wagner M [45] | .91791755 .84896918 .99246551**

Field J [46] | .92617736 .85631326 1.0017415

Teruel M [47] | .92390245 .85168041 1.0022489

Teruel M [47] | .9292003 .85812753 1.0061595

**Teruel M [47] | .91910943 .84930014 .99465678**

**Teruel M [47] | .92265428 .85140523 .99986574**

**Joo YB [48] | .9177555 .84812658 .99310077**

Zhu Q [49] | .93697021 .86791867 1.0115155

**Park JH [51] | .92109596 .85119927 .99673226**

**Hsieh YY [52] | .92262813 .85328663 .9976046**

Du J [53] | .93473469 .86513716 1.0099311

García BM [55] | .92527233 .85284707 1.0038481

Chen F [56] | .92941894 .85856793 1.0061167

Inal EE [57] | .92745808 .85672121 1.0040355

Jacobson E [32] | .92681826 .8564736 1.0029405

**Blanco KF [43] | .91733637 .84779592 .99258086**

**Blanco KF [43] | .91803334 .84745072 .99449466**

**Tanizawa K [58] | .92226241 .85242001 .9978273**

Chen F [59] | .92493169 .85453531 1.0011273

Chen F [56] | .92399482 .85306843 1.0008182

Pu T [60] | .92568519 .85560481 1.0015057

Rodríguez [61] | .92520153 .85415852 1.0021534

Wei Y [62] | .9432656 .87551087 1.0162638

-------------------+----------------------------------------------------------

Combined | .92716571 .85771392 1.0022412

4

------------------------------------------------------------------------------

Study ommited | e^coef. [95% Conf. Interval]

-------------------+----------------------------------------------------------

Tomer Y [23] | .91351312 .84057246 .99278319

Kim TY [24] | .91822207 .84585534 .99678011

Heward JM [25] | .91035511 .83635214 .99090608

Houston F [26] | .90520459 .83141147 .98554733

Mukai T [27] | .90748198 .83400857 .98742815

Kurylowicz A[28] | .90993428 .83604152 .99035797

Luo H [29] | .91803085 .84579033 .99644156

Meng F [30] | .918262 .84588113 .99683641

Ban Y [31] | .90542069 .83216364 .98512671

Jacobson E [32] | .91944395 .84711047 .99795388

Sun L [33] | .90967023 .83603205 .98979451

Makni K [34] | .90488013 .83284966 .98314029

Hsiao JY [35] | .9055135 .83243357 .98500918

Su Y [36] | .92247488 .85125023 .99965894

Ma L [37] | .91658351 .84436087 .99498373

Yang J [38] | .91291231 .83964242 .99257596

Inoue N [39] | .91021505 .83741758 .98934086

Huang J [40] | .91950594 .84742376 .99771945

Kim TY [24] | .90476348 .83190237 .98400604

Ban Y [31] | .9027104 .8299933 .98179837

Yang J [38] | .90425203 .83114593 .98378841

Inoue N [39] | .90516844 .83272078 .98391913

Buck D [42] | .90251298 .82961763 .98181337

Blanco KF [43] | .89858781 .82484991 .97891755

Wagner M [45] | .89698174 .82594352 .97412985

Field J [46] | .90706953 .83445275 .98600565

Teruel M [47] | .90366005 .82868538 .98541798

Teruel M [47] | .90586484 .83199905 .98628852

Teruel M [47] | .9011282 .82805757 .98064682

Teruel M [47] | .90276976 .82885367 .98327759

Joo YB [48] | .89869105 .82608086 .97768347

Zhu Q [49] | .91638488 .84395214 .99503422

Park JH [51] | .90194189 .8291496 .98112472

Hsieh YY [52] | .90359465 .83123055 .98225853

Du J [53] | .91449096 .84176304 .99350253

García BM [55] | .90655148 .83148474 .98839527

Chen F [56] | .90426633 .83075041 .98428792

Inal EE [57] | .90805701 .83457082 .98801386

Jacobson E [32] | .90723002 .83413698 .98672799

Blanco KF [43] | .8980802 .82586802 .97660645

Blanco KF [43] | .89963682 .82549619 .98043628

Tanizawa K [58] | .90262186 .83001195 .98158373

Chen F [59] | .90425032 .83123092 .98368409

Chen F [56] | .90324522 .82973037 .98327355

Pu T [60] | .90668396 .83381258 .98592396

Rodríguez [61] | .90295699 .82942912 .983003

**Wei Y [62] | .92742468 .85714596 1.0034657**

-------------------+----------------------------------------------------------

Combined | .90788072 .83568459 .98631399

5

------------------------------------------------------------------------------

Study ommited | e^coef. [95% Conf. Interval]

-------------------+----------------------------------------------------------

Tomer Y [23] | .90966633 .80995799 1.0216491

Kim TY [24] | .91804111 .81783417 1.0305261

Heward JM [25] | .90285646 .80082843 1.0178832

Houston F [26] | .89754063 .79721375 1.0104933

Mukai T [27] | .90510561 .80303615 1.0201486

Kurylowicz A[28] | .90327974 .8016168 1.0178358

Luo H [29] | .91300914 .81167596 1.0269932

Meng F [30] | .90742269 .805517 1.0222204

Ban Y [31] | .91725315 .81628535 1.0307098

Jacobson E [32] | .91679642 .81664531 1.0292298

Sun L [33] | .91037002 .80813077 1.0255439

Makni K [34] | .90313836 .80319791 1.0155142

Hsiao JY [35] | .9008524 .80001706 1.0143972

Su Y [36] | .91707719 .81638838 1.0301844

Ma L [37] | .93234289 .83583461 1.0399943

Yang J [38] | .91419682 .8132191 1.027713

Inoue N [39] | .91367492 .81302745 1.0267819

Huang J [40] | .9125113 .81091474 1.0268365

Kim TY [24] | .90102125 .79997503 1.0148308

Ban Y [31] | .90527076 .80358717 1.0198211

Yang J [38] | .8953507 .79578919 1.0073684

Inoue N [39] | .90371952 .80280563 1.0173184

Buck D [42] | .89563107 .79600971 1.0077201

Blanco KF [43] | .89592193 .79291555 1.0123097

Wagner M [45] | .89122434 .79411154 1.0002132

Field J [46] | .90477721 .80431435 1.0177884

Teruel M [47] | .89865084 .7959346 1.0146227

Teruel M [47] | .89073855 .79323607 1.0002258

Teruel M [47] | .91032207 .80883627 1.0245414

Teruel M [47] | .89879803 .79704892 1.0135362

Joo YB [48] | .89840647 .7962055 1.0137259

Zhu Q [49] | .90202746 .80099495 1.0158036

Park JH [51] | .90321638 .8016931 1.0175962

Hsieh YY [52] | .90683784 .80564166 1.0207452

Du J [53] | .90219772 .80106299 1.0161008

García BM [55] | .90932153 .80640655 1.0253707

**Chen F [56] | .88867671 .79254204 .99647244**

Inal EE [57] | .90456834 .80301738 1.0189616

Jacobson E [32] | .9032826 .80247171 1.0167579

Blanco KF [43] | .89374112 .79257704 1.0078177

Blanco KF [43] | .90554335 .80229845 1.0220745

Tanizawa K [58] | .90154327 .80042293 1.0154385

Chen F [59] | .89727671 .79663878 1.010628

Chen F [56] | .89618228 .79487147 1.0104057

Pu T [60] | .90502753 .80387818 1.0189042

Rodríguez [61] | .89204753 .79320609 1.0032056

Wei Y [62] | .92004408 .81959253 1.0328072

-------------------+----------------------------------------------------------

Combined | .90460242 .80482723 1.0167468

Overall-欧美-sub

1

------------------------------------------------------------------------------

Study ommited | e^coef. [95% Conf. Interval]

-------------------+----------------------------------------------------------

Tomer Y [23] | 1.0298042 .95928626 1.1055059

Heward JM [25] | 1.0298354 .95684858 1.1083896

Houston F [26] | 1.0187648 .94461032 1.0987405

Kurylowicz A[28] | 1.028212 .95498058 1.1070592

Jacobson E [32] | 1.0398806 .97387847 1.1103558

Makni K [34] | 1.0162524 .94561423 1.0921674

Buck D [42] | 1.0126785 .94029582 1.0906331

Blanco KF [43] | 1.010781 .93482859 1.0929044

Sokolova EA[44] | 1.0071183 .93502692 1.0847679

Wagner M [45] | 1.0036297 .93611633 1.0760121

Field J [46] | 1.0194648 .94751842 1.0968741

Teruel M [47] | 1.0203541 .94407836 1.1027924

Teruel M [47] | 1.0157732 .94174781 1.0956172

Teruel M [47] | 1.0174238 .94353105 1.0971034

Teruel M [47] | 1.0170255 .94211767 1.0978892

García BM [55] | 1.0305843 .9578907 1.1087946

Inal EE [57] | 1.0239248 .95060443 1.1029003

Jacobson E [32] | 1.0207009 .94797647 1.0990045

Blanco KF [43] | 1.0071258 .9348219 1.0850222

Blanco KF [43] | 1.0163356 .93960531 1.0993319

Rodríguez [61] | 1.0124996 .93915886 1.0915677

-------------------+----------------------------------------------------------

Combined | 1.0190095 .94796446 1.0953791

2

------------------------------------------------------------------------------

Study ommited | e^coef. [95% Conf. Interval]

-------------------+----------------------------------------------------------

Tomer Y [23] | 1.0843679 .932275 1.2612734

Heward JM [25] | 1.0866973 .92353648 1.2786836

Houston F [26] | 1.0645306 .90565596 1.2512759

Kurylowicz A[28] | 1.0842838 .92232978 1.2746758

Jacobson E [32] | 1.106625 .95921343 1.2766908

Makni K [34] | 1.0700875 .91445717 1.2522044

Buck D [42] | 1.0570758 .90129561 1.2397811

Blanco KF [43] | 1.0618142 .89382688 1.2613733

Sokolova EA[44] | 1.0510558 .89339774 1.2365357

Wagner M [45] | 1.0417553 .89820714 1.2082447

Field J [46] | 1.074846 .91760936 1.2590258

Teruel M [47] | 1.0754445 .90812353 1.2735941

Teruel M [47] | 1.047619 .89569967 1.2253054

Teruel M [47] | 1.0908343 .93089747 1.2782498

Teruel M [47] | 1.0701785 .90662004 1.2632436

García BM [55] | 1.1108361 .95296626 1.2948589

Inal EE [57] | 1.0842884 .92335181 1.2732757

Jacobson E [32] | 1.0757188 .91686711 1.2620923

Blanco KF [43] | 1.0510561 .89083533 1.2400933

Blanco KF [43] | 1.0878534 .9215174 1.2842135

Rodríguez [61] | 1.0506295 .89505987 1.2332385

-------------------+----------------------------------------------------------

Combined | 1.0727184 .91802006 1.2534855

3

------------------------------------------------------------------------------

Study ommited | e^coef. [95% Conf. Interval]

-------------------+----------------------------------------------------------

Tomer Y [23] | 1.0185052 .93946592 1.1041942

Heward JM [25] | 1.026113 .94674667 1.1121328

Houston F [26] | 1.0128471 .93069091 1.1022556

Kurylowicz A[28] | 1.0225067 .94218368 1.1096774

Jacobson E [32] | 1.0278935 .95209981 1.1097209

Makni K [34] | 1.0071634 .92826296 1.0927703

Buck D [42] | 1.0071149 .926103 1.0952134

Blanco KF [43] | .99693106 .91454874 1.0867344

Sokolova EA[44] | .99577657 .91571394 1.0828392

Wagner M [45] | .99739928 .91979711 1.0815486

Field J [46] | 1.0089955 .9291385 1.095716

Teruel M [47] | 1.0124677 .92825238 1.1043233

Teruel M [47] | 1.0190705 .93803453 1.1071072

Teruel M [47] | .99947378 .91964511 1.0862319

Teruel M [47] | 1.0073566 .92441485 1.0977403

García BM [55] | 1.0167807 .93332369 1.1077005

Inal EE [57] | 1.0137637 .93265068 1.1019312

Jacobson E [32] | 1.0112187 .93065609 1.0987553

Blanco KF [43] | .99445105 .91521436 1.0805478

Blanco KF [43] | .9972942 .91539028 1.0865264

Rodríguez [61] | 1.0112406 .92913317 1.1006039

-------------------+----------------------------------------------------------

Combined | 1.0097721 .93101872 1.0951871

4

------------------------------------------------------------------------------

Study ommited | e^coef. [95% Conf. Interval]

-------------------+----------------------------------------------------------

Tomer Y [23] | 1.026976 .94376527 1.1175232

Heward JM [25] | 1.0307524 .94602558 1.1230674

Houston F [26] | 1.017209 .93022557 1.1123261

Kurylowicz A[28] | 1.0280059 .94257759 1.1211767

Jacobson E [32] | 1.0384549 .95980382 1.1235511

Makni K [34] | 1.0121627 .92906469 1.1026932

Buck D [42] | 1.0103454 .92508497 1.1034639

Blanco KF [43] | 1.0036045 .91589498 1.0997134

Sokolova EA[44] | 1.0008411 .91651785 1.0929223

Wagner M [45] | .99936954 .91903775 1.086723

Field J [46] | 1.0153235 .93093263 1.1073645

Teruel M [47] | 1.0177448 .92855491 1.1155017

Teruel M [47] | 1.0187343 .93175459 1.1138335

Teruel M [47] | 1.0082586 .92243515 1.1020672

Teruel M [47] | 1.0129684 .92529992 1.1089431

García BM [55] | 1.0261944 .93935482 1.121062

Inal EE [57] | 1.020761 .93496017 1.1144357

Jacobson E [32] | 1.017424 .93220416 1.1104344

Blanco KF [43] | .99996726 .91629889 1.0912755

Blanco KF [43] | 1.0067729 .91817707 1.1039174

Rodríguez [61] | 1.0123877 .92562707 1.1072805

-------------------+----------------------------------------------------------

Combined | 1.0154809 .93217615 1.1062303

5

------------------------------------------------------------------------------

Study ommited | e^coef. [95% Conf. Interval]

-------------------+----------------------------------------------------------

Tomer Y [23] | 1.064893 .97409146 1.1641587

Heward JM [25] | 1.067414 .97454728 1.1691301

Houston F [26] | 1.056403 .96542946 1.155949

Kurylowicz A[28] | 1.0656581 .97355011 1.1664804

Jacobson E [32] | 1.0739376 .98196892 1.1745198

Makni K [34] | 1.0605844 .97019063 1.1594003

Buck D [42] | 1.0535443 .96288601 1.1527382

Blanco KF [43] | 1.0532018 .95548334 1.160914

Sokolova EA[44] | 1.045663 .95360748 1.1466049

Wagner M [45] | 1.0462087 .95626383 1.1446138

Field J [46] | 1.0622597 .97162183 1.1613527

Teruel M [47] | 1.0637621 .96851331 1.1683781

Teruel M [47] | 1.0435186 .9533856 1.1421727

Teruel M [47] | 1.0722711 .97999714 1.1732333

Teruel M [47] | 1.0602423 .96741821 1.1619728

**García BM [55] | 1.1025248 1.0036062 1.2111932**

Inal EE [57] | 1.0662423 .97435776 1.1667918

Jacobson E [32] | 1.0622724 .97132259 1.1617382

Blanco KF [43] | 1.0370145 .94217518 1.1414003

Blanco KF [43] | 1.0890655 .99154948 1.1961719

Rodríguez [61] | 1.0436716 .95284503 1.1431559

-------------------+----------------------------------------------------------

Combined | 1.0613263 .97090238 1.1601717

Overall-欧美- HWE-sub

1

------------------------------------------------------------------------------

Study ommited | e^coef. [95% Conf. Interval]

-------------------+----------------------------------------------------------

Tomer Y [23] | 1.0068386 .92299818 1.0982946

Heward JM [25] | 1.0041955 .91555528 1.1014176

Houston F [26] | .99170248 .90318683 1.088893

Kurylowicz A[28] | 1.0028479 .91443317 1.0998112

Jacobson E [32] | 1.0180494 .93990531 1.1026903

Makni K [34] | .98933165 .90645357 1.0797874

Buck D [42] | .98407237 .89887468 1.0773453

Sokolova EA[44] | .97678837 .89475779 1.0663394

Wagner M [45] | .97167925 .8953875 1.0544715

Field J [46] | .99394398 .9088536 1.0870008

Teruel M [47] | .99313244 .90056269 1.0952175

Teruel M [47] | .98795145 .9000104 1.0844853

Teruel M [47] | .9900871 .90213554 1.0866133

Teruel M [47] | .9894059 .89994413 1.0877609

García BM [55] | 1.003649 .91372019 1.1024287

Inal EE [57] | .99846061 .91072655 1.0946464

Jacobson E [32] | .99498851 .90855068 1.0896499

Rodríguez [61] | .98380397 .89735405 1.0785824

-------------------+----------------------------------------------------------

Combined | .9934532 .90980056 1.0847974

2

------------------------------------------------------------------------------

Study ommited | e^coef. [95% Conf. Interval]

-------------------+----------------------------------------------------------

Tomer Y [23] | 1.0697315 .88000314 1.3003652

Heward JM [25] | 1.0671402 .86036111 1.3236165

Houston F [26] | 1.0387383 .84056049 1.2836402

Kurylowicz A[28] | 1.0651074 .8605302 1.3183195

Jacobson E [32] | 1.1015373 .91537796 1.3255556

Makni K [34] | 1.0471759 .85438051 1.2834765

Buck D [42] | 1.0284084 .83536881 1.2660562

Sokolova EA[44] | 1.0226012 .82703241 1.2644163

Wagner M [45] | 1.0065913 .83252655 1.2170496

Field J [46] | 1.0545683 .85869621 1.2951197

Teruel M [47] | 1.0511241 .83852343 1.3176279

Teruel M [47] | 1.0161568 .82928256 1.2451422

Teruel M [47] | 1.0753952 .8731733 1.3244504

Teruel M [47] | 1.0457617 .83978632 1.3022569

García BM [55] | 1.0940125 .89135337 1.3427484

Inal EE [57] | 1.0659535 .86297195 1.3166787

Jacobson E [32] | 1.0550843 .85633607 1.2999603

Rodríguez [61] | 1.0211403 .82860256 1.2584169

-------------------+----------------------------------------------------------

Combined | 1.051466 .85974762 1.2859364

3

------------------------------------------------------------------------------

Study ommited | e^coef. [95% Conf. Interval]

-------------------+----------------------------------------------------------

Tomer Y [23] | .97252341 .91416911 1.0346027

Heward JM [25] | .98363266 .92240272 1.0489271

Houston F [26] | .96911814 .90991789 1.03217

Kurylowicz A[28] | .97836687 .91819599 1.0424808

Jacobson E [32] | .97890359 .91986021 1.0417368

Makni K [34] | .96601591 .90841118 1.0272735

Buck D [42] | .96500779 .90683776 1.0269092

Sokolova EA[44] | .93647493 .87698505 1.0000003

Wagner M [45] | .95415452 .89630422 1.0157387

Field J [46] | .96723317 .90952658 1.028601

Teruel M [47] | .96875971 .90669097 1.0350774

Teruel M [47] | .97464056 .91507256 1.0380862

Teruel M [47] | .95566293 .89743531 1.0176685

Teruel M [47] | .9627568 .90329636 1.0261313

García BM [55] | .97702873 .91337136 1.0451227

Inal EE [57] | .96983165 .91127528 1.0321507

Jacobson E [32] | .96823927 .91018133 1.0300006

Rodríguez [61] | .96769574 .90872429 1.0304941

-------------------+----------------------------------------------------------

Combined | .96753551 .90990783 1.028813

4

------------------------------------------------------------------------------

Study ommited | e^coef. [95% Conf. Interval]

-------------------+----------------------------------------------------------

Tomer Y [23] | .9871708 .89532401 1.0884397

Heward JM [25] | .98895308 .89296074 1.0952645

Houston F [26] | .97452987 .87771869 1.0820192

Kurylowicz A[28] | .98659396 .89044248 1.093128

Jacobson E [32] | .99856894 .91270939 1.0925054

Makni K [34] | .9702119 .87960854 1.0701478

Buck D [42] | .96676046 .8733708 1.0701363

Sokolova EA[44] | .9520464 .86621908 1.0463777

Wagner M [45] | .95207221 .86892574 1.0431749

Field J [46] | .97429635 .88145407 1.0769176

Teruel M [47] | .97434806 .87356008 1.0867646

Teruel M [47] | .97629209 .87925468 1.0840389

Teruel M [47] | .96358148 .86997474 1.06726

Teruel M [47] | .96911049 .87203206 1.0769961

García BM [55] | .98283498 .88280654 1.0941974

Inal EE [57] | .97942817 .88404516 1.0851024

Jacobson E [32] | .97611141 .88192944 1.0803512

Rodríguez [61] | .96869662 .87297596 1.0749129

-------------------+----------------------------------------------------------

Combined | .97462244 .88341543 1.075246

5

------------------------------------------------------------------------------

Study ommited | e^coef. [95% Conf. Interval]

-------------------+----------------------------------------------------------

Tomer Y [23] | 1.0839519 .90800724 1.2939894

Heward JM [25] | 1.0803589 .88755795 1.3150413

Houston F [26] | 1.0568029 .87238255 1.2802095

Kurylowicz A[28] | 1.0790862 .88857853 1.3104379

Jacobson E [32] | 1.1086988 .93499682 1.3146709

Makni K [34] | 1.066138 .88602921 1.2828586

Buck D [42] | 1.0493887 .86872293 1.267627

Sokolova EA[44] | 1.0483079 .86161594 1.2754517

Wagner M [45] | 1.0321792 .86602607 1.2302099

Field J [46] | 1.0729649 .89024959 1.2931808

Teruel M [47] | 1.0704876 .8717203 1.3145774

Teruel M [47] | 1.0312572 .86219701 1.2334669

Teruel M [47] | 1.0990705 .91289323 1.3232172

Teruel M [47] | 1.0672045 .87407068 1.303013

García BM [55] | 1.1139024 .92661453 1.3390451

Inal EE [57] | 1.0818011 .89248893 1.3112697

Jacobson E [32] | 1.0724171 .88713504 1.2963961

Rodríguez [61] | 1.0384258 .86067845 1.2528815

-------------------+----------------------------------------------------------

Combined | 1.0695309 .89090027 1.2839781

Overall-欧美- PB-sub

1

------------------------------------------------------------------------------

Study ommited | e^coef. [95% Conf. Interval]

-------------------+----------------------------------------------------------

Tomer Y [23] | 1.0180721 .94648645 1.0950719

Heward JM [25] | 1.0176659 .94314592 1.0980739

Houston F [26] | 1.0062451 .93077787 1.0878311

Kurylowicz A[28] | 1.0160769 .94139568 1.0966825

Jacobson E [32] | 1.0282971 .96160919 1.0996099

Makni K [34] | 1.0041988 .93259219 1.0813035

Buck D [42] | 1.0000102 .92660762 1.0792274

Blanco KF [43] | .99762979 .92095224 1.0806914

Wagner M [45] | .99048376 .92281516 1.0631144

Field J [46] | 1.0075064 .93442526 1.0863033

Teruel M [47] | 1.0076731 .92973367 1.0921463

Teruel M [47] | 1.0030781 .92783058 1.0844283

Teruel M [47] | 1.0048679 .92971909 1.086091

Teruel M [47] | 1.0043007 .92805482 1.0868107

García BM [55] | 1.0181405 .94360406 1.0985646

Inal EE [57] | 1.0117724 .93713245 1.0923572

Jacobson E [32] | 1.0085896 .9346568 1.0883706

Blanco KF [43] | .99362119 .92131311 1.0716043

Blanco KF [43] | 1.0034785 .92526558 1.0883029

Rodríguez [61] | .99962769 .92522957 1.0800082

-------------------+----------------------------------------------------------

Combined | 1.0071183 .93502692 1.0847679

2

------------------------------------------------------------------------------

Study ommited | e^coef. [95% Conf. Interval]

-------------------+----------------------------------------------------------

Tomer Y [23] | 1.0632049 .9081864 1.2446836

Heward JM [25] | 1.0643751 .89730155 1.2625569

Houston F [26] | 1.0414748 .87952523 1.2332446

Kurylowicz A[28] | 1.0621243 .89644647 1.258422

Jacobson E [32] | 1.0861184 .93576912 1.2606243

Makni K [34] | 1.0482301 .88960924 1.2351336

Buck D [42] | 1.03365 .87517463 1.2208218

Blanco KF [43] | 1.0378502 .86657815 1.2429727

Wagner M [45] | 1.0175457 .87266 1.1864864

Field J [46] | 1.0531659 .89274969 1.2424071

Teruel M [47] | 1.0521067 .88074729 1.256806

Teruel M [47] | 1.0234728 .86944519 1.2047873

Teruel M [47] | 1.0692873 .90566595 1.2624692

Teruel M [47] | 1.0469183 .87984275 1.2457201

García BM [55] | 1.0885011 .92592461 1.2796233

Inal EE [57] | 1.0623414 .89777396 1.2570752

Jacobson E [32] | 1.0537347 .89157533 1.2453875

Blanco KF [43] | 1.0264236 .86424261 1.219039

Blanco KF [43] | 1.0649206 .89412482 1.2683418

Rodríguez [61] | 1.0264402 .86850243 1.2130991

-------------------+----------------------------------------------------------

Combined | 1.0510558 .89339774 1.2365357

3

------------------------------------------------------------------------------

Study ommited | e^coef. [95% Conf. Interval]

-------------------+----------------------------------------------------------

Tomer Y [23] | 1.0045855 .92410004 1.092081

Heward JM [25] | 1.0118072 .93058966 1.1001131

Houston F [26] | .99825148 .91437562 1.0898213

Kurylowicz A[28] | 1.0082302 .92612444 1.0976152

Jacobson E [32] | 1.0142157 .93718813 1.0975742

Makni K [34] | .99299672 .91281297 1.080224

Buck D [42] | .99253254 .91005199 1.0824885

Blanco KF [43] | .98115615 .89826002 1.0717024

Wagner M [45] | .98230472 .90393562 1.0674682

Field J [46] | .99489042 .91361729 1.0833934

Teruel M [47] | .99739575 .91111338 1.0918491

Teruel M [47] | 1.004749 .92196636 1.0949645

Teruel M [47] | .98430269 .90339608 1.0724551

Teruel M [47] | .99235619 .9077573 1.0848393

García BM [55] | 1.0017129 .91600099 1.0954451

Inal EE [57] | .99945665 .91673576 1.0896418

Jacobson E [32] | .99700381 .9149344 1.0864348

Blanco KF [43] | .97852006 .89926351 1.0647619

Blanco KF [43] | .98156094 .89886847 1.0718608

Rodríguez [61] | .99661142 .91283242 1.0880796

-------------------+----------------------------------------------------------

Combined | .99577657 .91571394 1.0828392

4

------------------------------------------------------------------------------

Study ommited | e^coef. [95% Conf. Interval]

-------------------+----------------------------------------------------------

Tomer Y [23] | 1.0124309 .9281227 1.1043974

Heward JM [25] | 1.0157399 .92942559 1.11007

Houston F [26] | 1.0018842 .91351513 1.0988016

Kurylowicz A[28] | 1.0130397 .92610927 1.1081299

Jacobson E [32] | 1.0240977 .94471951 1.1101455

Makni K [34] | .99732591 .91327968 1.0891066

Buck D [42] | .99495017 .9086166 1.0894869

Blanco KF [43] | .98728355 .89930806 1.0838653

Wagner M [45] | .98341784 .90295429 1.0710516

Field J [46] | 1.0005825 .91506888 1.0940875

Teruel M [47] | 1.0020922 .91113665 1.1021274

Teruel M [47] | 1.0034623 .91505904 1.1004062

Teruel M [47] | .99253563 .90568137 1.0877192

Teruel M [47] | .99731126 .9083018 1.0950433

García BM [55] | 1.0106601 .92181874 1.1080636

Inal EE [57] | 1.0058058 .91869007 1.1011823

Jacobson E [32] | 1.0025338 .91610613 1.0971152

Blanco KF [43] | .98330793 .90010273 1.0742046

Blanco KF [43] | .99064919 .901061 1.0891447

Rodríguez [61] | .99683999 .90883913 1.0933618

-------------------+----------------------------------------------------------

Combined | 1.0008411 .91651785 1.0929223

5

------------------------------------------------------------------------------

Study ommited | e^coef. [95% Conf. Interval]

-------------------+----------------------------------------------------------

Tomer Y [23] | 1.0493989 .9569342 1.1507981

Heward JM [25] | 1.0513558 .95670044 1.1553763

Houston F [26] | 1.0400846 .94746487 1.1417585

Kurylowicz A[28] | 1.0497325 .95588532 1.1527934

Jacobson E [32] | 1.0588038 .96507889 1.161631

Makni K [34] | 1.0448674 .95284594 1.1457759

Buck D [42] | 1.0370937 .94481558 1.1383845

Blanco KF [43] | 1.0337008 .93395188 1.1441032

Wagner M [45] | 1.0293849 .93788214 1.129815

Field J [46] | 1.0465963 .95431225 1.1478044

Teruel M [47] | 1.0464101 .94924265 1.153524

Teruel M [47] | 1.0263694 .93467786 1.1270559

Teruel M [47] | 1.056877 .96283394 1.1601055

Teruel M [47] | 1.0435357 .94894892 1.1475505

García BM [55] | 1.0875581 .9863502 1.1991508

Inal EE [57] | 1.0504553 .95684249 1.1532267

Jacobson E [32] | 1.0464899 .9538767 1.148095

Blanco KF [43] | 1.0171152 .92048658 1.1238875

Blanco KF [43] | 1.0733009 .97363463 1.1831695

Rodríguez [61] | 1.0262412 .93381911 1.1278106

-------------------+----------------------------------------------------------

Combined | 1.045663 .95360748 1.1466049

Overall- Asian –sub

1

------------------------------------------------------------------------------

Study ommited | e^coef. [95% Conf. Interval]

-------------------+----------------------------------------------------------

Kim TY [24] | .87526482 .78638495 .97419019

Mukai T [27] | .8604849 .76943857 .9623046

Luo H [29] | .8746484 .78565419 .97372335

Meng F [30] | .86887909 .77856758 .96966646

Ban Y [31] | .86292014 .77205784 .96447588

Sun L [33] | .86352026 .77181983 .96611567

Hsiao JY [35] | .85723615 .76745247 .95752354

Su Y [36] | .87971656 .79216174 .97694849

Ma L [37] | .87710969 .78875702 .97535919

Yang J [38] | .86711385 .77643159 .96838723

Inoue N [39] | .86882461 .7793056 .96862669

Huang J [40] | .87466053 .78562314 .97378883

Chen X [41] | .87247416 .78285166 .97235684

Kim TY [24] | .85669534 .76725957 .95655622

Ban Y [31] | .8568092 .76701994 .95710941

Yang J [38] | .8541154 .76498735 .95362769

Inoue N [39] | .85837617 .76949879 .95751891

Joo YB [48] | .8519983 .76282334 .95159791

Zhu Q [49] | .86682371 .77677828 .96730736

Wu C [50] | .8460004 .76169595 .93963565

Park JH [51] | .85522969 .7656354 .95530826

Hsieh YY [52] | .85819777 .76904182 .95768968

Du J [53] | .8650249 .77481018 .96574373

Liu R [54] | .85496056 .76504471 .95544423

Chen F [56] | .85075393 .76285491 .94878101

Tanizawa K [58] | .85505454 .76589579 .95459237

Chen F [59] | .85478304 .76543207 .95456418

Chen F [56] | .85425867 .76386523 .95534898

Pu T [60] | .86054805 .7709465 .96056334

Wei Y [62] | .88270685 .79650781 .97823445

-------------------+----------------------------------------------------------

Combined | .86280281 .77492949 .96064054

2

------------------------------------------------------------------------------

Study ommited | e^coef. [95% Conf. Interval]

-------------------+----------------------------------------------------------

Kim TY [24] | .76862019 .62229189 .94935673

Mukai T [27] | .74291306 .59557304 .92670382

Luo H [29] | .76109543 .61383137 .94368954

Meng F [30] | .75669096 .60900929 .94018471

Ban Y [31] | .75382967 .60606942 .93761399

Sun L [33] | .74875775 .59984048 .93464542

Hsiao JY [35] | .73721096 .59304943 .91641603

Su Y [36] | .77166948 .62569835 .95169468

Ma L [37] | .78392481 .64095775 .958781

Yang J [38] | .7594245 .61224593 .9419835

Inoue N [39] | .75823887 .61180567 .93972028

Huang J [40] | .76099762 .61351688 .94393062

Chen X [41] | .76189041 .61428461 .94496427

Kim TY [24] | .73646075 .59230317 .91570409

Ban Y [31] | .738915 .59351821 .91993027

Yang J [38] | .72913468 .58768224 .90463408

Inoue N [39] | .73906302 .59542151 .91735711

Joo YB [48] | .73047533 .58584794 .91080666

Zhu Q [49] | .75001338 .60341733 .93222391

Wu C [50] | .71868542 .58435284 .88389873

Park JH [51] | .7370859 .59206052 .91763528

Hsieh YY [52] | .74113968 .59651725 .92082505

Du J [53] | .7484748 .60195375 .93066042

Liu R [54] | .73300062 .58890909 .91234778

Chen F [56] | .72422707 .58563443 .89561819

Tanizawa K [58] | .73503434 .59128518 .91373081

Chen F [59] | .73220268 .58886136 .91043633

Chen F [56] | .73124599 .58664801 .91148472

Pu T [60] | .74336711 .59812271 .92388176

Wei Y [62] | .77577615 .63075806 .95413547

-------------------+----------------------------------------------------------

Combined | .74686648 .60409968 .92337334

3

------------------------------------------------------------------------------

Study ommited | e^coef. [95% Conf. Interval]

-------------------+----------------------------------------------------------

**Kim TY [24] | .88123667 .77048073 1.0079137**

Mukai T [27] | .86651183 .75454797 .99508947

**Luo H [29] | .88316422 .77329038 1.0086496**

**Meng F [30] | .88620936 .77638986 1.0115627**

Ban Y [31] | .85924876 .74950691 .98505887

Sun L [33] | .86776176 .75491128 .99748208

Hsiao JY [35] | .86500054 .7541103 .99219695

**Su Y [36] | .89029158 .78223049 1.0132807**

**Ma L [37] | .87967894 .76949167 1.0056445**

**Yang J [38] | .87364174 .76129377 1.0025695**

Inoue N [39] | .86982209 .75976671 .99581945

**Huang J [40] | .88567069 .77610555 1.0107035**

**Chen X [41] | .87547374 .76371984 1.0035804**

Kim TY [24] | .86353434 .75345813 .98969209

Ban Y [31] | .8577196 .74907979 .98211555

Yang J [38] | .86486339 .75351948 .99266005

Inoue N [39] | .86343262 .75487262 .98760488

Joo YB [48] | .85371787 .74647749 .9763646

**Zhu Q [49] | .88700706 .77796731 1.0113298**

Wu C [50] | .8504098 .74667388 .96855783

Park JH [51] | .85732756 .74826197 .9822904

Hsieh YY [52] | .85889122 .75135075 .98182391

**Du J [53] | .88270059 .77257856 1.0085192**

Liu R [54] | .86161952 .75043753 .98927381

**Chen F [56] | .8705927 .75765264 1.0003683**

Tanizawa K [58] | .85902026 .75002317 .98385734

Chen F [59] | .86413796 .75328016 .99131035

Chen F [56] | .8624284 .74999906 .99171156

Pu T [60] | .86590841 .75594114 .9918727

**Wei Y [62] | .89628191 .78969725 1.0172522**

-------------------+----------------------------------------------------------

Combined | .87007895 .76191735 .99359513

4

------------------------------------------------------------------------------

Study ommited | e^coef. [95% Conf. Interval]

-------------------+----------------------------------------------------------

Kim TY [24] | .8482633 .73402606 .98027941

Mukai T [27] | .83058202 .71482083 .96509009

Luo H [29] | .84927179 .73538588 .98079471

Meng F [30] | .84812409 .73384047 .98020551

Ban Y [31] | .82754245 .71269251 .9609004

Sun L [33] | .83322531 .71647055 .96900622

Hsiao JY [35] | .82774998 .71342895 .96039001

Su Y [36] | .85697813 .74501068 .98577313

Ma L [37] | .84850287 .73484003 .97974674

Yang J [38] | .8383639 .7223596 .97299741

Inoue N [39] | .83750835 .72337334 .96965178

Huang J [40] | .8507652 .73707364 .98199337

Chen X [41] | .84405335 .72891715 .9773759

Kim TY [24] | .82643356 .71282058 .95815476

Ban Y [31] | .82295622 .70991386 .95399877

Yang J [38] | .82567782 .71138454 .95833382

Inoue N [39] | .82705831 .71464894 .95714891

Joo YB [48] | .81853769 .7067271 .94803773

Zhu Q [49] | .84708738 .73294711 .97900248

Wu C [50] | .81191212 .70560416 .93423667

Park JH [51] | .82199776 .70879461 .95328083

Hsieh YY [52] | .82382543 .71175055 .95354803

Du J [53] | .84361461 .72900541 .97624187

Liu R [54] | .82438238 .70988829 .95734261

Chen F [56] | .82579221 .71018968 .96021217

Tanizawa K [58] | .8226611 .70993122 .95329135

Chen F [59] | .82563672 .71160222 .9579453

Chen F [56] | .82438457 .70886302 .95873238

Pu T [60] | .83011675 .71628545 .962038

Wei Y [62] | .86230237 .75207641 .98868329

-------------------+----------------------------------------------------------

Combined | .83410884 .72218813 .9633744

5

------------------------------------------------------------------------------

Study ommited | e^coef. [95% Conf. Interval]

-------------------+----------------------------------------------------------

Kim TY [24] | .83709511 .71152224 .98482968

Mukai T [27] | .8171665 .68929752 .96875596

Luo H [29] | .8288458 .7016574 .97908945

Meng F [30] | .82053387 .69277423 .97185461

Ban Y [31] | .83380679 .70685032 .98356576

Sun L [33] | .82329659 .69438361 .97614239

Hsiao JY [35] | .81208135 .68691812 .96005055

Su Y [36] | .83482327 .70845239 .98373566

Ma L [37] | .85580426 .7357846 .99540127

Yang J [38] | .83122669 .70459255 .98062036

Inoue N [39] | .83156482 .70556831 .98006108

Huang J [40] | .82744036 .69977084 .97840251

Chen X [41] | .83412591 .70678327 .98441216

Kim TY [24] | .81218912 .68663426 .96070239

Ban Y [31] | .81792413 .69080306 .96843793

Yang J [38] | .80390931 .68160898 .94815385

Inoue N [39] | .81675036 .69117286 .96514372

Joo YB [48] | .80822689 .68090707 .95935369

Zhu Q [49] | .813793 .68816372 .96235681

Wu C [50] | .79981873 .67938024 .94160821

Park JH [51] | .81510673 .68847567 .965029

Hsieh YY [52] | .82120236 .69483228 .97055552

Du J [53] | .81395787 .6881294 .96279481

Liu R [54] | .80844652 .68306327 .95684513

Chen F [56] | .79507861 .68126446 .92790691

Tanizawa K [58] | .8129436 .68720448 .96168943

Chen F [59] | .80674492 .68234351 .95382656

Chen F [56] | .80538307 .68010601 .95373645

Pu T [60] | .81847471 .69235467 .96756891

Wei Y [62] | .83756574 .71130198 .98624267

-------------------+----------------------------------------------------------

Combined | .81978933 .69623697 .96526696

Overall- Asian - HWE-sub

1

------------------------------------------------------------------------------

Study ommited | e^coef. [95% Conf. Interval]

-------------------+----------------------------------------------------------

Kim TY [24] | .92009978 .82974648 1.0202919

Mukai T [27] | .90326843 .80946993 1.007936

Meng F [30] | .91304634 .82061412 1.0158899

Ban Y [31] | .90607042 .81260463 1.0102867

Sun L [33] | .90709937 .81287719 1.012243

Hsiao JY [35] | .89926201 .80678188 1.002343

Su Y [36] | .9248626 .8364209 1.022656

Yang J [38] | .91109357 .81815022 1.0145955

Inoue N [39] | .91185256 .82014616 1.0138133

Chen X [41] | .91775882 .82668591 1.0188649

Kim TY [24] | .89858616 .80650794 1.0011769

Ban Y [31] | .89878421 .8062804 1.0019009

**Yang J [38] | .89564721 .80383685 .99794371**

Inoue N [39] | .9002087 .8088606 1.0018731

**Joo YB [48] | .89311192 .80088711 .99595672**

Zhu Q [49] | .9101057 .81771656 1.0129334

**Wu C [50] | .88616674 .80001935 .98159061**

**Park JH [51] | .89696577 .80463024 .99989729**

Hsieh YY [52] | .90012858 .80844038 1.0022155

Du J [53] | .90815181 .81548293 1.0113513

Liu R [54] | .89668714 .80393891 1.0001355

**Chen F [56] | .89163883 .80107861 .99243669**

**Tanizawa K [58] | .89671441 .80491215 .99898695**

**Chen F [59] | .89642981 .80437456 .99902017**

Chen F [56] | .89588986 .80249268 1.000157

Pu T [60] | .902813 .81066555 1.0054348

Wei Y [62] | .92921232 .84297102 1.0242766

-------------------+----------------------------------------------------------

Combined | .90409307 .81402544 1.0041262

2

------------------------------------------------------------------------------

Study ommited | e^coef. [95% Conf. Interval]

-------------------+----------------------------------------------------------

Kim TY [24] | .84781473 .69293276 1.0373154

Mukai T [27] | .81807231 .66006154 1.013909

Meng F [30] | .8348834 .67737309 1.0290197

Ban Y [31] | .83140898 .6735816 1.026217

Sun L [33] | .8259251 .66657794 1.0233646

Hsiao JY [35] | .81045457 .6561543 1.0010399

Su Y [36] | .8520234 .69796545 1.0400857

Yang J [38] | .8373737 .68060474 1.0302525

Inoue N [39] | .83453283 .67858375 1.0263214

Chen X [41] | .84265556 .68618367 1.0348081

Kim TY [24] | .80961007 .65527594 1.0002938

Ban Y [31] | .81275306 .65694188 1.005519

**Yang J [38] | .80073402 .64957866 .98706287**

Inoue N [39] | .81226291 .65873561 1.0015718

**Joo YB [48] | .80241673 .64703166 .99511762**

Zhu Q [49] | .82575974 .66903318 1.0192008

**Wu C [50] | .78774057 .64551051 .96130923**

Park JH [51] | .81053879 .65511098 1.0028425

Hsieh YY [52] | .81489048 .66019633 1.0058318

Du J [53] | .82400769 .66728331 1.0175418

**Liu R [54] | .80553961 .65110158 .99660958**

**Chen F [56] | .79421188 .64637097 .97586763**

**Tanizawa K [58] | .80789404 .65401991 .99797081**

**Chen F [59] | .80450651 .65101169 .99419218**

**Chen F [56] | .80340268 .64815916 .99582928**

Pu T [60] | .81759054 .66221004 1.0094294

Wei Y [62] | .85874423 .70666234 1.0435559

-------------------+----------------------------------------------------------

Combined | .81938207 .66738022 1.0060037

3

------------------------------------------------------------------------------

Study ommited | e^coef. [95% Conf. Interval]

-------------------+----------------------------------------------------------

Kim TY [24] | .92501573 .80930482 1.0572705

Mukai T [27] | .90895815 .79118645 1.0442607

Meng F [30] | .93053161 .8162382 1.0608289

Ban Y [31] | .90027634 .78490666 1.0326037

Sun L [33] | .91072329 .79199504 1.0472501

Hsiao JY [35] | .90686014 .79030696 1.0406024

Su Y [36] | .93393241 .82192957 1.0611977

Yang J [38] | .91727434 .79939773 1.0525326

Inoue N [39] | .91145559 .7960487 1.0435936

Chen X [41] | .91897946 .80183372 1.0532399

Kim TY [24] | .90496873 .78935159 1.0375204

Ban Y [31] | .89834662 .78428156 1.0290012

Yang J [38] | .9068948 .78979336 1.0413587

Inoue N [39] | .90428438 .79049498 1.0344534

Joo YB [48] | .8934846 .78049871 1.0228264

Zhu Q [49] | .9305352 .81714843 1.0596554

Wu C [50] | .88948414 .7811819 1.0128013

Park JH [51] | .89800749 .78338308 1.0294037

Hsieh YY [52] | .89936754 .78670219 1.0281679

Du J [53] | .92611728 .81119849 1.0573161

Liu R [54] | .90327686 .78624227 1.0377324

Chen F [56] | .91403585 .79535954 1.05042

Tanizawa K [58] | .89983769 .78538503 1.0309693

Chen F [59] | .90591879 .78937497 1.0396692

Chen F [56] | .90452892 .78603712 1.0408829

Pu T [60] | .90738865 .79197447 1.0396221

Wei Y [62] | .94106788 .83134486 1.0652724

-------------------+----------------------------------------------------------

Combined | .91077776 .79762297 1.0399853

4

------------------------------------------------------------------------------

Study ommited | e^coef. [95% Conf. Interval]

-------------------+----------------------------------------------------------

Kim TY [24] | .90227039 .78323353 1.0393986

Mukai T [27] | .88226936 .76048367 1.0235581

Meng F [30] | .90218946 .78310242 1.0393862

Ban Y [31] | .87852715 .75769192 1.018633

Sun L [33] | .88571439 .76298047 1.0281914

Hsiao JY [35] | .87859141 .75840496 1.0178241

Su Y [36] | .91117758 .79544727 1.0437456

Yang J [38] | .89154231 .7699264 1.0323684

Inoue N [39] | .8888857 .76928048 1.0270867

Chen X [41] | .8979652 .77768808 1.0368444

Kim TY [24] | .87691862 .75755492 1.0150898

Ban Y [31] | .87295554 .75416546 1.0104565

Yang J [38] | .87628062 .75604806 1.0156335

Inoue N [39] | .87714368 .75928266 1.0132999

Joo YB [48] | .86759247 .74972894 1.0039851

Zhu Q [49] | .90022268 .78112955 1.0374731

**Wu C [50] | .85970417 .74893876 .98685139**

Park JH [51] | .87189981 .75285434 1.0097694

Hsieh YY [52] | .87369851 .7561568 1.0095116

Du J [53] | .89645335 .77659923 1.0348048

Liu R [54] | .8748562 .75431825 1.0146558

Chen F [56] | .87670073 .75488764 1.0181703

Tanizawa K [58] | .8725673 .75415943 1.009566

Chen F [59] | .87616803 .75625259 1.0150979

Chen F [56] | .87504366 .75324963 1.0165307

Pu T [60] | .88092425 .76141878 1.0191862

Wei Y [62] | .91818852 .80524001 1.04698

-------------------+----------------------------------------------------------

Combined | .88386924 .7667916 1.0188229

5

------------------------------------------------------------------------------

Study ommited | e^coef. [95% Conf. Interval]

-------------------+----------------------------------------------------------

Kim TY [24] | .89939311 .77094998 1.0492354

Mukai T [27] | .87738291 .74460033 1.0338443

Meng F [30] | .88130829 .74884795 1.0371989

Ban Y [31] | .89761467 .76727998 1.0500888

Sun L [33] | .88570316 .75229505 1.0427692

Hsiao JY [35] | .87065464 .74091542 1.0231121

Su Y [36] | .89763208 .76799871 1.0491468

Yang J [38] | .89297832 .76264665 1.0455829

Inoue N [39] | .89237871 .76283842 1.0439167

Chen X [41] | .90028002 .77006623 1.0525122

Kim TY [24] | .87087304 .74065133 1.0239904

Ban Y [31] | .87787372 .74600657 1.0330503

Yang J [38] | .86131807 .73489888 1.0094842

Inoue N [39] | .87568204 .74562826 1.02842

Joo YB [48] | .86642266 .73383285 1.022969

Zhu Q [49] | .87263513 .74238945 1.0257313

Wu C [50] | .85620423 .73202379 1.0014506

Park JH [51] | .87446728 .7430496 1.0291278

Hsieh YY [52] | .88088515 .7501028 1.0344698

Du J [53] | .87288623 .74240172 1.0263047

Liu R [54] | .86656518 .73639574 1.0197441

**Chen F [56] | .84963493 .73392136 .98359244**

Tanizawa K [58] | .87175316 .74133493 1.025115

Chen F [59] | .86450959 .73551123 1.0161325

Chen F [56] | .86275639 .73254624 1.0161114

Pu T [60] | .8778118 .7471826 1.0312788

Wei Y [62] | .9022843 .77322005 1.0528917

-------------------+----------------------------------------------------------

Combined | .87772089 .75025209 1.0268468

Overall- Asian - PB-sub

1

------------------------------------------------------------------------------

Study ommited | e^coef. [95% Conf. Interval]

-------------------+----------------------------------------------------------

Kim TY [24] | .85964236 .77059633 .95897809

Mukai T [27] | .84299066 .75139479 .94575218

Luo H [29] | .85891751 .76973284 .95843551

Meng F [30] | .85243388 .76163917 .95405219

Ban Y [31] | .84574033 .75431753 .94824351

Sun L [33] | .84631185 .75391502 .95003247

Hsiao JY [35] | .83955318 .74945447 .94048348

Su Y [36] | .86464505 .77725413 .9618618

Ma L [37] | .86176083 .77336465 .96026076

Yang J [38] | .85042966 .75919655 .9526263

Inoue N [39] | .85245104 .76259144 .95289921

Huang J [40] | .85896364 .76972232 .95855158

Kim TY [24] | .83903019 .74932984 .93946835

Ban Y [31] | .83906086 .74897216 .93998572

Yang J [38] | .83609578 .74686556 .9359866

Inoue N [39] | .84117915 .75204679 .94087544

Joo YB [48] | .83360936 .74514543 .93257576

Zhu Q [49] | .85019608 .75971079 .95145861

Park JH [51] | .83728013 .74748335 .93786438

Hsieh YY [52] | .84086249 .75142802 .94094138

Du J [53] | .84818647 .75750213 .94972708

Chen F [56] | .83229705 .74494546 .9298914

Tanizawa K [58] | .83720119 .74787217 .93720005

Chen F [59] | .83682111 .74730908 .9370548

Chen F [56] | .83603709 .74555794 .93749656

Pu T [60] | .84337029 .75340546 .94407792

Wei Y [62] | .86830667 .78258675 .96341585

-------------------+----------------------------------------------------------

Combined | .84637941 .75831272 .94467373

2

------------------------------------------------------------------------------

Study ommited | e^coef. [95% Conf. Interval]

-------------------+----------------------------------------------------------

Kim TY [24] | .73978946 .59607709 .91815045

Mukai T [27] | .7113356 .56641753 .89333099

Luo H [29] | .73147862 .58657886 .91217229

Meng F [30] | .72656994 .58116394 .90835621

Ban Y [31] | .72339536 .57789661 .90552677

Sun L [33] | .71760701 .57083601 .90211518

Hsiao JY [35] | .70566543 .56430682 .88243432

Su Y [36] | .74324408 .59998041 .92071633

Ma L [37] | .75685609 .61744135 .92774989

Yang J [38] | .72964271 .58483239 .91030951

Inoue N [39] | .72842872 .58446315 .90785603

Huang J [40] | .73136154 .58620355 .91246411

Kim TY [24] | .70478374 .56346413 .88154703

Ban Y [31] | .70723543 .5645244 .88602362

Yang J [38] | .69700055 .55892676 .86918322

Inoue N [39] | .70807121 .56717535 .88396796

Joo YB [48] | .69777664 .55688802 .87430906

Zhu Q [49] | .71941275 .57522236 .89974719

Park JH [51] | .70523298 .56299012 .88341436

Hsieh YY [52] | .71005964 .56809191 .8875055

Du J [53] | .71772377 .5736221 .89802573

Chen F [56] | .6913956 .55767252 .85718383

Tanizawa K [58] | .7032493 .56242204 .8793389

Chen F [59] | .70000431 .55980691 .87531259

Chen F [56] | .69861789 .55745966 .87551979

Pu T [60] | .71237558 .56970673 .89077228

Wei Y [62] | .74815126 .6060184 .92361933

-------------------+----------------------------------------------------------

Combined | .71714512 .57703176 .89128045

3

------------------------------------------------------------------------------

Study ommited | e^coef. [95% Conf. Interval]

-------------------+----------------------------------------------------------

Kim TY [24] | .85639813 .74305864 .98702541

Mukai T [27] | .83992002 .72543493 .97247267

Luo H [29] | .85873952 .7464246 .98795453

Meng F [30] | .86195108 .74963453 .99109583

Ban Y [31] | .83216975 .72042405 .96124845

Sun L [33] | .84113093 .72559661 .97506138

Hsiao JY [35] | .83848779 .72524822 .96940848

Su Y [36] | .86674434 .75648274 .99307719

Ma L [37] | .85494019 .74229686 .98467711

Yang J [38] | .84769463 .73259834 .98087335

Inoue N [39] | .84425543 .73184749 .97392863

Huang J [40] | .861478 .74949063 .9901983

Kim TY [24] | .83705621 .72475797 .96675459

Ban Y [31] | .83067891 .72020039 .95810479

Yang J [38] | .83821175 .72447143 .96980902

Inoue N [39] | .83747319 .72684101 .96494465

Joo YB [48] | .82614811 .71856605 .94983711

Zhu Q [49] | .8630335 .75165427 .99091678

Park JH [51] | .83008495 .71919888 .95806743

Hsieh YY [52] | .83244709 .72308875 .95834455

Du J [53] | .85817146 .74556809 .98778136

Chen F [56] | .84422327 .72850206 .97832657

Tanizawa K [58] | .83211723 .72117659 .96012417

Chen F [59] | .83751405 .72433609 .96837613

Chen F [56] | .83528608 .72045022 .96842616

Pu T [60] | .83983302 .72759254 .96938803

Wei Y [62] | .87347266 .76485374 .99751683

-------------------+----------------------------------------------------------

Combined | .8451396 .73484209 .97199243

4

------------------------------------------------------------------------------

Study ommited | e^coef. [95% Conf. Interval]

-------------------+----------------------------------------------------------

Kim TY [24] | .82464748 .71021004 .95752444

Mukai T [27] | .80487268 .68881086 .94049045

Luo H [29] | .82578731 .71177911 .95805659

Meng F [30] | .824489 .7099945 .957447

Ban Y [31] | .80163804 .68667095 .93585368

Sun L [33] | .80765455 .69041065 .94480852

Hsiao JY [35] | .80200936 .68760756 .93544495

Su Y [36] | .83445581 .72274717 .96343028

Ma L [37] | .8249494 .71121521 .95687143

Yang J [38] | .81345481 .69699003 .94938047

Inoue N [39] | .81298539 .69866246 .94601511

Huang J [40] | .82746774 .71367824 .95939993

Kim TY [24] | .80072285 .6871253 .93310068

Ban Y [31] | .79684069 .68401463 .92827704

Yang J [38] | .79964389 .68535385 .93299302

Inoue N [39] | .80195675 .68962151 .93259073

Joo YB [48] | .79191414 .68189954 .91967801

Zhu Q [49] | .82336003 .70905493 .95609198

Park JH [51] | .79565032 .682748 .92722269

Hsieh YY [52] | .79829233 .686423 .92839349

Du J [53] | .8195018 .70464452 .95308086

Chen F [56] | .79953581 .68385218 .93478903

Tanizawa K [58] | .79660354 .68411692 .92758589

Chen F [59] | .79966541 .68565162 .93263801

Chen F [56] | .79797325 .68249503 .9329904

Pu T [60] | .80487337 .6909459 .93758592

Wei Y [62] | .84077163 .73110956 .96688235

-------------------+----------------------------------------------------------

Combined | .80998652 .69808241 .93982909

5

------------------------------------------------------------------------------

Study ommited | e^coef. [95% Conf. Interval]

-------------------+----------------------------------------------------------

Kim TY [24] | .8195176 .69095617 .97199957

Mukai T [27] | .79672371 .66551315 .95380336

Luo H [29] | .8100366 .67955162 .96557682

Meng F [30] | .80056136 .66944161 .9573628

Ban Y [31] | .81565364 .68544355 .97059904

Sun L [33] | .80347937 .67100312 .96211043

Hsiao JY [35] | .79149623 .66343924 .94427076

Su Y [36] | .81687584 .68736029 .97079529

Ma L [37] | .84170226 .71994777 .98404735

Yang J [38] | .81280173 .68296732 .96731811

Inoue N [39] | .81327821 .68419019 .96672161

Huang J [40] | .80836595 .6773207 .96476529

Kim TY [24] | .79151437 .66301633 .94491637

Ban Y [31] | .79773043 .66736812 .95355744

Yang J [38] | .78261744 .6580328 .93078956

Inoue N [39] | .79678128 .66818189 .95013114

Joo YB [48] | .78664878 .656374 .94278003

Zhu Q [49] | .79335577 .66474205 .94685356

Park JH [51] | .79461963 .66485243 .94971505

Hsieh YY [52] | .80163231 .67210156 .95612686

Du J [53] | .79349303 .6646523 .94730912

Chen F [56] | .77324224 .65972741 .9062888

Tanizawa K [58] | .7923422 .6636212 .9460309

Chen F [59] | .78541396 .65838568 .93695096

Chen F [56] | .78363415 .65582457 .93635174

Pu T [60] | .79860163 .66937313 .95277885

Wei Y [62] | .82001057 .69062543 .97363535

-------------------+----------------------------------------------------------

Combined | .80073768 .67440075 .95074156

*（2）Publication bias results：*

Overall

1

Begg's Test

adj. Kendall's Score (P-Q) = 23

Std. Dev. of Score = 123.12

Number of Studies = 51

z = 0.19

Pr > |z| = 0.852

z = 0.18 (continuity corrected)

Pr > |z| = 0.858 (continuity corrected)

Egger's test

------------------------------------------------------------------------------

Std_Eff | Coef. Std. Err. t P>|t| [95% Conf. Interval]

-------------+----------------------------------------------------------------

slope | 5.126671 .0533887 96.03 0.000 5.019383 5.23396

bias | 3.354272 3.788793 0.89 0.380 -4.259593 10.96814

2

Begg's Test

adj. Kendall's Score (P-Q) = -166

Std. Dev. of Score = 116.01

Number of Studies = 49

z = -1.43

Pr > |z| = 0.152

z = 1.42 (continuity corrected)

Pr > |z| = 0.155 (continuity corrected)

Egger's test

------------------------------------------------------------------------------

Std_Eff | Coef. Std. Err. t P>|t| [95% Conf. Interval]

-------------+----------------------------------------------------------------

slope | 3.475477 .1205008 28.84 0.000 3.233061 3.717893

bias | 1.067573 .5671554 1.88 0.066 -.0733968 2.208542

3

Begg's Test

adj. Kendall's Score (P-Q) = 249

Std. Dev. of Score = 123.12

Number of Studies = 51

z = 2.02

Pr > |z| = **0.043**

z = 2.01 (continuity corrected)

Pr > |z| = 0.044 (continuity corrected)

Egger's test

------------------------------------------------------------------------------

Std_Eff | Coef. Std. Err. t P>|t| [95% Conf. Interval]

-------------+----------------------------------------------------------------

slope | 5.468241 .095362 57.34 0.000 5.276604 5.659878

bias | -.7267067 1.683857 -0.43 0.668 -4.110544 2.657131

4

Begg's Test

adj. Kendall's Score (P-Q) = -675

Std. Dev. of Score = 123.12

Number of Studies = 51

z = -5.48

Pr > |z| = **0.000**

z = 5.47 (continuity corrected)

Pr > |z| = 0.000 (continuity corrected)

Egger's test

------------------------------------------------------------------------------

Std_Eff | Coef. Std. Err. t P>|t| [95% Conf. Interval]

-------------+----------------------------------------------------------------

slope | 4.57218 .0614552 74.40 0.000 4.448681 4.695679

bias | -.7331507 2.708873 -0.27 0.788 -6.176834 4.710533

5

Begg's Test

adj. Kendall's Score (P-Q) = -113

Std. Dev. of Score = 119.55

Number of Studies = 50

z = -0.95

Pr > |z| = 0.345

z = 0.94 (continuity corrected)

Pr > |z| = 0.349 (continuity corrected)

Egger's test

------------------------------------------------------------------------------

Std_Eff | Coef. Std. Err. t P>|t| [95% Conf. Interval]

-------------+----------------------------------------------------------------

slope | 3.218062 .1257128 25.60 0.000 2.9653 3.470825

bias | .8238879 .3455079 2.38 **0.021** **.1291978 1.518578**

------------------------------------------------------------------------------

MS

1

Begg's Test

adj. Kendall's Score (P-Q) = 0

Std. Dev. of Score = 4.08

Number of Studies = 5

z = 0.00

Pr > |z| = 1.000

z = -0.24 (continuity corrected)

Pr > |z| = 1.000 (continuity corrected)

Egger's test

------------------------------------------------------------------------------

Std_Eff | Coef. Std. Err. t P>|t| [95% Conf. Interval]

-------------+----------------------------------------------------------------

slope | 4.739003 .8745782 5.42 0.012 1.955705 7.522301

bias | 11.01722 6.36612 1.73 0.182 -9.242612 31.27706

2

Begg's Test

adj. Kendall's Score (P-Q) = 2

Std. Dev. of Score = 4.08

Number of Studies = 5

z = 0.49

Pr > |z| = 0.624

z = 0.24 (continuity corrected)

Pr > |z| = 0.806 (continuity corrected)

Egger's test

------------------------------------------------------------------------------

Std_Eff | Coef. Std. Err. t P>|t| [95% Conf. Interval]

-------------+----------------------------------------------------------------

slope | 1.852588 .7247682 2.56 0.083 -.4539483 4.159123

bias | 4.386811 2.725424 1.61 0.206 -4.286705 13.06033

3

Begg's Test

adj. Kendall's Score (P-Q) = -2

Std. Dev. of Score = 4.08

Number of Studies = 5

z = -0.49

Pr > |z| = 0.624

z = 0.24 (continuity corrected)

Pr > |z| = 0.806 (continuity corrected)

Egger's test

------------------------------------------------------------------------------

Std_Eff | Coef. Std. Err. t P>|t| [95% Conf. Interval]

-------------+----------------------------------------------------------------

slope | 5.564877 .4450155 12.50 0.001 4.14864 6.981115

bias | -2.807635 3.341538 -0.84 0.462 -13.4419 7.826629

4

Begg's Test

adj. Kendall's Score (P-Q) = 2

Std. Dev. of Score = 4.08

Number of Studies = 5

z = 0.49

Pr > |z| = 0.624

z = 0.24 (continuity corrected)

Pr > |z| = 0.806 (continuity corrected)

Egger's test

------------------------------------------------------------------------------

Std_Eff | Coef. Std. Err. t P>|t| [95% Conf. Interval]

-------------+----------------------------------------------------------------

slope | 5.371627 .34008 15.80 0.001 4.289341 6.453914

bias | -1.638699 2.956723 -0.55 0.618 -11.04831 7.770912

5

Begg's Test

adj. Kendall's Score (P-Q) = 2

Std. Dev. of Score = 4.08

Number of Studies = 5

z = 0.49

Pr > |z| = 0.624

z = 0.24 (continuity corrected)

Pr > |z| = 0.806 (continuity corrected)

Egger's test

------------------------------------------------------------------------------

Std_Eff | Coef. Std. Err. t P>|t| [95% Conf. Interval]

-------------+----------------------------------------------------------------

slope | 1.867325 .4652659 4.01 0.028 .3866415 3.348009

bias | 3.752965 1.002717 3.74 **0.033** **.5618722 6.944058**

GD

1

Begg's Test

adj. Kendall's Score (P-Q) = -1

Std. Dev. of Score = 28.58

Number of Studies = 19

z = -0.03

Pr > |z| = 0.972

z = 0.00 (continuity corrected)

Pr > |z| = 1.000 (continuity corrected)

Egger's test

------------------------------------------------------------------------------

Std_Eff | Coef. Std. Err. t P>|t| [95% Conf. Interval]

-------------+----------------------------------------------------------------

slope | 4.895329 .0911284 53.72 0.000 4.703065 5.087593

bias | 6.1788 4.006841 1.54 0.141 -2.274895 14.63249

2

Begg's Test

adj. Kendall's Score (P-Q) = -32

Std. Dev. of Score = 24.28

Number of Studies = 17

z = -1.32

Pr > |z| = 0.187

z = 1.28 (continuity corrected)

Pr > |z| = 0.202 (continuity corrected)

Egger's test

------------------------------------------------------------------------------

Std_Eff | Coef. Std. Err. t P>|t| [95% Conf. Interval]

-------------+----------------------------------------------------------------

slope | 3.51659 .1220167 28.82 0.000 3.256518 3.776663

bias | .3583172 .3624959 0.99 0.339 -.4143244 1.130959

3

Begg's Test

adj. Kendall's Score (P-Q) = -41

Std. Dev. of Score = 28.58

Number of Studies = 19

z = -1.43

Pr > |z| = 0.151

z = 1.40 (continuity corrected)

Pr > |z| = 0.162 (continuity corrected)

Egger's test

------------------------------------------------------------------------------

Std_Eff | Coef. Std. Err. t P>|t| [95% Conf. Interval]

-------------+----------------------------------------------------------------

slope | 4.707637 .1145671 41.09 0.000 4.465922 4.949352

bias | .7020401 1.514222 0.46 0.649 -2.492689 3.89677

4

Begg's Test

adj. Kendall's Score (P-Q) = -51

Std. Dev. of Score = 28.58

Number of Studies = 19

z = -1.78

Pr > |z| = 0.074

z = 1.75 (continuity corrected)

Pr > |z| = 0.080 (continuity corrected)

Egger's test

------------------------------------------------------------------------------

Std_Eff | Coef. Std. Err. t P>|t| [95% Conf. Interval]

-------------+----------------------------------------------------------------

slope | 4.389629 .0551816 79.55 0.000 4.273206 4.506052

bias | 1.093729 3.460433 0.32 0.756 -6.207147 8.394605

5

Begg's Test

adj. Kendall's Score (P-Q) = -47

Std. Dev. of Score = 26.40

Number of Studies = 18

z = -1.78

Pr > |z| = 0.075

z = 1.74 (continuity corrected)

Pr > |z| = 0.081 (continuity corrected)

Egger's test

------------------------------------------------------------------------------

Std_Eff | Coef. Std. Err. t P>|t| [95% Conf. Interval]

-------------+----------------------------------------------------------------

slope | 3.430886 .1551424 22.11 0.000 3.101999 3.759773

bias | -.1472584 .4207599 -0.35 0.731 -1.03923 .7447127

GD’s Asian subgroup

1

Begg's Test

adj. Kendall's Score (P-Q) = 0

Std. Dev. of Score = 16.39

Number of Studies = 13

z = 0.00

Pr > |z| = 1.000

z = -0.06 (continuity corrected)

Pr > |z| = 1.000 (continuity corrected)

Egger's test

------------------------------------------------------------------------------

Std_Eff | Coef. Std. Err. t P>|t| [95% Conf. Interval]

-------------+----------------------------------------------------------------

slope | 4.898436 .1091495 44.88 0.000 4.6582 5.138673

bias | 9.041002 5.796091 1.56 0.147 -3.716108 21.79811

2

Begg's Test

adj. Kendall's Score (P-Q) = -8

Std. Dev. of Score = 14.58

Number of Studies = 12

z = -0.55

Pr > |z| = 0.583

z = 0.48 (continuity corrected)

Pr > |z| = 0.631 (continuity corrected)

Egger's test

------------------------------------------------------------------------------

Std_Eff | Coef. Std. Err. t P>|t| [95% Conf. Interval]

-------------+----------------------------------------------------------------

slope | 3.668067 .08803 41.67 0.000 3.471924 3.864211

bias | .8621797 .2968705 2.90 **0.016 .2007111 1.523648**

3

Begg's Test

adj. Kendall's Score (P-Q) = -18

Std. Dev. of Score = 16.39

Number of Studies = 13

z = -1.10

Pr > |z| = 0.272

z = 1.04 (continuity corrected)

Pr > |z| = 0.300 (continuity corrected)

Egger's test

------------------------------------------------------------------------------

Std_Eff | Coef. Std. Err. t P>|t| [95% Conf. Interval]

-------------+----------------------------------------------------------------

slope | 4.725062 .1364201 34.64 0.000 4.424803 5.025321

bias | .0689683 1.884458 0.04 0.971 -4.078695 4.216632

4

Begg's Test

adj. Kendall's Score (P-Q) = 0

Std. Dev. of Score = 16.39

Number of Studies = 13

z = 0.00

Pr > |z| = 1.000

z = -0.06 (continuity corrected)

Pr > |z| = 1.000 (continuity corrected)

Egger's test

------------------------------------------------------------------------------

Std_Eff | Coef. Std. Err. t P>|t| [95% Conf. Interval]

-------------+----------------------------------------------------------------

slope | 5.152462 .0797311 64.62 0.000 4.976975 5.327949

bias | -.1792999 1.378619 -0.13 0.899 -3.21362 2.855021

5

Begg's Test

adj. Kendall's Score (P-Q) = -28

Std. Dev. of Score = 16.39

Number of Studies = 13

z = -1.71

Pr > |z| = 0.088

z = 1.65 (continuity corrected)

Pr > |z| = 0.100 (continuity corrected)

Egger's test

------------------------------------------------------------------------------

Std_Eff | Coef. Std. Err. t P>|t| [95% Conf. Interval]

-------------+----------------------------------------------------------------

slope | 3.477488 .1325329 26.24 0.000 3.185785 3.769191

bias | .2786498 .4062413 0.69 0.507 -.6154812 1.172781

GD’s Caucasian subgroup

1

Begg's Test

adj. Kendall's Score (P-Q) = -1

Std. Dev. of Score = 5.32

Number of Studies = 6

z = -0.19

Pr > |z| = 0.851

z = 0.00 (continuity corrected)

Pr > |z| = 1.000 (continuity corrected)

Egger's test

------------------------------------------------------------------------------

Std_Eff | Coef. Std. Err. t P>|t| [95% Conf. Interval]

-------------+----------------------------------------------------------------

slope | 5.400739 1.01289 5.33 0.006 2.588507 8.212971

bias | -1.687659 3.566625 -0.47 0.661 -11.5902 8.214879

2

Begg's Test

adj. Kendall's Score (P-Q) = 0

Std. Dev. of Score = 4.08

Number of Studies = 5

z = 0.00

Pr > |z| = 1.000

z = -0.24 (continuity corrected)

Pr > |z| = 1.000 (continuity corrected)

Egger's test

------------------------------------------------------------------------------

Std_Eff | Coef. Std. Err. t P>|t| [95% Conf. Interval]

-------------+----------------------------------------------------------------

slope | 1.967557 .601362 3.27 0.047 .0537548 3.881359

bias | 1.685752 .9906331 1.70 0.187 -1.466885 4.838388

3

Begg's Test

adj. Kendall's Score (P-Q) = 3

Std. Dev. of Score = 5.32

Number of Studies = 6

z = 0.56

Pr > |z| = 0.573

z = 0.38 (continuity corrected)

Pr > |z| = 0.707 (continuity corrected)

Egger's test

------------------------------------------------------------------------------

Std_Eff | Coef. Std. Err. t P>|t| [95% Conf. Interval]

-------------+----------------------------------------------------------------

slope | 4.377814 .327143 13.38 0.000 3.46952 5.286109

bias | 4.950447 3.867187 1.28 0.270 -5.786586 15.68748

4

Begg's Test

adj. Kendall's Score (P-Q) = -1

Std. Dev. of Score = 5.32

Number of Studies = 6

z = -0.19

Pr > |z| = 0.851

z = 0.00 (continuity corrected)

Pr > |z| = 1.000 (continuity corrected)

Egger's test

------------------------------------------------------------------------------

Std_Eff | Coef. Std. Err. t P>|t| [95% Conf. Interval]

-------------+----------------------------------------------------------------

slope | 4.378143 .0356335 122.87 0.000 4.279209 4.477078

bias | 11.25472 3.871646 2.91 **0.044 .5053078 22.00413**

5

Begg's Test

adj. Kendall's Score (P-Q) = -2

Std. Dev. of Score = 4.08

Number of Studies = 5

z = -0.49

Pr > |z| = 0.624

z = 0.24 (continuity corrected)

Pr > |z| = 0.806 (continuity corrected)

Egger's test

------------------------------------------------------------------------------

Std_Eff | Coef. Std. Err. t P>|t| [95% Conf. Interval]

-------------+----------------------------------------------------------------

slope | 3.122365 4.253141 0.73 0.516 -10.41303 16.65776

bias | -.8759348 6.091301 -0.14 0.895 -20.26117 18.5093
